# Identification of glutathione (GSH)-independent glyoxalase III from *Schizosaccharomyces pombe*

**DOI:** 10.1186/1471-2148-14-86

**Published:** 2014-04-23

**Authors:** Qiaoqiao Zhao, Yang Su, Zhikang Wang, Caiping Chen, Tongsiyu Wu, Ying Huang

**Affiliations:** 1 Jiangsu Key Laboratory for Microbes and Genomics, School of Life Sciences, Nanjing Normal University, 1 Wenyuan Road, Nanjing 210023, China

**Keywords:** Glyoxalase III, DJ-1, Hsp31, Methylglyoxal, Reactive carbonyl species

## Abstract

**Background:**

Reactive carbonyl species (RCS), such as methylglyoxal (MG) and glyoxal (GO), are synthesized as toxic metabolites in living systems. Mechanisms of RCS detoxification include the glutathione (GSH)-dependent system consisting of glyoxalase I (GLO1) and glyoxalase II (GLO2), and GSH-independent system involving glyoxalase III (GLO3). Hsp31 and DJ-1 proteins are weakly homologous to each other and belong to two different subfamilies of the DJ-1/Hsp31/PfpI superfamily. Recently, the *Escherichia coli* Hsp31 protein and the DJ-1 proteins from *Arabidopsis thaliana* and metazoans have been demonstrated to have GLO3 activity.

**Results:**

We performed a systematic survey of homologs of DJ-1 and Hsp31 in fungi. We found that DJ-1 proteins have a very limited distribution in fungi, whereas Hsp31 proteins are widely distributed among different fungal groups. Phylogenetic analysis revealed that fungal and metazoan DJ-1 proteins and bacterial YajL proteins are most closely related and together form a sister clade to bacterial and fungal Hsp31 proteins. We showed that two *Schizosaccharomyces pombe* Hsp31 proteins (Hsp3101 and Hsp3102) and one *Saccharomyces cerevisiae* Hsp31 protein (ScHsp31) displayed significantly higher *in vitro* GLO3 activity than *S. pombe* DJ-1 (SpDJ-1). Overexpression of *hsp3101*, *hsp3102* and *ScHSP31* could confer MG and GO resistance on either wild-type *S. pombe* cells or *GLO1* deletion of *S. pombe. S. pombe* DJ-1 and Hsp31 proteins exhibit different patterns of subcellular localization.

**Conclusions:**

Our results suggest that fungal Hsp31 proteins are the major GLO3 that may have some role in protecting cells from RCS toxicity in fungi. Our results also support the view that the GLO3 activity of Hsp31 proteins may have evolved independently from that of DJ-1 proteins.

## Background

Reactive carbonyl species (RCS) are usually produced as metabolites in living systems including prokaryotes, fungi, plants, and animals. Among RCS are the highly reactive α-carbonyl compounds methylglyoxal (MG) and glyoxal (GO). MG is formed primarily as a by-product of glycolysis via the *β*-elimination of phosphate from the glycolytic intermediates dihydroxyacetone phosphate (DHAP) and glyceraldehyde 3-phosphate (GA3P). GO is generated by oxidative degradation of lipid and DNA, and oxidation of glycolaldehyde. RCS can rapidly react with the amino groups in proteins, nucleic acids and lipids to form toxic or mutagenic advanced glycation endproducts (AGE) and cause carbonyl stress. RCS have been linked to diverse human diseases, including diabetes, neurodegenerative diseases (such as Parkinson and Alzheimer), and aging (for reviews, see [1-3]).

The major system for reactive α-carbonyl species detoxification in both prokaryotes and eukaryotes involves two enzymes, glyoxalase I (GLO1, EC 4.4.1.5) and glyoxalase II (GLO2, EC 3.1.2.6) (for reviews, see [4,5]). GLO1 converts MG into *S*-D-lactoylglutathione (SLG) with glutathione (GSH) as a catalytic cofactor, and then GLO2 hydrolyzes SLG to D-lactate and GSH. Most recently, a GSH-independent glyoxalase system was identified in *Escherichia coli*, *Caenorhabditis elegans*, mice and humans [6,7]. In this system, glyoxalase III (GLO3) converts MG directly into D-lactate in a single step, independent of GSH.

In animals, GLO3 activity appears to reside in DJ-1 proteins [6]. The GLO3 activity of DJ-1 proteins seems to play an important role in protecting cells against α-dicarbonyl-induced cell death [6]. Human DJ-1 (HsDJ-1) is a cancer- and Parkinson’s disease (PD)-associated protein [8,9]. Besides playing a role in α-dicarbonyl detoxification, HsDJ-1 has multiple functions, including transcriptional regulation [10-19], regulation of mitochondrial function [20-25], molecular chaperone [26,27] and protease activities [28-30], and, most strikingly, protection against oxidative stress [31-36]. It appears that oxidation at the conserved cysteine residue (Cys106 in HsDJ-1) is critically required for DJ-1 function [22,37-39]. However, the mechanism of how HsDJ-1 executes these functions remains to be determined.

The closely related homologs of HsDJ-1 (hereafter referred to as DJ-1 proteins) are found in a wide variety of eukaryotes [40-43], but the species distribution of this gene appears to be complex. Unlike mammals, which contain a single *DJ*-*1* gene, *Drosophila melanogaster* has two *DJ*-*1* genes (*DJ*-*1α* and *DJ*-*1β*) [44], which differ in tissue distribution. *DJ*-*1α* is detected primarily in the testes from the pupal to the adult stage, whereas *DJ*-*1β* is found in most tissues from embryo to adults [33,44,45]. Similar to *Drosophila*, *C. elegans* has two *DJ*-*1* genes (*cDJR*-*1.1* and *cDJR*-*1.2*), both of which encode GLO3 [6]. *cDJR*-*1.1* is found in both the nucleus and cytoplasm of the intestine, while *cDJR*-*1.2* is detected in various tissues and is only present in the cytoplasm [6]. The *Arabidopsis thaliana* genome has six *DJ*-*1* genes, among which *AtDJ*-*1d* encodes an enzyme that has the highest GLO3 activity [46]. Besides, a close homolog of HsDJ-1 called SpDJ-1 (Accession number: SPAC22E12.03c) has been found in the fission yeast *Schizosaccharomyces pombe*[41].

The most closely related homologs of DJ-1 in prokaryotes are YajL proteins. The best characterized YajL protein is the *E. coli* YajL protein (EcYajL). Like DJ-1, EcYajL protects cells against oxidative stress [47]. The conserved cysteine 106 of EcYajL can form mixed disulfides with cytoplasmic proteins including chaperones, proteases, ribosomal proteins, catalases, peroxidases and FeS proteins. This covalent chaperone activity of EcYajL is required to protect against protein aggregation and sulfenylation during oxidative stress [48-50]. However, unlike DJ-1, EcYajL does not display any activity to MG [6].

The principal GLO3 enzyme responsible for converting MG to D-lactate without a cofactor in *E. coli* is EcHsp31 (formerly known as YedU) [7], which shares only 14% sequence identity to HsDJ-1. It has been suggested that EcHsp31 is involved in protecting stationary cells against dicarbonyl stress [7]. In addition, EcHsp31 is a heat-inducible molecular chaperone [51,52] that plays a role in protecting cells against multiple stresses including heat shock and starvation [53-55]. EcHsp31 is also involved in the management of protein misfolding under acid stress [56]. However, unlike DJ-1 proteins, EcHsp31 does not appear to be involved in oxidative stress response [56].

Close homologs of EcHsp31 (hereafter referred to as Hsp31 proteins) have been found in bacteria and fungi [41,55,57]. ScHsp31 (Accession number: YDR533C), a *Saccharomyces cerevisiae* homolog of EcHsp31, is involved in the protection of yeast cells against reactive oxygen species [58].

Based on sequence comparison, DJ-1 and Hsp31 proteins belong to different subfamilies of the DJ-1/Hsp31/PfpI superfamily, which encompasses a wide variety of functionally diverse proteins [40,41,43]. Both DJ-1 and Hsp31 proteins possess the Glu-Cys-His catalytic triad. The first two triad residues (e.g., Glu16 and Cys111 in SpDJ-1, and Glu30 and Cys138 in ScHsp31) are in the same locations in the two enzymes and are essential for GLO3 activity [6,7]. In contrast, the third triad residue His (e.g., His130 in SpDJ-1 and His139 in ScHsp31) resides at different sequence and structural positions, and is less important for enzyme activity [6,7]. In addition, Hsp31 proteins also have a potential cysteine protease-like catalytic triad (Cys138-His139-Glu170 in ScHsp31) of unknown functions [57,59-61].

Structural and bioinformatic studies reveal that Hsp31 proteins but not DJ-1, YajL and PfpI proteins have a P domain, which is likely involved in Hsp31 dimer formation and substrate binding [57,60]. Furthermore, Hsp31 proteins can clearly be divided into class I and class II proteins based on their structural and sequence similarity [57]. Class I proteins represented by EcHsp31 contain a complete P domain, whereas class II proteins represented by ScHsp31 contain a shorter P domain and have Glu instead of Asp as the third member of a cysteine protease-like catalytic triad [57,60].

To better understand the origins and functions of GLO3, we investigated the distribution and diversity of DJ-1 and Hsp31 proteins in a broad diversity of fungal species since GLO3 activity is most likely to reside in these proteins. This is the first comprehensive survey of DJ-1 and Hsp31 proteins in fungi. We also provide evidence that fungal homologs of the DJ-1/Hsp31/PfpI superfamily may function as GLO3.

## Results

### Candidate DJ-1 proteins are present only in a very limited number of fungal species

To determine the distribution of DJ-1 proteins in fungi, we performed extensive BLAST searches against fungal databases using HsDJ-1 as a query sequence. In total, we have examined 191 sequenced fungal species, including 134 Ascomycota, 39 Basidiomycota, 3 Zygomycota, 3 Chytridiomycota and 12 Microsporidia. We identified 46 candidate DJ-1 proteins from a total of 43 fungal species (Table 1). Among them, only four (*S. pombe*, *Alternaria brassicicola*, *Ustilago maydis* and *Coprinopsis cinerea*) have been previously reported [41]. Identical results were obtained when EcYajL was used as a query sequence. However, no DJ-1 protein was found by the BLAST search by using EcHsp31 as a query.

**Table 1:** **Candidate fungal DJ**-**1 proteins identified in this study**

**Species#**	**Taxonomy**	**Accession number/name**	**No. aa**^+^	**E value**	**Database**
*Alternaria arborescens*	Ascomycota	-	199	4E-24	NCBI
*Alternaria brassicicola*	Ascomycota	Altbr1_7229	197	2.32E-26	JGI
*Cochliobolus heterostrophus*	Ascomycota	CocheC4_1_31923	197	4.97E-28	JGI
*Cochliobolus lunatus*	Ascomycota	Coclu2_103587	196	1.04E-26	JGI
*Cochliobolus miyabeanus*	Ascomycota	Cocmi1_87889	197	7.63E-27	JGI
*Cochliobolus sativus*	Ascomycota	Cocsa1_181323	197	2.67E-26	JGI
*Cochliobolus victoriae*	Ascomycota	Cocvi1_89530	197	1.25E-27	JGI
*Pyrenophora teres*	Ascomycota	PTT_13806	197	8.00E-34	NCBI
*Pyrenophora tritici*	Ascomycota	PTRG_06163.1	197	2.67E-28	Broad
*Phaeosphaeria nodorum*	Ascomycota	SNOG_07399	193	1.32E-27	Broad
*Schizosaccharomyces japonicus*	Ascomycota	SJAG_06414.4	191	5.77E-15	Broad
*Schizosaccharomyces japonicus*	Ascomycota	SJAG_02106.4	199	7.30E-10	Broad
*Schizosaccharomyces pombe*	Ascomycota	SPAC22E12.03c/SpDJ-1	191	4.89E-14	Broad
*Schizosaccharomyces octosporus*	Ascomycota	SOCG_00579.5	202	3.50E-12	Broad
*Schizosaccharomyces cryophilus*	Ascomycota	SPOG_01926.3	202	7.80E-12	Broad
*Agaricus bisporus*	Basidiomycota	Agabi_187606	200	1.06E-18	JGI
*Auricularia delicate*	Basidiomycota	Aurde1_110250	191	4.77E-21	JGI
*Ceriporiopsis subvermispora*	Basidiomycota	Cersu1_140432	200	1.63E-20	JGI
*Coprinopsis cinerea*	Basidiomycota	CC1G_10336.3	197	3.26E-26	Broad
*Dichomitus squalens*	Basidiomycota	Dicsq1_101983	202	5.63E-20	JGI
*Ganoderma lucidum*	Basidiomycota	Gansp1_117607	202	4.73E-22	JGI
*Heterobasidion annosum*	Basidiomycota	Hetan2_436865	201	8.66E-21	JGI
*Heterobasidion irregulare*	Basidiomycota	-	201	2E-06	NCBI
*Malassezia globosa*	Basidiomycota	MGL_3627	200	2.00E-23	NCBI
*Microbotryum violaceum*	Basidiomycota	-	188	7E-13	NCBI
*Omphalotus olearius*	Basidiomycota	-	196	2E-04	NCBI
*Phanerochaete carnosa*	Basidiomycota	Phaca1_160579	199	5.31E-18	JGI
*Phanerochaete chrysosporium*	Basidiomycota	Phchr1_3440	199	2.04E-6	JGI
*Postia placenta*	Basidiomycota	POSPLDRAFT_103847	199	3.00E-15	NCBI
*Postia placenta*	Basidiomycota	POSPLDRAFT_93097	135*	1.00E-09	NCBI
*Punctularia strigosozonata*	Basidiomycota	Punst1_52328	197	3.75E-23	JGI
*Rhodotorula glutinis*	Basidiomycota	RTG_01234	197	9.00E-29	NCBI
*Rhodotorula graminis*	Basidiomycota	Rhoba1_1_52552	197	1.00E-122	JGI
*Schizophyllum commune*	Basidiomycota	SCHCODRAFT_58862	196	1.00E-18	NCBI
*Serpula lacrymans*	Basidiomycota	SERLA73DRAFT_191001	200	2.00E-29	NCBI
*Stereum hirsutum*	Basidiomycota	Stehi1_124932	202	1.07E-25	JGI
*Trametes versicolor*	Basidiomycota	Trave1_171260	202	1.12E-22	JGI
*Ustilago maydis*	Basidiomycota	Um10481	213	4.31E-25	FungiDB
*Wallemia sebi*	Basidiomycota	Walse1_59511	186	3.68E-27	JGI
*Spizellomyces punctatus*	Chytridiomycota	SPPG_04405.2	191	1.04E-40	Broad
*Allomyces macrogynus*	Chytridiomycota	AMAG_03424.1	205	2.94E-35	Broad
*Allomyces macrogynus*	Chytridiomycota	AMAG_04742.1	205	1.62E-33	Broad
*Batrachochytrium dendrobatidis*	Chytridiomycota	BDEG_07033	185	2.49E-34	Broad
*Rhizopus oryzae*	Mucormycotina	RO3G_06344.3	191	3.49E-28	Broad
*Mucor circinelloides*	Zygomycete	Mucci2_157438	191	6.70E-24	JGI
*Phycomyces blakesleeanus*	Zygomycete	Phybl2_131210	192	1.11E-17	JGI

^+^The number of amino acids in the fungal DJ-1 proteins.

“-” indicates the accession number is not available.

*Indicates the sequence was incorrectly predicted by NCBI due to sequence gaps.

The distribution of DJ-1 proteins in the two phyla of higher fungi is different. The majority of fungal species within the largest and most phylogenetically diverse phylum of fungi, the Ascomycota, lack DJ-1. We identified 10 DJ-1 proteins from the Pezizomycotina, all of them belonging to the Dothideomycetes class. In addition, we identified DJ-1 proteins from all four sequenced *Schizosaccharomyces* species (*S. pombe*, *Schizosaccharomyces octosporus*, *Schizosaccharomyces cryophilus* and *Schizosaccharomyces japonicus*) belonging to the Taphrinomycotina, the basal subphylum of Ascomycota. These four fission yeast species are the only members of the Taphrinomycotina whose genomes have been sequenced so far. Unlike species in the Ascomycota, many sequenced species of the Basidiomycota phylum contain DJ-1 proteins. We identified 25 basidiomycete DJ-1 proteins in 39 species from all three subphyla of Basidiomycota.

Fungal species from the basal phyla Chytridiomycota, Zygomycota and Microsporidia are currently under-represented in the current genomic databases. Nevertheless, DJ-1 proteins were identified in all completely sequenced chytridiomycetes and zygomycetes. Unlike chytridiomycetes and zygomycetes, all sequenced microsporidians appear to lack DJ-1 proteins likely due to their extreme genome reduction and compaction [62].

Among some of fungal species that possess DJ-1 proteins, three species have two DJ-1 proteins. Fission yeast *S. japonicus* and chytrid species *Allomyces macrogynus* contain two candidate DJ-1 proteins with 70% and 94% sequence identities, respectively. In addition, the basidiomycete *Postia placenta* also appears to have two DJ-1 proteins (POSPLDRAFT_103847 and POSPLDRAFT_93097), although the full-length sequence of the latter candidate could not be accurately predicted due to the sequence gaps.

### Conservation of candidate fungal DJ-1 proteins

**Figure 1:**
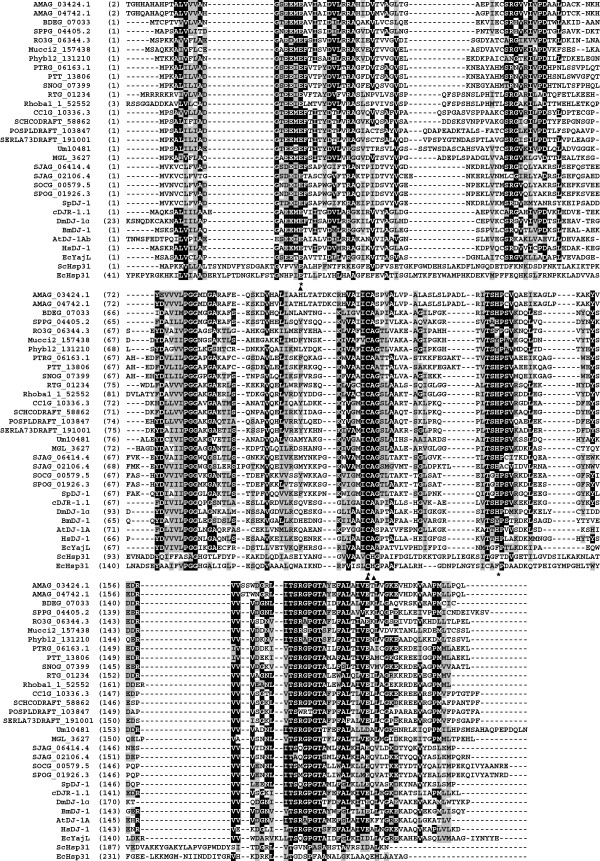
**Alignment of DJ-1 proteins from representative fungal species.** Candidate fungal DJ-1 proteins are from six ascomycetes, nine basidiomycetes and six basal fungal species. Accession numbers of fungal DJ-1 proteins are used; see Table 1 for details. DJ-1 proteins from human (HsDJ-1), *D. melanogaster* (DmDJ-1α), *C. elegans* (cDJR-1.1), *Bombyx mor*i (BmDJ-1) and *A. thaliana* (AtDJ-1A), EcYajL, EcHsp31 and ScHsp31 are included for comparison. For AtDJ-1A, only the C-terminal region is used for comparison. The alignment was constructed using Clustal W [91] and adjusted manually to improve the alignment. Identical residues are on a black ground and conserved residues shared in gray. The conserved catalytic triad residues of DJ-1 proteins and Hsp31 proteins are indicated below the alignment by stars and filled triangles, respectively. Hyphens represent gaps introduced into sequences for maximum alignment.

To evaluate the sequence conservation between fungal and higher eukaryotic DJ-1 proteins, we aligned DJ-1 sequences from fungi, human, fly, worm, and plant (Figure 1 and Additional file 1). We also included EcYajL and EcHsp31 for comparison. The fission yeast DJ-1 protein sequences were more divergent from human, fly, worm, and plant DJ-1 protein sequences than any other identified fungal DJ-1 sequences. Moreover, DJ-1 protein sequences from basal fungi are more similar to those from selected higher eukaryotes compared with their homologs from higher fungi. For example, DJ-1 proteins from basal and higher fungi share 40-43% and 23-39% overall sequence identity to HsDJ-1, respectively.

Fungal DJ-1 proteins exhibit a degree of amino acid sequence conservation with EcYajL, with identity ranging from 23-36% (Additional file 2). In contrast, fungal DJ-1 proteins exhibit extremely weak sequence similarity to EcHsp31, sharing as little as 9% amino acid identity with EcHsp31, illustrating extraordinary degree of divergence between DJ-1 and Hsp31 proteins (Additional file 2).

While the majority of fungal DJ-1 proteins have a conserved catalytic triad (Glu16-Cys111-His130 in SpDJ-1), the conserved His residue of the catalytic triad is replaced by either Phe or Tyr in three identified zygomycete DJ-1 proteins (RO3G_06344.3, Mucci2_157438 and Phybl2_131210) (Figure 1). Interestedly, this conserved His residue is also replaced by Tyr in the *Bombyx mori* DJ-1 protein (BmDJ-1) [63].

### Candidate Hsp31 proteins are widely present in fungal species

To identify Hsp31 proteins from fungi, we conducted BLAST searches against fungal databases using the protein sequence of EcHsp31 as a query. Unlike DJ-1 proteins, candidate Hsp31 proteins were found in all fungal species we examined, except 7 Saccharomycotina species, 4 Basidiomycota species and 2 Chytridiomycota species. Overall, we identified a total of 142 fungal Hsp31 proteins from 83 fungal species, including 69 Ascomycota species, 10 Basidiomycota species, 1 Chytridiomycota species, and 3 Zygomycota species (Table 2 for representative list of fungal Hsp31 proteins. For a complete list see Additional file 3).

**Table 2:** Distribution of Hsp31 proteins from representative fungi

**Species**	**Taxonomy**	**Accession number**/**name**	**Database**	**No. aa**^+^	**Class Type**	**E**-**value**
*Ajellomyces dermatitidis*	Ascomycota	BDBG_02344	NCBI	231	Class II	5.0E-19
*Arthroderma benhamiae*	Ascomycota	ARB_05407	NCBI	240	Class II	1.0E-67
*Aspergillus nidulans*	Ascomycota	AN6796.2	NCBI	237*	Class II	4.0E-15
*Aspergillus nidulans*	Ascomycota	AN6810.2	NCBI	256*	Class III	1.0E-04
*Candida albicans*	Ascomycota	CaO19.251/Glx3p	NCBI	236	Class II	5.0E-104
*Candida albicans*	Ascomycota	CaO19.7882	NCBI	236	Class II	2.0E-104
*Cordyceps militaris*	Ascomycota	CCM_05507	NCBI	296	Class I	5.0E-03
*Cordyceps militaris*	Ascomycota	CCM_09248	NCBI	225	Class II	1.0E-23
*Metarhizium acridum*	Ascomycota	MAC_07323	NCBI	228	Class II	9.0E-25
*Metarhizium acridum*	Ascomycota	MAC_05717	NCBI	294	Class I	1.0E-03
*Neurospora crassa*	Ascomycota	NCU06603	NCBI	242	Class II	8.0E-17
*Phaeosphaeria nodorum*	Ascomycota	SNOG_00505	NCBI	262	Class III	1.0E-08
*Phaeosphaeria nodorum*	Ascomycota	SNOG_04306	NCBI	229	Class II	6.0E-17
*Pyrenophora teres f. teres*	Ascomycota	PTT_13641	NCBI	229	Class II	2.0E-19
*Pyrenophora teres f. teres*	Ascomycota	PTT_19431	NCBI	252	Class III	1.0E-08
*Saccharomyces cerevisiae*	Ascomycota	YDR533C	NCBI	237	Class II	1.0E-171
*Saccharomyces cerevisiae*	Ascomycota	YPL280W	NCBI	237	Class II	2.0E-117
*Saccharomyces cerevisiae*	Ascomycota	YOR391C	NCBI	237	Class II	2.0E-04
*Saccharomyces cerevisiae*	Ascomycota	YMR322C	NCBI	237	Class II	2.0E-116
*Schizosaccharomyces pombe*	Ascomycota	SPCC757.03c/Hsp3101	NCBI	244	Class II	8.0E-63
*Schizosaccharomyces pombe*	Ascomycota	SPAC5H10.02c/Hsp3102	NCBI	240	Class II	1.0E-56
*Schizosaccharomyces pombe*	Ascomycota	SPBC947.09/Hsp3103	NCBI	262	Class II	1.0E-55
*Schizosaccharomyces pombe*	Ascomycota	SPAC11D3.13/Hsp3104	NCBI	222	Class II	2.0E-42
*Schizosaccharomyces pombe*	Ascomycota	SPAC1F7.06/Hsp3105	NCBI	251	Class II	5.0E-33
*Verticillium dahliae*	Ascomycota	VDAG_08958	NCBI	293	Class I	2.0E-03
*Verticillium dahliae*	Ascomycota	VDAG_09321	NCBI	235	Class II	4.0E-16
*Yarrowia lipolytica*	Ascomycota	YALI0C22000p	NCBI	239	Class II	7.0E-79
*Yarrowia lipolytica*	Ascomycota	YALI0F00682p	NCBI	250	Class II	5.0E-70
*Coprinopsis cinerea*	Basidiomycota	CC1G_10162	NCBI	270	Class III	1.0E-05
*Coprinopsis cinerea*	Basidiomycota	CC1G_11702	NCBI	231	Class II	2.0E-09
*Coprinopsis cinerea*	Basidiomycota	CC1G_00260	NCBI	230	Class II	4.0E-20
*Malassezia globosa*	Basidiomycota	MGL_4192	NCBI	240	Class II	2.0E-73
*Postia placenta*	Basidiomycota	Pospl1_110200	JGI	224	Class II	1.4E-08
*Postia placenta*	Basidiomycota	Pospl1_115118	JGI	249	Class II	3.6E-19
*Rhodotorula graminis*	Basidiomycota	Rhoba1_1_64353	JGI	232	Class II	7.0E-25
*Schizophyllum commune*	Basidiomycota	SCHCODRAFT_46162	NCBI	226	Class II	6.0E-28
*Schizophyllum commune*	Basidiomycota	SCHCODRAFT_49614	NCBI	225	Class II	2.0E-22
*Serpula lacrymans*	Basidiomycota	SERLA73DRAFT_120613	NCBI	224	Class II	6.0E-27
*Ustilago maydis*	Basidiomycota	UM00094.1	NCBI	235	Class II	2.0E-16
*Spizellomyces punctatus*	Chytridiomycota	SPPG_02734.3	BROAD	260	Class II	5.5E-11
*Spizellomyces punctatus*	Chytridiomycota	SPPG_05672.3	BROAD	231	Class II	3.0E-18
*Rhizopus oryzae*	Mucormycotina	RO3G_07202.3	BROAD	240	Class II	3.0E-65
*Mucor circinellodes*	Zygomycota	Mucci2_157529	JGI	240	Class II	4.3E-70
*Phycomyces blakesleeanus*	Zygomycota	Phybl2_109595	JGI	243	Class II	2.7E-72

^+^The number of amino acids in the fungal Hsp31 proteins.

The number of Hsp31 proteins is highly variable among fungal species (Additional file 4). It is notable that while the majority of fungal species have one or two candidate Hsp31 proteins, some fungal species have as many as four Hsp31 proteins. The ascomycetous fungus *Nectria haematococca* (asexual name *Fusarium solani*), appears to possess the largest number of Hsp31 proteins (7) so far identified. Another ascomycete, *Aspergillus niger*, has the second largest number of Hsp31 proteins (5). *S. cerevisiae* has four Hsp31 homologs, YDR533C, YPL280W, YOR391C and YMR322C, which have been named ScHsp31, ScHsp32, ScHsp33 and ScHsp34 (also called Sno4), respectively [60]. *S. pombe* has five Hsp31 proteins, which we named Hsp3101 (SPCC757.03c), Hsp3102 (SPAC5H10.02c), Hsp3103 (SPBC947.09), Hsp3104 (SPAC11D3.13) and Hsp3105 (SPAC1F7.06).

### Conservation of candidate fungal Hsp31 proteins

**Figure 2:**
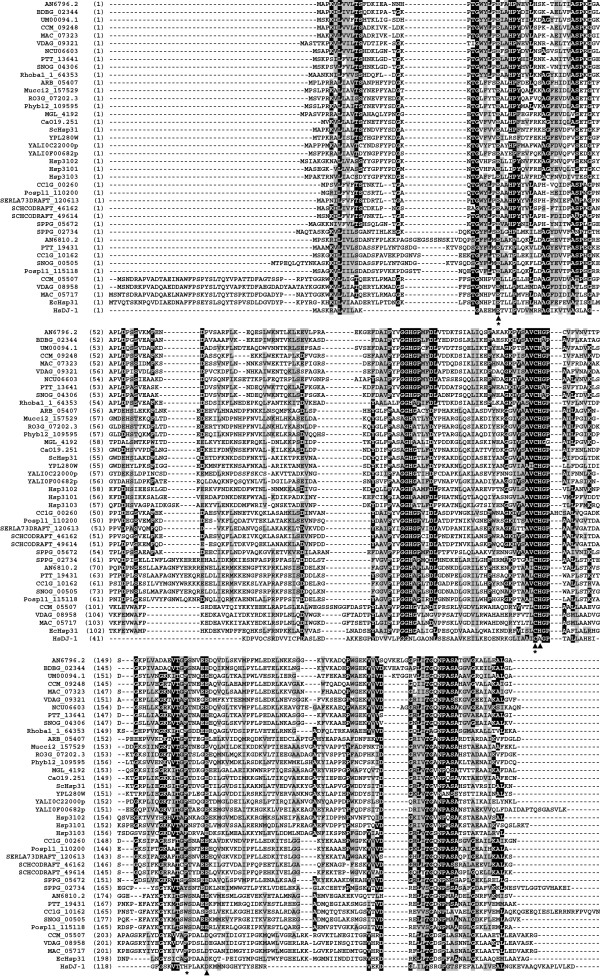
**Multiple sequence alignment of fungal Hsp31 proteins.** Candidate fungal Hsp31 proteins are from thirteen ascomycetes, seven basidiomycetes and four basal fungal species. EcHsp31 and HsDJ-1 are included for comparison. See Table 2 for more information. The annotation of the alignment is as described in the legend to Figure 1.

To assess the extent of sequence conservation among fungal Hsp31 proteins, we performed multiple sequence alignment of Hsp31 protein sequences from taxonomically diverse fungal species (Figure 2; for a complete list see Additional file 5). For comparison, we also included HsDJ-1 and EcHsp31.

Based on sequence comparison, fungal Hsp31 proteins can be divided into different classes. The majority of fungal Hsp31 proteins belong to class II that contains a short P domain. In contrast, only five proteins belong to class I that contains a P domain. Interestingly, class I fungal Hsp31 proteins were only found in the Pezizomycotina species that belong to the order Hypocreales within the class Sordariomycetes. In addition to these previously noted classes, some fungal Hsp31 proteins appear to form a new class which we have termed class III. Class III Hsp31 proteins have a short P domain and are highly divergent in sequence from other classes of Hsp31 proteins. These proteins were found mainly in Pezizomycotina species, but were absent from all examined Saccharomycotina species.

The sequence similarity between different Hsp31 proteins of the same species varies considerably among different species. In *S. cerevisiae*, the four Hsp31 proteins, which all belong to class II, are highly similar to each other (84% average identity). Among these, ScHsp32, ScHsp33 and ScHsp34 are almost identical to each other (99.5% average identity). Similarly, two *Candida albicans* Hsp31 proteins, all belonging to class II, share 99.6% identity. In contrast, the two *Aspergillus nidulans* Hsp31 proteins belonging to class II and class III, respectively, share only 19% identity. Among the five *S. pombe* Hsp31 proteins, which all belong to class II, Hsp3101 and Hsp3102 are more closely related to each other than they are to other Hsp31 proteins (58% identity between Hsp3101 and Hsp3102).

### The phylogenetic relationship between fungal DJ-1 and Hsp31 proteins

**Figure 3:**
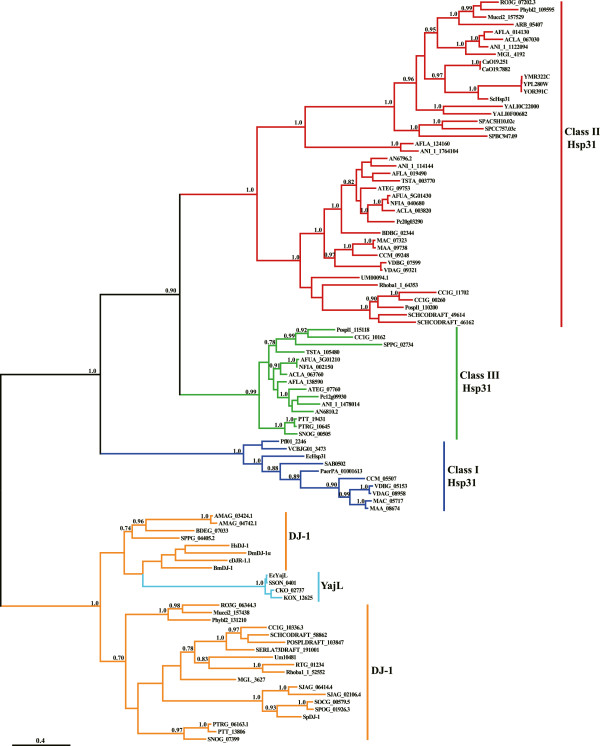
**Phylogenetic tree of candidate DJ**-**1 and Hsp31 proteins identified in fungi.** The phylogenetic tree was constructed based on multiple sequence alignments of fungal DJ-1 and Hsp31 proteins whose accession numbers are shown in Table 1 and Additional file 3. Bacterial Hsp31 proteins from *E. coli* (EcHsp31), *Pseudomonas aeruginosa* (PaerPA_01001613), *Pseudomonas fluorescens* (Pfl01_2246) *Vibrio cholerae* (VCBJG01_3473), and *Staphylococcus aureus* (SAB0502), and bacterial YajL proteins from *E. coli* (EcYajL), *Citrobacter koseri* (CKO_02737), *Klebsiella oxytoca* (KOX_12625), and *Shigella sonnei* (SSON_0401) were also included in the analysis. Bayesian posterior probabilities generated by using Bayesian MCMC sampling are indicated at the nodes. The scale bar indicates 0.4 nucleotide substitutions per site.

To examine the relationship between fungal DJ-1 and Hsp31 proteins, we generated a Bayesian phylogenetic tree from an alignment of representative fungal DJ-1 and Hsp31 proteins. In addition to fungal sequences, we also included Hsp31 and YajL proteins from representative bacterial species and DJ-1 proteins from representative metazoan species in our analyses. Our phylogenic reconstruction revealed that bacterial YajL proteins and fungal and metazoan DJ-1 proteins form a monophyletic group to the exclusion of all Hsp31 proteins, indicating that DJ-1 and YajL proteins are very closely related to each other. By contrast, DJ-1 and Hsp31 proteins form two sister clades with Bayesian posterior probability of 1.00 (Figure 3). The phylogenic reconstitution also revealed that fungal Hsp31 proteins can be further divided into different classes. This is congruent with the classification of fungal Hsp31 proteins based on sequence analysis. In addition, the phylogeny suggests that the presence of multiple *hsp31* genes identified in many fungal species is due to recent duplication events.

### Prediction of subcellular localization of candidate fungal DJ-1 and Hsp31 proteins

We predicted the subcellular localization of DJ-1 and Hsp31 proteins using the programs Mitoprot, TargetP and iPSORT. Of all 46 fungal DJ-1 proteins analyzed, only DJ-1 from the basidiomycetous anamorphic yeast *Rhodotorula glutinis* (RglDJ-1, accession number: RTG_01234) has potential nuclear localization signals (NLSs). RglDJ-1 appears to have two NLS: ^2^RRRR^6^ and ^184^RKKR^187^, which reside at the N- and C-terminus, respectively. However, despite a lack of NLSs in fungal DJ-1 proteins, it is possible that they are localized in nucleus given that a classical NLS could not be detected in HsDJ-1, but HsDJ-1 is localized in the nucleus [64-66], as well as the cytoplasm and mitochondria [22,67,68]. Indeed, we found that SpDJ-1 localizes to both the nucleus and cytoplasm (see below). Among 142 fungal Hsp31 proteins identified, 8 Hsp31 proteins are predicted to the nucleus, and 11 Hsp31 proteins are predicted to be targeted to the mitochondria (Additional file 6).

### Hsp3101, Hsp3102, ScHsp31 and SpDJ-1 possess GSH-independent glyoxalase activity

**Figure 4:**
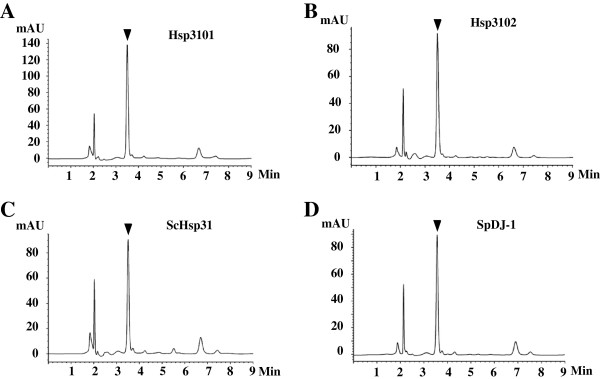
**HPLC analysis of products formed by Hsp3101 (A), Hsp3102 (B) and ScHsp31, (C) SpDJ-1 (D).** Purified recombinant Hsp3101 (257 μg), Hsp3102 (240 μg), ScHsp31 (114 μg) or SpDJ-1 (450 μg) was mixed with 10 mM MG and incubated for 30 min. Aliquots of the reaction mixtures were analyzed using a Ultimate AQ-C18 column (4.6 × 150 mm) eluted at a flow rate of 1.0 ml/min with 10 mM H_3_PO_4_ (pH 2.5). The lactate peak marked by an inverted triangle was identified by comparison with the retention time of commercial lactate.

Candidate Hsp31 and DJ-1 proteins are present in fungi, but their roles in MG detoxification have not been studied in any fungal species. To determine whether fungal Hsp31 and DJ-1 proteins are indeed GLO3 enzymes, we chose to examine DJ-1 and Hsp31 proteins from *S. pombe* and *S. cerevisiae*, including SpDJ-1, Hsp3101-3104, ScHsp31 and ScHsp32. Hsp3105 was not examined because it lacks the conserved residues critical for GLO3 activity and is unlikely to possess GLO3 activity (Additional file 7). We also did not examine the GLO3 activity of ScHsp33 and ScHsp34 because their sequences are almost identical to the sequence of ScHsp32. We were able to obtain all but two of the soluble recombinant proteins by using either the pET expression system or the ESP^®^ yeast protein expression. Two exceptions were Hsp3103 and Hsp3104, which were expressed as inclusion bodies in *E. coli* and *S. pombe*. As expected, SpDJ-1, Hsp3101, Hsp3102 and ScHsp31 could directly convert MG to lactate, which was detected by using the DNPH colorimetric method. The HPLC analysis of the reaction mixtures showed a peak with a retention time of 3.5 min identical to that expected for lactate, confirming the conversion of MG to lactate by these recombinant proteins (Figure 4). In contrast, recombinant ScHsp32 exhibited no detectable GLO3 activity.

We measured the *K*_m_ and *k*_cat_ values for the recombinant proteins that exhibited glyoxalase activity. Hsp3101, Hsp3102 and ScHsp31 have comparable apparent *k*_cat_/*K*_m_ values for MG (Table 3). These values are similar to those reported for HsDJ-1 [6] and EcHsp31 [7]. In contrast, the *k*_cat_/*K*_m_ value for SpDJ-1 is 7.9 × 10^3^ min^-1^ M^-1^, which is about 15-fold lower than those for HsDJ-1 and EcHsp31 (Table 3). The reduction in catalytic efficiency for MG apparently is primarily caused by an increase in the *K*_m_ value.

**Table 3:** **The enzyme kinetic parameters of SpDJ**-**1**, **the predicted catalytically inactive mutants of SpDJ**-**1**, **Hsp3101**, **Hsp3102**, **ScHsp31 and ScHsp32**

**Enzyme**	***K***_**m **_**(mM)**	***k***_**cat **_**(min**^**-** **1**^**)**	***k***_**cat**_**/*K***_**m **_**(min**^**-1**^ **M**^**-1**^**)**
SpDJ-1	10.8	85.7	7.9 × 10^3^
SpDJ-1E16A	ND	ND	ND
SpDJ-1C111A	ND	ND	ND
SpDJ-1H130A	37.3	65.5	1.8 × 10^3^
Hsp3101	1.4	31.1	2.3 × 10^4^
Hsp3102	2.7	58.0	2.1 × 10^4^
ScHsp31	1.5	75.0	5.1 × 10^4^
ScHsp32	ND	ND	ND

All enzyme assays were done in triplicate, and the mean values are presented. ND, not determined. The kinetic parameters of SpDJ-1E16A, SpDJ-1C111A and ScHsp32 cannot be accurately measured because of the extremely low activity of these proteins.

Fungal DJ-1 and Hsp31 proteins have similar predicted catalytic triads (Glu16-Cys111-His130 in SpDJ-1 and Glu30-Cys138-His139 in EcHsp31). To test whether the proposed catalytic triad is important for the GLO3 activity of SpDJ-1, we individually mutated these residues to Ala and purified the mutant enzymes (SpDJ-1E16A, SpDJ-1C111A and SpDJ-1H130A). Mutations of Glu16 and Cys111 to Ala nearly completely abolished the enzymatic activity, whereas the H130A substitution led to a 5- to 6-fold reduction in the catalytic efficiency.

### Overexpression of *hsp3101*, *hsp3102*, *ScHSP31* and *EcHsp31* can confer resistance to MG and GO

Since Hsp3101, Hsp3102 and SpDJ-1 possess GLO3 activity *in vitro*, we wanted to assess whether Hsp3101-3103 and SpDJ-1 can function as GLO3 *in vivo*. We first deleted their corresponding genes individually or in combination and examined the sensitivity of the mutant strains to MG and GO. These mutant strains showed sensitivity similar to that of the isogenic wild-type strain, suggesting that Hsp3101-3103 and SpDJ-1 may be functionally redundant for MG and GO detoxification (Additional file 8 and data not shown).

**Figure 5:**
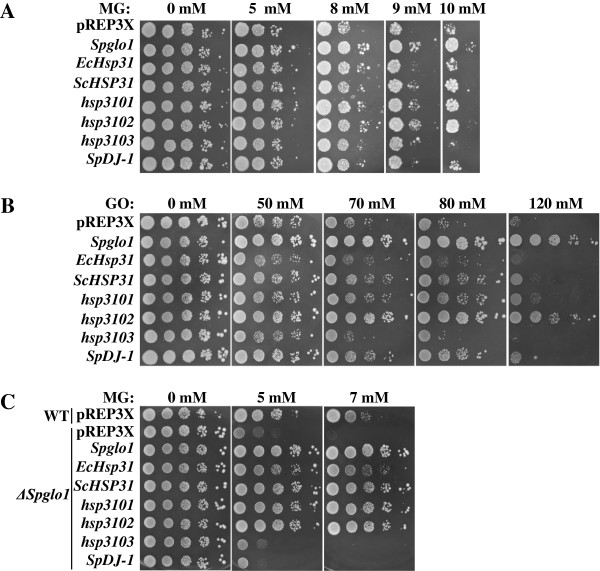
**Overexpression of *hsp3101***, ***3102***, ***ScHSP31 *and *EcHsp31 *increases resistance of wild**-**type and Δ*Spglo1 *cells to α**-**dicarbonyls. (A**-**B)** Wild-type cells harboring pREP3X (empty plasmid) or indicated constructs overexpressing *Spglo1* (positive control), *EcHsp31*, *ScHSP31*, *hsp3101*-*3103* and *SpDJ*-*1*. **(C)** Wild-type cells harboring pREP3X and Δ*Spglo1* cells harboring indicated constructs. Cells were grown to stationary phase in leucine-selective medium. Equivalent numbers of cells, in 10-fold serial dilutions (from left to right), were spotted on leucine-selective media without or with the indicated concentration of either MG **(A** and **C)** or GO **(B)** and were incubated at 30°C.

Next, we wanted to determine whether overexpression of *hsp3101*-*3103* and *SpDJ*-*1* under the control of the *nmt1* promoter [69] could modulate the sensitivity of *S. pombe* cells to MG and GO. Overexpression of *hsp3102* and, to a lesser extent, *hsp3102* increased the survival of wild-type *pombe* cells during growth in the presence of either MG or GO. In fact, overexpression of *hsp3102* displayed increased MG and GO resistance to a degree similar to overexpression of *Spglo1* (*S. pombe GLO1*). Interestingly, overexpression of *ScHSP31* and, to a lesser extent, *EcHsp31* in wild-type *pombe* cells increased resistance to MG or GO, suggesting functional conservation of Hsp31 proteins across species. Unlike overexpression of *hsp3101*-*3103*, overexpression of *SpDJ*-*1* led to enhanced resistance to GO, but not to MG (Figure 5A and B). In contrast, overexpression of *hsp3103*, *ScHSP32* and *HsDJ*-*1* did not affect the sensitivity of wild-type *S. pombe* cells to MG or GO (Figure 5A and B and Data not shown).

We also tested the effect of overexpression of *hsp3101*-*3103* and *SpDJ*-*1* in a ∆*Spglo1* strain on sensitivity to MG. The ∆*Spglo1* strain is much more sensitive to MG than the wild-type strain, which is consistent with its role as the major enzyme responsible for MG detoxification (Figure 5A and [42]). Similar to overexpression of *Spglo1*, overexpression of *ScHSP31*, *EcHsp31*, *hsp3101* and *hsp3102* markedly suppressed the MG sensitivity of ∆*Spglo1* cells, indicating that overexpression of these *hsp31* genes were able to compensate for the absence of *Spglo1* in *S. pombe* (Figure 5C). In contrast, overexpression of *hsp3103*, *ScHSP32*, *SpDJ*-*1* and *HsDJ*-*1* did not affect the sensitivity of the ∆*Spglo1* mutant to MG (Figure 5C and Data not shown).

### Hsp3101 and SpDJ-1 are both nuclear and cytoplasmic whereas Hsp3102 is exclusively cytoplasmic

**Figure 6:**
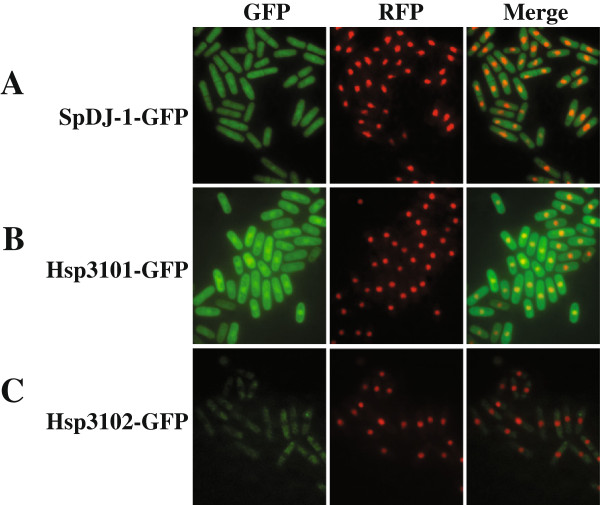
**The subcellular localization of the endogenous *S. pombe *DJ-1 and Hsp31 proteins.** Cells expressing different GFP tagged fusion proteins were grown to stationary phase in YES. The subcellular localization of GFP fusion proteins was examined and photographed by fluorescence microscopy. Left panels show the fluorescent signals for GFP-tagged SpDJ-1 **(A)**, Hsp3101-GFP **(B)** and Hsp3102-GFP **(C)**, middle panels show nuclear localization of a known nuclear protein, tRNA 3′ endonuclease tRNase Z, SpTrz1, tagged with red fluorescent protein (RFP), and right panels show overlaid images of GFP and RFP signals.

A previous genome-wide subcellular localization study using enhanced green fluorescent protein (GFP) fusions under the control of the intermediate-strength nmt1 promoter [69] showed that SpDJ-1, Hsp3101, and Hsp3102 are present in both the nucleus and cytoplasm, Hsp3104 is cytoplasmic, and Hsp3105 is predominantly nuclear [70]. However, the subcellular localization of Hsp3103 is unclear. Since the GFP fusions used in the genome-wide study may be mislocalized due to overexpression of these fusions [69]. To determine the subcellular localization of Hsp3103 and to verify the results from the genome-wide study, we examined the localization of the C-terminal GFP fusions of SpDJ-1 and Hsp3101-3105 expressed from their endogenous loci. Consistent with results obtained by the genome-wide study, SpDJ-1-GFP and Hsp3101-GFP are localized in the nucleus and cytoplasm (Figure 6A and B). However, in our analysis, Hsp3102-GFP is only localized in the cytoplasm, not in nucleus (Figure 6C). The localization of other GFP-tagged Hsp31 proteins could not be unequivocally determined, most likely due to very low levels of expression of these proteins.

## Discussion

To better understand the origin and evolution of GLO3, we performed the first comprehensive survey of DJ-1 and Hsp31 proteins in a wide range of taxonomically diverse fungal species. Our studies revealed that most fungal species examined lack DJ-1 proteins. The fact that DJ-1 proteins are present in many basidiomycetes and all sequenced basal fungi, but are absent from most species in the Ascomycota, suggests that DJ-1 proteins were lost in recent fungal evolution. Unlike DJ-1 proteins, Hsp31 proteins are widely distributed among phylogenetically distant fungal species, indicating that Hsp31 proteins were retained during fungal evolution. The distribution of DJ-1 and Hsp31 proteins in fungi appears to be in sharp contrast to the situation in metazoans, where DJ-1 proteins are ubiquitous, whereas Hsp31 proteins are largely absent (Z. Wang and Y. Huang, unpublished results). These results imply that DJ-1 proteins appeared before the divergence of metazoans from fungi, and that Hsp31 proteins were lost after the divergence of metazoan and fungal lineages.

To determine whether fungal DJ-1 and Hsp31 proteins are functional GLO3, we characterized candidate DJ-1 and Hsp31 proteins from *S. pombe* and *S. cerevisiae*. It appears that SpDJ-1, Hsp3101, Hsp3102 and ScHsp31 possess GLO3 activity *in vitro*. However, under our assay conditions, SpDJ-1 seems to have a lower enzymatic activity than other GLO3 enzymes, suggesting that SpDJ-1 may be less efficient as a GLO3 in *S. pombe*.

Although direct evidence demonstrating the *in vivo* role of fungal Hsp31 proteins in MG and GO detoxification is still lacking, we showed that overexpression of the two most closely related *S. pombe hsp31* genes (*hsp3101* and *hsp3102*) could confer MG and GO resistance on *S. pombe* wild-type and ∆*Spglo1* cells, suggesting that these two *S. pombe* Hsp31 proteins may play some role in protecting cells from MG and GO toxicity. We also found that overexpression of *ScHSP31* and *EcHsp31* could also increase the resistance of *S. pombe* cells to MG and GO, suggesting functional conservation of Hsp31 proteins from *E. coli*, *S. cerevisiae* and *S. pombe*. It will be important in future studies to elucidate the physiological function of GLO3 enzymes.

It is not clear at present why many fungal species bear more than one Hsp31 genes. Some of them do not appear to encode GLO3. Among five *S. pombe* Hsp31 proteins, Hsp3105 lacks the conserved residues critical for GLO3 activity, and is thus unlikely to function as a GLO3. Our biochemical studies reveal that *S. cerevisiae* ScHsp32 did not exhibit any GLO3 activity. Since the sequence of ScHsp32 is nearly identical to those of ScHsp33 and ScHsp34, it is likely that only one of the four *S. cerevisiae* Hsp31 proteins (ScHsp31) has GLO3 activity. Other Hsp31 proteins (Hsp32, 33, and 34) may have other *in vivo* substrates. In support of this idea, it has recently found that the size and orientation of the active site pockets of the Hsp31 and Hsp33 are markedly different [71].

Unlike Hsp3102, Hsp3101 and SpDJ-1 are both nuclear and cytoplasmic. Although the physiological relevance of the differences in subcellular distribution of these proteins is unclear at present, it seems likely that nuclear SpDJ-1 and Hsp3101 pools may play a role in the regulation of gene expression. This hypothesis is consistent with previous studies reporting that a nuclear pool of HsDJ-1 regulates the expression of some genes through stabilization of the antioxidant transcriptional master regulator Nrf2 [10]. For example, HsDJ-1 up-regulates thioredoxin 1 gene expression via Nrf2-mediated transcriptional induction [72].

Our analysis reveals that fungal DJ-1 proteins are phylogenetically most closely related to bacterial YajL proteins, consistent with previous studies [40,41,43]. Besides, the overall structures of DJ-1 and YajL are also very similar (e.g., Cα RMSD between HsDJ-1 and EcYajL is 0.9 Å) [73]. It is thus likely that DJ-1 and YajL proteins may have evolved from the same ancestor protein. In contrast, Hsp31 proteins are much less similar in sequence to DJ-1 proteins than are YajL proteins [73]. Moreover, although both Hsp31 and DJ-1 proteins contain the same structurally conserved core domain characterized by an *α*/*β* sandwich fold, Hsp31 proteins have structural properties, including an additional P domain and a different dimerization interface, that distinguish them from DJ-1 proteins [59,60]. However, despite the extensive sequence and structural divergence [59,60,74-78], many identified *E. coli* and fungal Hsp31 proteins possess GLO3 activity *in vitro*, suggesting that DJ-1 and Hsp31 proteins may have emerged independently.

During preparation of this manuscript, Hasim *et al*. reported the crystal structure and the functional studies of the *Candida albicans* Hsp31 protein, Glx3p (Systematic name: CaO19.251) [79]. They demonstrated that Glx3p possesses *in vitro* GLO3 activity. The *k*_cat_/*K*_m_ value of Glx3p reported by Hasim *et al.* is 1.4 × 10^3^ M^-1^S^-1^, which is comparable to our *k*_cat_/*K*_m_ values for Hsp3101, Hsp3102 and ScHsp31. While in this paper, Hasim *et al*. mainly focused on the *C. albicans* Glx3p, they also reported the specific activities of SpDJ-1 and ScHsp31. However, the kinetic parameters for these two enzymes were not reported. Consistent with our results, they found that Glx3p and ScHsp31 are considerably more active than SpDJ-1 (Table 3 in [79]). However, in contrast to our findings that single and combined deletions of *S. pombe DJ*-*1* and *hsp31* genes did not result in an increase in sensitivity to MG, they found that deleting *glx3* caused increased sensitivity to MG. We believe that the difference may be due to functional redundancy of *S. pombe* DJ-1 and Hsp31 proteins. Furthermore, our results extend their findings to show that some of fungal Hsp31 proteins may not be GLO3.

## Conclusions

This study represents the first large-scale identification and analysis of fungal DJ-1 and Hsp31 proteins. Our survey shows that Hsp31 proteins are widespread throughout the fungal kingdom, whereas DJ-1 proteins are restricted to certain fungal phyla. The apparent lack of DJ-1 genes in the majority of fungal species suggests that they may have lost during fungal evolution. Sequence alignment and phylogenetic analyses reveal that fungal Hsp31 proteins can be further divided into different classes. Our analysis reveals that fungal Hsp31 proteins may serve as the major GLO3 in fungi, although some of them may not be GLO3. The GLO3 activity of Hsp31 proteins may have occurred independently from that of DJ-1 proteins. Our results further suggest that Hsp3101 and Hsp3102 are the major GLO3 that may have some role in protecting cells from RCS toxicity in *S. pombe*, and that SpDJ-1 and Hsp3101 may also participate in nuclear-related functions.

## Methods

### Strains, media and genetic manipulation

All *S. pombe* strains used in this study are listed in Additional file 9. Deletion mutants were created by the one-step gene replacement method [80]. The deletion cassettes to generate the *Spglo1* and *SpDJ*-*1* null alleles were generated by cloning the 5′ and 3′ flanks of *Spglo1* and *SpDJ*-*1* into pAF1 [81] and pFA6a-kanMX6 [82], respectively. The deletion cassettes were transformed into yHL6381. Primers used for deletion cassette construction and other plasmid constructions are available upon request. All deletions were verified by PCR.

### Fungal genome database search and protein sequence analysis

Fungal DJ-1 and Hsp31 proteins were identified by BLAST searches using known DJ-1 and Hsp31 proteins as queries, respectively, and the cut-off E-value of 0.01. Fungal genome databases used include the National Center for Biotechnology Information (NCBI; http://blast.ncbi.nlm.nih.gov/Blast.cgi?PAGE_TYPE=BlastSearch&BLAST_SPEC=MicrobialGenomes), the Broad Institute (http://www.broadinstitute.org/science/data), the Joint Genome Institute (http://genome.jgi-psf.org/pages/fungi/home.jsf), the Genome Center at Washington University (http://genome.wustl.edu/), FungiDB (http://fungidb.org/fungidb), and the Universal Protein Resource (http://www.uniprot.org). All sequences were subjected to manual curation and further analysis as described [83-85].

### Phylogenetic analysis

The phylogenetic tree of candidate fungal DJ-1 and Hsp31 proteins was constructed using methods as described [83-85]. Briefly, fungal DJ-1 and Hsp31 protein sequences were aligned using the Clustal W program implemented in MEGA5.0 [86] and adjusted manually to conform to the optimized alignment of amino acid sequences. Model selection was performed using ProtTest 2.4 [87]. The Bayesian phylogenetic tree was inferred by using MrBayes version 3.1.2 [88] and a mixture of the fixed amino acid models and I + G distribution. Statistical confidence was assessed by using Markov Chain Monte Carlo (MCMC) sampling approaches. Two separate runs including a total of four independent tree searches were conducted. All searches consisted of one ‘cold’ and three ‘heated’ Markov chains estimated for 10^7^ generations, and every 100 generations were sampled. The burn-in parameter was estimated by plotting -ln*L* against the generation number using Tracer (v1.4.1, http://beast.bio.ed.ac.uk/software/Tracer), and the retained trees were used to estimate the consensus tree and the Bayesian posterior probabilities.

### Plasmid construction

To make recombinant proteins with an addition of a 6-histidine tag at their carboxyl-terminal end, *ScHSP31*-*32*, *hsp3101*-*3104* and *SpDJ*-*1* were PCR-amplified from genomic DNA, cloned into the NcoI and XhoI sites (for *ScHSP31*, *hsp3101*-*3104* and *SpDJ*-*1*) of pET28a (Novagen) or the NdeI and XhoI (for *ScHSP32*) of pET23a (Novagen). *hsp3102*-*3104*, which formed inclusion bodies when expressed in *E. coli*, were also cloned into the NdeI and BamHI sites of the yeast expression vector pESP3 (Stratagene), and were overexpressed in *S. pombe* as C-terminally His-tagged fusion proteins. The point mutations of *SpDJ*-*1* (*SpDJ*-*1E16A*, *SpDJ*-*1C111A* and *SpDJ*-*1H130A*) were generated by overlapping PCR with two sets of PCR primers including overlapping primer pairs containing the desired mutations. For expression in *S. pombe*, the coding regions of *Spglo1*, *DJ*-*1* and *Hsp31* genes were PCR-amplified from genomic DNA (for *Spglo1*, *hchA*, *ScHSP31*-*32*, *hsp3101*-*3103* and *SpDJ*-*1*) or plasmid p3xFlag-myc-CMV-24-DJ-1 [89] (for *HsDJ*-*1*). The PCR fragments were digested with BamHI and SmaI (for *DJ*-*1* and *hsp31* genes ) or with XhoI and SmaI (for *Spglo1*), and cloned into the BamHI and SmaI or the XhoI and SmaI sites of pREP3X [69].

### Green fluorescent protein (GFP) tagging of *S. pombe* DJ-1 and Hsp31 proteins

*S. pombe* DJ-1 and Hsp31 proteins were C-terminally tagged with GFP (S65T) at their respective chromosomal loci by homologous recombination. To place a GFP tag at the C terminus of spDJ-1, Hsp3101 and Hsp3102, the GFP tagging cassette containing the coding region before the stop codon, coding regions for GFP and the kanamycin resistance marker (KanMX6), and the 3′ untranslated region after the stop codon was generated by fusion PCR using pFA6a-GFP (S65T)-kanMX6 as template. To place a GFP tag at the C terminus of Hsp3103-3105, the 5′ coding region before the stop codon and the 3′ untranslated region after the stop codon were subcloned into the SalI and BamHI sites, and the SpeI and SacII sites of pFA6a-GFP (S65T)-kanMX6, respectively. The GFP tagging cassette was then generated by PCR. The GFP tagging cassettes were gel-purified and used to transform yHL6381 to G418 resistance by lithium acetate-mediated transformation. Correct integration of the GFP tag into the desired locus was verified by PCR. GFP fusion proteins were visualized by fluorescence microscopy as described [90].

### Expression and purification of recombinant proteins

Recombinant plasmids carrying wild-type *SpDJ*-*1*, *hsp3101* or *ScHSP31*, or the predicted catalytically inactive mutants of *SpDJ*-*1* (pET28a-SpDJ-1E16A, pET28a-SpDJ-1C111A and pET28a-SpDJ-1H130A) were transformed into *E. coli* BL21 (DE3) cells. The recombinant His-tagged proteins were induced with 1 mM isopropyl-*β*-D-thiogalactoside (IPTG) for 4 hrs. Cells were harvested and lysed by sonification in buffer containing 20 mM sodium phosphate, pH7.5, 14.3 mM *β*-mercaptoethanol and 0.5 mM Phenylmethylsulfonyl fluoride (PMSF), and further clarified with centrifugation. The supernatant was incubated with Ni-NTA resin (Qiagen) with rocking at 4°C. The resin was washed using buffer containing 20 mM sodium phosphate, pH7.5, and 25 mM imidazole. The proteins were eluted with 20 mM sodium phosphate buffer (pH7.5) containing 250 mM imidazole. Purified proteins were dialyzed against buffer containing 100 mM sodium phosphate, pH6.0, and 1 mM DTT, and quantitated using the Bradford assay. Recombinant Hsp3102, which formed inclusion bodies when expressed in *E. coli*, was produced with the ESP^®^ yeast protein expression system in *S. pombe* (Stratagene). The plasmid pESP3 expressing *hsp3102* (pESP3-Hsp3102) was transformed into *S. pombe* strain yAS56, and the recombinant protein was produced according to manufacturer’s instructions, except that the recombinant protein was affinity purified with Ni-NTA resin. The purified recombinant proteins were used in the *in vitro* assays and for antiserum production.

### GLO3 activity assay

For the determination of GLO3 activity *in vitro*, a colorimetric assay was used as described in references [6,7] with modifications. The reaction mixtures (200 μl) contain 100 mM Na_3_PO_4_, pH 6.8, different concentrations of MG (0.5, 1, 1.5, 2, 2.5, and 3 mM) and purified recombinant wild-type or mutant SpDJ-1 proteins, or ScHsp31 were incubated for predetermined time periods at 45°C. The reactions were stopped by the addition of 700 μl 100 mM Na_3_PO_4_, pH 6.8 and 300 μl of 2,4-dinitrophenylhydrazine (DNPH). After incubation for 15 min at room temperature, 10 ml of 10% NaOH was added. The mixtures were further incubated for 15 min, and the formation of phenylhydrazone was determined by measuring absorbance at 540 nm. Steady-state kinetic parameters were determined from initial rates, which were calculated from the changes in phenylhydrazone absorbance at 540 nm. The *k*_cat_ and *K*_m_ were calculated using OriginPro 8 software (OriginLab Corporation).

### HPLC analysis

The reactions catalyzed by SpDJ-1, Hsp3101, Hsp3102 and ScHsp31 were monitored by HPLC according a previously published protocol with modifications [6]. Briefly, HPLC analysis was carried out using an Agilent 1260 Infinity HPLC system with the Ultimate AQ-C18 column (4.6 × 150 mm, 5 μm column, Welch Materials, Inc.) at a flow rate of 1.0 ml/min. The mobile phase was a mixture of 10 mM H_3_PO_4_ (pH 2.5). Production of lactate was detected by UV absorbance at 210 nm. The retention of D-lactate (Sigma) was around 3.5 min.

### Methylglyoxal and glyoxal sensitivity assays

*S. pombe* cells were grown at 30°C overnight, diluted and grown to stationary phase. Cells were suspended in water and normalized to an OD_600_ = 3.0. 3 μl of cells were spotted in 10-fold serial dilution onto medium containing different concentrations of MG or GO (Sigma). The plates were photographed after 7 to 10 days of incubation at 30°C.

## Abbreviations

MG: Methylglyoxal; GO: Glyoxal; GSH: Glutathione; SLG: *S*-D-lactoylglutathione; GLO1: Glyoxalase I; GLO2: Glyoxalase II; GLO3: Glyoxalase III; GATase1: Glutamine amidotransferase.

## Competing interests

The authors declare that they have no competing interests.

## Authors’ contributions

QZ performed biochemical assays. ZW did online database searches and sequence analysis. YS performed all spotting assays. CC and TW carried out localization analysis. YH conceived this study, analyzed the data and drafted the manuscript. All authors have read and approved the final version of the manuscript.

## Supplementary Material

Additional file 1 **Multiple sequence alignment of candidate fungal DJ-1 proteins.** The accession numbers for the candidates are listed in Additional file 3. The annotation of the alignment is described in the legend to Figure 1. Click here for file

Additional file 2 **Percentage amino acid identities of fungal DJ-1 proteins compared with those from *C. elegans *(cDJR-1.1), *D. melanogaster *(DmDJ-1α), *A. thaliana *(AtDJ-1A), and human (HsDJ-1), *E. coli *YajL (EcYajL), and *E. coli *Hsp31 (EcHsp31).** The first column is accession numbers (For proteins lacking a accession number, species names are used). See Table 1 for details. The pair-wise percent identity scores were generated with Clustal W [91]. For AtDJ-1A, only the C-terminal region is used for comparison. Click here for file

Additional file 3 **Distribution of candidate Hsp31 proteins identified in fungi. **^+^The number of amino acids in fungal Hsp31. *Indicates that mispredicted sequences obtained from the databases have been corrected. ^?^Indicates the sequence could not be correctly predicted due to sequence gaps. “―”, the protein sequence could not be identified by BLAST searches. “#”, the accession number is not available. Click here for file

Additional file 4 The number of Hsp31 proteins in fungal species. Click here for file

Additional file 5 **Multiple sequence alignment of candidate DJ-1 and Hsp31 proteins from representative fungal species.** The accession numbers for the candidates are listed in Table 1 and Additional file 3. The annotation of the alignment is described in the legend to Figure 1. Click here for file

Additional file 6 **Candidate fungal Hsp31 proteins predicted to localize to nucleus or mitochondria.** Predicted NLSs are shown. “+” indicates the predicted mitochondrial localization. Click here for file

Additional file 7 **Multiple sequence alignment of candidate *S. pombe *** **Hsp31 proteins.** Click here for file

Additional file 8 **Deletion of individual *SpDJ*-*1 *** **and *hsp*** **3101-3103 or in combination does not affect the MG sensitivity of cells.** Click here for file

Additional file 9 **List of *S. pombe *** **strains used in this study.** Click here for file
