# Supplementary material for: Identification of glutathione (GSH)-independent glyoxalase III from Schizosaccharomyces pombe
Source: BMC Evol Biol. 2014 Apr 23;14:86. doi: 10.1186/1471-2148-14-86 (PMC4021431; doi:10.1186/1471-2148-14-86)
Supplement: Additional file 1 — Multiple sequence alignment of candidate fungal DJ-1 proteins. The accession numbers for the candidates are listed in Additional file 3. The annotation of the alignment is described in the legend to Figure 1. [file 1471-2148-14-86-S1.doc]

**Additional file 1**

**AarDJ1 (1) ---------MPRALILVADGSEEIEFVTPYDVLTRAGFEVKSAGVSLKNKS-----------YAHLSRNIRIVPDYPELSSISVD---AHERYDVLILPGGAPGAKTFC--GDNTVLDLINEF**

**Altbr1_7229 (1) ---------MPRALLLVADGSEEIEFVTPYDVLTRAGFEVKSAGVSLENEV-----------YAHMSRNIRILPDYQKLLSVPSQT--AHDYFDILILPGGAPGAKTFC--ETDSVLELVHNF**

**CocheC4_1_31923 (1) ---------MPRALILVANGSEEIEFVTPYDVLTRAGFEVKSAGVSLQNEA-----------YAHMSRNIRIVPDYLNLNSVPLQP--AHEHFDVLILPGGGPGSKTFC--ESDAVLKLIDDF**

**Coclu2_103587 (1) ---------MPKALILVADGSEEIEFVTPYDVLTRAGFQVKSAGVALQNEA-----------FAHMSRNIRIVPDYSNLQSVPLQT--AHEDFDILILPGGGPGSKTFC--ESDAVLRLIRDF**

**Cocmi1_87889 (1) ---------MPKALILIADGSEEIEFVTPYDVLTRAGFEVKSAGISLQNEA-----------YAHMSRNIRIVPDYANLNSVPLQP--AHEHFDVLILPGGGPGSKTFC--ESDAVLKLIDGF**

**Cocsa1_181323 (1) ---------MTKALILVADGSEEIEFVTPYDVLTRAGFEVKSAGVSLQNEA-----------YAHMSRNIRIVPDYPNQNSVPLQP--AHEHFDVLVLPGGVPGSKTFC--ESDAVLKLIHDF**

**Cocvi1_89530 (1) ---------MPKALILVADGSEEIEFVTPYDVLTRAGFEVKSAGVSLQNEA-----------YAHMSRNIRIVPDYSNLNSVPLQP--AHEHFDVLILPGGGPGSKTFC--ESDAVLKLIHDF**

**PTT_13806 (1) ---------MPKALILVADGSEEIEFVTPYDVLTRAGFDVTSAGVSLKNEA-----------YAHMSRNVRIVPDHSNLSWVGFQT--AHEDFDVLILPGGAPGAKAFC--GSDEVLELISKF**

**PTRG_06163.1 (1) ---------MPKALILVADGSEEIEFVTPYDVLTRAGFDVTSAGVSLKNEA-----------YAHMSRNVRIVPDHPNLSSVPLQT--AHEDFDVLILPGGAPGAKAFC--GSDAVLELISKF**

**SNOG_07399 (1) ---------MPKALILIADGSEEIEFVTPYDVLTRAGFEVQSVGVDLKNEG-----------YAHMTRNVRIVPDHTNLTSFPHQL--AHEHYDILILPGGGPGAKTFS--TNPSVLQLIKSF**

**SJAG_02106.4 (1) ---------MVKVCLFVTGGSDEIETASCYGVFTRAKTPIDTVYVCEENKER----------LVNMLCGIRLYADRSLSEFQSAEDF--MKEYDVVIIPGGWGGSLERSIPGTKMVQEIVRGM**

**SPAC22E12.03c (1) ---------MVKVCLFVADGTDEIEFSAPWGIFKRAEIPIDSVYVG-ENKDR----------LVKMSRDVEMYANRSYKEIPSADDF--AKQYDIAIIPGGGLGAKTLS--TTPFVQQVVKEF**

**SOCG_00579.5 (1) ---------MVNVCLFVADGNDEIEFSAPWGVFTRAQIPIDSVYVG-ENPEK----------LVTMSRGVQLYAKRSLKEFKSVEEF--ASHYDVVIVPGGVKGANTLS--TDEFVKKVVSSY**

**SPOG_01926.3 (1) ---------MVNVCLFVADGNDEIEFSAPWGVFTRAQIPIDSVYVG-ENPEK----------LVTMSRGVQLYAKRSLKEFKSVEEF--ASHYDVIIIPGGMKGAKTLS--TDEFVKKLVTSY**

**SJAG_06414.4 (1) ---------MVKVCLFVADGSDEIEFSAPYGIFTRANTPIDTVYVG-DNKDR----------LVNMSRGIQLYAKRSLSEFQSTEEF--VKEYDVAIIPGGWQGSLTLS--GNKKVQEIVKEM**

**Agabi_187606 (1) ------MSLPPKALILIADGTEEMEFTITYDTLVRGGIITTSAYISTTPSLDS-------PPTAKCSRGVRIMPDIYFEPSASGFGP---DKYDLLVIPGGAKGAEIMA--RDGGVQKLVRDY**

**Aurde1_110250 (1) --------MAPSALIFIADGTEEMEFTIAYDTLVRGGVECTSALVG----------VTRIDSVAICSRGVKIIADTTLESRDVAD-----KSFDLIVVPGGAEGAKTIS--KNAKVQEMIRSQ**

**Cersu1_140432 (1) ---------MPSALILIADGTEEMEFTITYDTLVRAGVACTSAYVS--ADSDTASTTQTTPSFAKGSRGINILPDTYFSPQAHVP-----DNFDLLVIPGGAKGAETIS--KNSPVQHLVREY**

**CC1G_10336.3 (1) ---------MPSAVVLLADGTEEMEFTITYDTLVRAGVQVVSAFVPAQSPG-----ASVSPPAAKCSRGVRILPDSYLDPTECGP-----DKHDLLVIPGGAVGAATMS--ANATVQKLIQAY**

**Dicsq1_101983 (1) ---------MVSALILIADGTEEMEFTITYDTLVRAGASCTSAFVSTEERQGDSGSYEKNPPFAKGSRGINILPDTYFSPQAHTP-----DKFDLLVIPGGAKGAETIS--NSSPVQHLVRQF**

**Gansp1_117607 (1) ---------MVSALILIADGTEEMEFTITYDTLVRAGASCTSAFVSSEERQGEN-TYENNPPFAKGSRGINILPDTYFSPQFSTP-----DNYDLLVIPGGAKGAETIS--NSSPVQHLVREY**

**Hetan2_436865 (1) ---------MVSALILLADGTEEMEFTIIYDTLVRAGIICTSAFVPS-DASAADTDARISPPFAKGSRGIRIQPDTYFSLQALRV-----EKFGAIIIPGGAQGARTMA--ESPAVQRLVREF**

**HirDJ1 (1) ---------MVSALILLADGTEEMEFTIIYDTLVRAGIICTSAFVPS-DASAADTDARISPPFAKGSRGIRIQPDTYFSLQALRV-----EKFGAIIIPGGAQGARTMA--ESPAVQRLVREF**

**MGL_3627 (1) ---------MPKAIVFLAQGAEEMEFSITYDVLVRGGVDVTSVYVPGADEPLSP-----ADGLVVASRGVKLGVDTTLEALTKSGHAG---DYDAYIIPGGAGGANTLS--KDPTVLQILRDS**

**OolDJ1 (1) ---------MPRALILIADGTEEMEFTITYDTLVRAGVTCRSAFVHQGSS------DSTDPLVAVGSRGIRILPDTTLEPSQCGP-----DAFDALIVPGGATGAKTIS--ENSVVQHLVREY**

**Phaca1_160579 (1) ---------MAKACILIADGTEEMEFTITYDTLVRAGVSCASAYVP--SGADNDASFTSPP-FARGSRGINILPDMFFSPQDCTP-----DKFDLLVIPGGAKGAETIS--TSSPVQHLVRRF**

**Phchr1_3440 (1) ---------MPNACILIADGTEEMEFTITYDTLVRAGVSCASAYVS--ADNDNDATFKSPP-FARGSRGINILPDMFFSPQDCTP-----DKFDLLVIPGGAKGAETIS--TNSPVQHLVRRF**

**POSPLDRAFT_103847 (1) ---------MPSALILIADGTEEMEFTITYDTLVRAGIACTSAYVPAQDAP---EADKTALSFATCSRGLKIVPDTLFSPQAAVP-----EHYDLLVVPGGAKGAQTIA--QSAPVQHLVRRY**

**Punst1_52328 (1) ---------MTAALILIAEGTEEMEFVITYDTLVRAGVQCTSAYVAAGP-------ETTDTPHATCSRGVRIVPDAFFNDDIASDAL---SRFDAIVVPGGAKGAETIS--GNSRVQSLIKSF**

**MviDJ1 (1) -------------MILVADGSEEIEVMTIFDVLVRASLGPAIVSLSPQLSPSQ------SLPYITLSRGARMLADTKFETLKQEHKD----DFDAIIIPGGAKGADRLS--SSREVQTLIRSF**

**RTG_01234 (1) -----MRRRRKRVIVLVADGTEEIEVFTAYDVFVRASLNPVLVSVSPQFSPSN------SLPHITLSRGARILADTQFETLKEEHWD----LFDAVVVPGGAKGAERLS--KEKRVQELLWRF**

**Rhoba1_1_52552 (1) MARSSGGADDKAVLVLCADGTEEIELMTVYDVLVRASLSPVIVSVSPQFSPSH------SLPHMTLSRGAKILADTTWEHLETKQPDVLATYFDAVVVPGGAKGAERLS--TDKGVQRLVRDY**

**SCHCODRAFT_58862 (1) ---------MPSALIFIADGTEEMEFTITYDTLVRAGVKVQSAFVS------ADDSKSVNPPVAKGSRGIGILPDTYFEPGNSGP-----DKFDLLVVPGGAKGAETIS--QNPAVQELIRKY**

**SERLA73DRAFT_191001 (1) ---------MPKALILIADGTEEMEFTITYDTLVRAGVSCTSALVQDVPSP--YGTNDASPTLAVGSRGISILPDTTLVPLEAGP-----DKYDAIIIPGGAKGAETIS--KSSPVQHLVREY**

**Stehi1_124932 (1) ---------MPSALILIADGTEEMEFTIVYDTLVRADVKCVSAYVSPTSAATIPEGTEQNPPFVRCSRGVCIQPDIYFTHKATTS-----NDYDVLVIPGGAKGAETMA--GFSAVQTVVKEF**

**Trave1_171260 (1) ---------MPSALILIADGTEEMEFTITYDTLVRAGVACTSAFVSERDEGDDHRSHESNPPFVKGSRGIRIVPDIYFSPSAATP-----DKFDLLVIPGGAKGAATIS--GNASIQHLVKEY**

**Um10481 (1) ---------MPSALILIAEGTEESEFTITYDVLVRGSVTTRSVLVGSSSTWSDAS-CAHSVAYVTCSRGVKIVPDLQLADVGGGKAL----EYDCIVIPGGAKGVETIS--ANADVQALVGAM**

**Walse1_59511 (1) ---------MTSALIFITNGTEEMEFTIAYDTLVRAAIDTTSAGVDIQS-------------QAVCTRGVKITPDVLLDSLSDDQLS----SYDIVVIPGGGPGANTLQ--ANKRVQKLFQDN**

**AMAG_03424.1 (1) -MTGHHAHAHPTALVVVANGSEEMEAVIAIDVLRRAHIDVTVAGLTGAEPI-------------KCSRGVVIVPDAALDACK-------NKHYEVVVLPGGMDGARAFE--QSKDVHALIAAH**

**AMAG_04742.1 (1) -MTGHQAHAQPTALVVVAHGSEEMEAVIAIDVLRRAHIDVMVAGLTGAGPI-------------KCSRGVVIVPDAALDTCK-------NKHYDVVVLPGGMDGARAFE--KSKDVHVLIATH**

**BDEG_07033 (1) -------MTCPTVVVLVANGTEEMEAVITIDILRRAQAKVLVCSLQEEKVI-------------ECSRHVRIVPDTWLAKID-------AANIDAVIMPGGMGGAKAFS--ESKDVHQLLN--**

**SPPG_04405.2 (1) --------MAPSALVLITDGNEEMEAVISIDILRRAGVDVTVAGLNGPNTV-------------ECSRHVKIVPDTGLSAVK-------DKTFDAILLPGGMGGAEAFS--KSSTVHELLS--**

**Mucci2_157438 (1) ------MSAQKKAIVFLCEGAEEMEFTISVDVLRRAKVEVTVVGVELASDV------------AVCSRGVKIAPDVKFSES--LK----ADDYDAVIIPGGNGSAKTLS--AHEGAKKLIMD-**

**RO3G_06344.3 (1) -------MSPKKAVVFLAEGAEDMEFSISVDVLRRAKIEVTVVGVDISGDY------------ATCARGIKVVPDTKLDKIN-LN----ASEYDVAIVPGGAGSAKTLG--AHKGVHKILME-**

**Phybl2_131210 (1) --------MTKKAIVFLANGTEEMEFTISADILRRAKVDVTVVGVELEKDKP-----------AICANGTKILPDILLTDKELEG----NLSYDAGVVPGGLKGSHTCR--DNKKVQAIIKN-**

*****

**AarDJ1 (99) R----KRAKWVAAICAATTALVAAHKRDDGTRKVTTTVTSHPSVKDEVVQAG--WQYSEER-VVVDQ--NIITSQGPGTAILFALTIVEELCGKEKRGEVAGPMIVAETL------------**

**Altbr1_7229 (100) R----KAGKWVAAICAATTALVAAEEKLGGG---KTRVTSHPSVKEEVAKAG--WQYSEER-VVVDG--KVVTSRGPGTAMLFALTIVDVLCGKEKRDEVAGPMVVAETL------------**

**CocheC4_1_31923 (100) Q----QAGKWVAAICAATTALVASAKKFGKEK---KRVTSHPSVAEEIKVAG--WQYSEDR-LVVDG--KVITSRGPGTAMLFALTIVQVISGKEKRDEIAGPMVLAEKL------------**

**Coclu2_103587 (100) Q----QKGRWVATICAATTALVASAEKFESAK---ARVTSHPSVSSEIQAKG--WEYSEER-IVVDG--KVITSRGPGTAMLFALTIVELVCGREKREEIAGPMVLAEK-------------**

**Cocmi1_87889 (100) Q----QAGKWVAAICAATTALVASTKKFDKDK---KRVTSHPSVAEEIKVAG--WQYSEDR-LVVDG--KVITSRGPGTAMLFALTIVEVISGKEKRDEIAGPMVLAEKL------------**

**Cocsa1_181323 (100) Q----QAGKWVAAICAATTALVASTKKFDKEK---KRVTSHPSVAEEIKVAG--WQYSEDR-LVVDG--KVITSRGPGTAMLFALTIVEVISGEEKRDEIAGPMVLAEKL------------**

**Cocvi1_89530 (100) Q----QAGKWVAAICAATTALVASTKKFDKEK---KRVTSHPSVAEEIKVGG--WQYSEDR-LVVDG--KVITSRGPGTAMLFALTIVEVISGKEKRDEIAGPMVLAEKL------------**

**PTT_13806 (100) R----KGQKWVAAICAATTALVASAKKFEGAK---TTVTSHPSVAEEIKQAG--WEYSEDR-IVVDE--KIVTSRGPGTAMAFALTIVEAMCGKGKREEIGGPMMLAEKL------------**

**PTRG_06163.1 (100) Q----KAGKWVAAICAATTALVASTKKFEGAK---TTVTSHPSVAEEIKQAG--WEYSEDR-IVVDD--KIVTSRGPGTAILFALTIVEAICGKEKRDEIGGPMMLAEKL------------**

**SNOG_07399 (100) V----RSGKFVAAICAGTTALVAAGIEK-------KIVTSHPSVMQEIKGAG--WEYSEER-VVVDG--KVVTSRGPGTALLFSLTIVEVMVGKEKRDEVAGPMVVAATL------------**

**SJAG_02106.4 (103) YK---KPGKWVAMICAGSMGVMTSGLDPKTLE-----LTSHACVIDVLRNAG--YNWVDEP-VVVSN--NLITAQGPGTSMLFALKIAEQVLDKETYQQVYASLEMPQRN------------**

**SPAC22E12.03c (100) YK---KPNKWIGMICAGTLTAKTSGLPNKQ-------ITGHPSVRGQLEEGG--YKYLDQP-VVLEE--NLITSQGPGTAMLFGLKLLEQVASKDKYNAVYKSLSMP---------------**

**SOCG_00579.5 (100) WK---KAGKVIGMICAGTLTAKTTGLSATE-------ITGHPSVRKDLEEAG--FRYIPQP-VVVEN--NLITSMGPGTALLWALKLLEQVTTKETYDTVYQALTMPEKQIVYAANRE----**

**SPOG_01926.3 (100) WK---KSGKVIGMICAGTLAAKTTGLSATE-------ITGHPSVRKDLEESG--FRYVPQP-VVVEN--NLITSMGPGTALIWALKLMEQVATKETYDAVYEALSMPEKQIVYATNRD----**

**SJAG_06414.4 (100) YN---KPGKWVAMICAGSLTAKTSGLGVKT-------LTSHPCITKDLQEAG--YEWKNES-VVVTD--NLITSQGPGTAMLFALKIAEQVLDKDTYQKVYDSLEMP---------------**

**Agabi_187606 (106) L----SQGKYVGMICAGSLVAKTSGLPSQP-------ITSHPSIKSELEN---SFEYSEDS-VVVSG--KLVTSRGPGSTFPFALTLVELLVGSEKRKEITPPMMFPSGTPW----------**

**Aurde1_110250 (99) I----NVGRFVGMICAGSLAALEAKLPSQP-------VTSHPSVKDSLKD---SFEYREDS-VVVSG--KLVTSRGPGTAFPFALKLVELLCGEAKMREVHAPMIFPGSG------------**

**Cersu1_140432 (106) I----KNGKYVGMICAGSLAALTAGLEKQP-------LTSHPSVKSHLEK---DFIYSEQP-VVVSG--RLVTSRGPGTTFPFALTLVELLCGSAKRAEVAGPMVFPPGTCE----------**

**CC1G_10336.3 (103) L----DKKKYVGMICAGSLAARTAKLPKQP-------ITSHPSVRGDLEA---DFEYSEDP-VVVSG--TLVTSRGPGTAFPFALTLVELLCGKEKREEVRSPMVFPTGTPF----------**

**Dicsq1_101983 (108) L----EAGKYVGMICAGSLAALTAKLPKQP-------LTSHPSVKDRLAD---AFVYSEQS-VVVSG--KLVTSRGPGTTFPFAFTLVEMLFGKEKREEVIGPMVFPPGTWA----------**

**Gansp1_117607 (107) L----QAGKYVGMICAGSMAALTSGLPKQP-------LTSHPSVKDRLKD---AFEYSEQS-VVISG--KLVTSRGPGTAFPFAFTLVQLLFGKEKRAEVEGPMIFPPGTTFS---------**

**Hetan2_436865 (107) Y----EQKKIVGMICAGSMAALTSKLPKQP-------LTSHPSVKSKLET---EYDYREDS-VVVSG--NLITSRGPGTAFPFALTLIEHLCGATKREEVQGPMMFPPETTW----------**

**HirDJ1 (107) Y----EQKKIVGMICAGSMAALTSKLPKQP-------LTSHPSVKSKLET---EYDYREDS-VVVSG--NLITSRGPGTAFPFALTLIEHLCGATKREEVQGPMMFPPETTW----------**

**MGL_3627 (105) H----ANGKIVGMICAGSLAALEARVGLGG-P-----ITSHPSVKDKLAS---CTYQYQELPVAVSN--NLVTSRGPGTTFLFALTLVEKLMGIDKRQEITGPMMLTPEHL-----------**

**OolDJ1 (102) I----ESKKIVGMICAGSLAALTSALPKQP-------LTSHPSVKSQLEE---DFTYSEDS-VVISK--NLVTSRGPGTAFPFALKLVELLCGSEKRAEVYGPMVFPSGTPW----------**

**Phaca1_160579 (105) L----DEGKFVGMICAGSLAAKTSGLAKQP-------ITSHPSVKSELEK---NFDYREDP-VVVSG--KLVTSRGPGTTFPFALTLVELLCGAEKRKDVAGPMVFPPNTFA----------**

**Phchr1_3440 (105) L----DEGKFVGMICAGSLAAKTSGLPKQP-------ITSHPSVKSQLEK---DFDYREEP-VVVSG--KLVTSRGPGTTFPFALTLVELLCGTEKRKEVAGPMVFPPNTFA----------**

**POSPLDRAFT_103847 (105) L----QEDKYVGMICAGSLAAQTSKLPRQP-------LTSHPSVQAELEK---DFEYSDAP-VVVSG--KLVTSWRTGTAFPFALTLVELLCGAPARAEVAGPMVFPAGTFA----------**

**Punst1_52328 (103) Y----DQGKVVGMICAGSLAALSAKLPKQQ-------ITSHPSVRSKLEK---EFEYSEDP-VALSG--KLVTSRGPGTAFPFALTLAELLVGKSKRDEIYGPMVFPHGTPF----------**

**MviDJ1 (99) Y----DQGKLVGMICAGSLAAKTSGIAGGQ-R-----ITSHPSVKGDLEK---HYDYVEDR-VVVSG--NLVTSRGPGTALEWALTIVNILAGSAKRSEVEGPLMM----------------**

**RTG_01234 (107) W----SEQKLVGCICAGSLAALSSQIGLGG-A-----LTSHPSVRSQLEK---HYDYSDDR-VVVAG--NLVTSRGPGTALEWALQLVEILAGRKKRDEVEGPMMV----------------**

**Rhoba1_1_52552 (116) Y----DDGKLVACICAGSLAAKTAGIGLGG-R-----ITSHPSVRDDLEG---VYDYVDDERVVVEA--NLVTSRGPGTALEWALAIVEILAGEKRRDEVAGPMML----------------**

**SCHCODRAFT_58862 (102) I----DAGKYVGMICAGSLAAQTSKLPKQP-------LTSHPSVKAQLEG---DYEYSESP-VVVSG--KLVTSRGPGTAFPFALTLVELLLGKDKREEVRGPMAFPANTPF----------**

**SERLA73DRAFT_191001 (106) Y----KHNKIVAMICAGSLAAKRSGLPRQP-------LTSHPSVKADLER---DFDYSEDS-VVISG--KLVTSRGPGTAFPFAFALVELLCGPEKRAEVRGPMVFPSGAPF----------**

**Stehi1_124932 (108) Y----NNKKLVAMICAGSLAALSSELPSQP-------LTSHPSVKSELDQ---KFKYSEDS-VVVSN--NLITSRGPGTAFPFALTIVELLCGKEKREQVRGPMIFPAGTPF----------**

**Trave1_171260 (108) L----QAGKFVGMICAGSLAASTAGLPRQP-------ITSHPSVRDQLKD---DFDYSEES-VVISG--KLVTSRGPGTTFPFALTLVELLCGKEKRAEVAGPMVFPQGTFA----------**

**Um10481 (108) Y----AKGKVVGAICAGSLAIHSAAIARDS-A-----ITSHPSVKSSLD----QHYAYKDDRVVVAD--NLITSRGPGTTFEFALALLDALVGKHNRLKIQPPMILHPSMSAHAQPEPDQLN**

**Walse1_59511 (96) Y---QVKGKLLGTICAGSLAIKSSGIAKGK-A-----ITSHPSVKGELTG---DYAYSEDK-VVVTD--NLVSSRGPGTAFPFALTLVELALGKEKRDEVAGPMVF----------------**

**AMAG_03424.1 (101) LTAT-DKCRHVAIICASPVALVPALASLSLPAD--LRITSHPCVQAEIEAGLPKAKYVEDR-VVSSWDGRLITSRGPGTAYEFALAIVETLVGKEVHDKVAAPMLLPQL-------------**

**AMAG_04742.1 (101) LTAT-DKCRHVAIICASPVALVPALASLSLPAD--LRITSHPCVQAEIEAGLPKAKYVEDR-VVSTWNGRLITSRGPGTAYEFALAIVETLVGKEVHDKVAAPMLLPQL-------------**

**BDEG_07033 (93) -LAN-TNGKLIGVICAAPIALKAAGILFGK-R-----LTSHPSVKDQLES---NYQYSDDR-VVVDG--NLITSRGPGTAIDFALALVEKLLGAQVRSKVEAPMCIC---------------**

**SPPG_04405.2 (92) -QYY-SQQKVVAIICASPIVLKAAQVAKGR-S-----VTAHPSVKDQLVQ---DYNYKEER-VVVDG--NLITSRGPGTAFEFALAVVKKLQGPEKLKEIVPPMICNDEIVKSV--------**

**Mucci2_157438 (97) --FY-NSKKIVAFVCAGTLVAKAAGIPGKH-T-----VTSYPAVKGQLDN---VYTYSDDR-VVVDD--NVITSRSPGTSFLFALTIVEQLVDVKTANLLRDEMLTCSSL------------**

**RO3G_06344.3 (97) --FY-EHAKLVAFICAGTLVAKEAGIPAHH-T-----VTSFPGVKNQLTG---VYTYSEDR-VVVSD--NVITSRAPGTAFLFALTIAEKLVGSEIVDTVKHDLLTLPEL------------**

**Phybl2_131210 (98) --LY-DQKKIVAFICAGTLVAKASGIPKGH-T-----VTSYPAVMDQLTD---TYEYSQER-VVVDK--NVITSRAPGTSFLFALTIVENLVGKEAADQLKKDMLTSSVL------------**

*** ***
