# Supplementary material for: Identification of glutathione (GSH)-independent glyoxalase III from Schizosaccharomyces pombe
Source: BMC Evol Biol. 2014 Apr 23;14:86. doi: 10.1186/1471-2148-14-86 (PMC4021431; doi:10.1186/1471-2148-14-86)
Supplement: Additional file 2 — Percentage amino acid identities of fungal DJ-1 proteins compared with those from C. elegans (cDJR-1.1), D. melanogaster (DmDJ-1α), A. thaliana (AtDJ-1A), and human (HsDJ-1), E. coli YajL (EcYajL), and E. coli Hsp31 (EcHsp31). The first column is accession numbers (For proteins lacking a accession number, species names are used). See Table 1 for details. The pair-wise percent identity scores were generated with Clustal W [91]. For AtDJ-1A, only the C-terminal region is used for comparison. [file 1471-2148-14-86-S2.doc]

Additional file 2. Percentage amino acid identities of fungal DJ-1 proteins compared with those from *C. elegans* (cDJR-1.1), *D. melanogaster* (DmDJ-1α), *A. thaliana* (AtDJ-1A), and human (HsDJ-1), *E. coli* YajL (EcYajL), and *E. coli* Hsp31 (EcHsp31).

| Fungal DJ-1 | HsDJ-1 | | DmDJ-1α | | cDJR-1.1 | | AtDJ-1A | | EcYajL | | EcHsp31 | |
| --- | --- | --- | --- | --- | --- | --- | --- | --- | --- | --- | --- | --- |
|  | I% | S% | I% | S% | I% | S% | I% | S% | I% | S% | I% | S% |
| *A. arborescens* | 38 | 55 | 32 | 40 | 37 | 54 | 29 | 45 | 32 | 49 | 15 | 27 |
| Altbr1_7229 | 39 | 57 | 29 | 41 | 39 | 57 | 30 | 47 | 33 | 53 | 13 | 25 |
| CocheC4_1_31923 | 38 | 55 | 31 | 42 | 40 | 54 | 31 | 49 | 31 | 50 | 13 | 25 |
| Coclu2_103587 | 36 | 56 | 31 | 43 | 40 | 55 | 30 | 47 | 32 | 51 | 14 | 25 |
| Cocmi1_87889 | 37 | 56 | 31 | 42 | 40 | 55 | 31 | 49 | 30 | 50 | 14 | 26 |
| Cocsa1_181323 | 38 | 55 | 31 | 42 | 40 | 56 | 30 | 48 | 32 | 51 | 13 | 25 |
| Cocvi1_89530 | 39 | 56 | 31 | 42 | 41 | 56 | 31 | 49 | 31 | 50 | 14 | 27 |
| PTT_13806 | 38 | 54 | 32 | 43 | 40 | 53 | 28 | 47 | 31 | 49 | 13 | 27 |
| PTRG_06163.1 | 39 | 56 | 33 | 44 | 40 | 55 | 29 | 47 | 33 | 52 | 13 | 27 |
| SNOG_07399 | 37 | 53 | 32 | 44 | 40 | 58 | 30 | 49 | 33 | 51 | 13 | 25 |
| SJAG_06414.4 | 29 | 44 | 25 | 39 | 29 | 47 | 22 | 41 | 26 | 40 | 12 | 22 |
| SJAG_02106.4 | 23 | 41 | 23 | 40 | 25 | 45 | 23 | 39 | 25 | 44 | 11 | 23 |
| SPAC22E12.03c | 27 | 45 | 25 | 39 | 31 | 49 | 24 | 44 | 23 | 43 | 9 | 20 |
| SOCG_00579.5 | 24 | 42 | 25 | 37 | 29 | 48 | 22 | 41 | 24 | 43 | 10 | 21 |
| SPOG_01926.3 | 26 | 42 | 25 | 37 | 29 | 48 | 24 | 41 | 25 | 43 | 10 | 22 |
| Agabi_187606 | 38 | 55 | 32 | 40 | 37 | 55 | 26 | 42 | 31 | 51 | 12 | 23 |
| Aurde1_110250 | 35 | 50 | 33 | 46 | 41 | 57 | 34 | 47 | 35 | 52 | 12 | 22 |
| Cersu1_140432 | 31 | 47 | 30 | 43 | 35 | 53 | 27 | 38 | 31 | 48 | 11 | 20 |
| CC1G_10336.3 | 34 | 50 | 30 | 43 | 38 | 57 | 28 | 43 | 31 | 49 | 9 | 22 |
| Dicsq1_101983 | 32 | 47 | 31 | 43 | 36 | 55 | 28 | 40 | 31 | 48 | 12 | 23 |
| Gansp1_117607 | 31 | 47 | 31 | 43 | 35 | 55 | 28 | 40 | 32 | 48 | 12 | 20 |
| Hetan2_436865 | 31 | 46 | 32 | 43 | 39 | 52 | 27 | 38 | 30 | 44 | 12 | 24 |
| *H. irregular* | 31 | 46 | 32 | 43 | 39 | 52 | 27 | 38 | 30 | 44 | 12 | 24 |
| MGL_3627 | 37 | 51 | 32 | 44 | 39 | 51 | 26 | 40 | 29 | 48 | 14 | 23 |
| *M. violaceum* | 31 | 49 | 28 | 42 | 38 | 57 | 29 | 45 | 28 | 46 | 10 | 21 |
| *O. olearius* | 34 | 51 | 31 | 46 | 39 | 57 | 27 | 42 | 31 | 48 | 11 | 20 |
| Phaca1_160579 | 29 | 45 | 31 | 44 | 37 | 55 | 24 | 38 | 29 | 46 | 11 | 20 |
| Phchr1_3440 | 28 | 43 | 31 | 43 | 36 | 54 | 26 | 42 | 27 | 45 | 12 | 21 |
| POSPLDRAFT_103847 | 31 | 46 | 29 | 40 | 35 | 51 | 25 | 39 | 31 | 47 | 13 | 23 |
| Punst1_52328 | 34 | 49 | 28 | 41 | 28 | 41 | 26 | 41 | 34 | 50 | 11 | 23 |
| RTG_01234 | 34 | 49 | 29 | 43 | 37 | 55 | 29 | 46 | 28 | 48 | 12 | 23 |
| Rhoba1_1_52552 | 31 | 46 | 31 | 44 | 33 | 54 | 30 | 49 | 27 | 45 | 12 | 21 |
| SCHCODRAFT_58862 | 33 | 49 | 30 | 45 | 39 | 58 | 30 | 43 | 31 | 52 | 10 | 21 |
| SERLA73DRAFT_191001 | 33 | 48 | 29 | 43 | 36 | 53 | 26 | 38 | 32 | 48 | 12 | 22 |
| Stehi1_124932 | 37 | 51 | 32 | 41 | 39 | 55 | 29 | 41 | 32 | 46 | 13 | 24 |
| Trave1_171260 | 33 | 49 | 31 | 47 | 36 | 55 | 28 | 40 | 30 | 48 | 13 | 21 |
| Um10481 | 31 | 47 | 31 | 43 | 35 | 49 | 25 | 41 | 30 | 47 | 12 | 22 |
| Walse1_59511 | 37 | 53 | 36 | 47 | 42 | 56 | 28 | 48 | 27 | 44 | 11 | 21 |
| AMAG_03424.1 | 43 | 54 | 37 | 47 | 41 | 56 | 32 | 49 | 34 | 49 | 11 | 21 |
| AMAG_04742.1 | 42 | 53 | 37 | 46 | 40 | 56 | 32 | 49 | 34 | 49 | 10 | 21 |
| BDEG_07033 | 43 | 56 | 39 | 50 | 44 | 58 | 35 | 55 | 32 | 53 | 13 | 24 |
| SPPG_04405.2 | 43 | 57 | 38 | 52 | 40 | 56 | 38 | 53 | 37 | 56 | 14 | 23 |
| RO3G_06344.3 | 41 | 52 | 36 | 51 | 39 | 54 | 29 | 43 | 29 | 46 | 12 | 20 |
| Mucci2_157438 | 40 | 54 | 36 | 48 | 37 | 55 | 29 | 45 | 28 | 45 | 12 | 21 |
| Phybl2_131210 | 41 | 52 | 36 | 49 | 37 | 54 | 34 | 48 | 30 | 50 | 11 | 21 |
