# Supplementary material for: Identification of glutathione (GSH)-independent glyoxalase III from Schizosaccharomyces pombe
Source: BMC Evol Biol. 2014 Apr 23;14:86. doi: 10.1186/1471-2148-14-86 (PMC4021431; doi:10.1186/1471-2148-14-86)
Supplement: Additional file 3 — Distribution of candidate Hsp31 proteins identified in fungi. +The number of amino acids in fungal Hsp31. *Indicates that mispredicted sequences obtained from the databases have been corrected. ?Indicates the sequence could not be correctly predicted due to sequence gaps. “―”, the protein sequence could not be identified by BLAST searches. “#”, the accession number is not available. [file 1471-2148-14-86-S3.doc]

Additional file 3. Distribution of candidate Hsp31 proteins in fungi.

| **Species** | **Phylum/subphylum** | **Accession number** | **Database** | **No. aa^+^** | **Class type** |
| --- | --- | --- | --- | --- | --- |
| *Ajellomyces capsulatus* | Ascomycota/Pezizomycotina | HCDG_00904 | NCBI | 232 | Class II |
| *Ajellomyces dermatitidis* | Ascomycota/Pezizomycotina | BDBG_02344 | NCBI | 231 | Class II |
| *Arthrobotrys oligospora* | Ascomycota/Pezizomycotina | AOL_s00109g140 | NCBI | 260 | Class II |
| *Arthroderma benhamiae* | Ascomycota/Pezizomycotina | ARB_05407 | NCBI | 240 | Class II |
| *Arthroderma gypseum* | Ascomycota/Pezizomycotina | MGYG_01284 | NCBI | 240 | Class II |
| *Arthroderma otae* | Ascomycota/Pezizomycotina | MCYG_00686 | NCBI | 249 | Class II |
| *Aspergillus clavatus* | Ascomycota/Pezizomycotina | ACLA_003820 | NCBI | 236 | Class II |
| *Aspergillus clavatus* | Ascomycota/Pezizomycotina | ACLA_063760 | NCBI | 253 | Class III |
| *Aspergillus clavatus* | Ascomycota/Pezizomycotina | ACLA_067030 | NCBI | 239 | Class II |
| *Aspergillus flavus* | Ascomycota/Pezizomycotina | AFLA_014130 | NCBI | 239* | Class II |
| *Aspergillus flavus* | Ascomycota/Pezizomycotina | AFLA_019490 | NCBI | 234* | Class II |
| *Aspergillus flavus* | Ascomycota/Pezizomycotina | AFLA_124160 | NCBI | 230 | Class II |
| *Aspergillus flavus* | Ascomycota/Pezizomycotina | AFLA_138590 | NCBI | 253 | Class III |
| *Aspergillus fumigatus* | Ascomycota/Pezizomycotina | AFUA_3G01210 | NCBI | 253 | Class III |
| *Aspergillus fumigatus* | Ascomycota/Pezizomycotina | AFUA_5G01430 | NCBI | 236 | Class II |
| *Aspergillus* *nidulans* | Ascomycota/Pezizomycotina | AN6796.2 | NCBI | 237* | Class II |
| *Aspergillus nidulans* | Ascomycota/Pezizomycotina | AN6810.2 | NCBI | 256* | Class III |
| *Aspergillus niger* | Ascomycota/Pezizomycotina | ANI_1_114144 | NCBI | 234 | Class II |
| *Aspergillus niger* | Ascomycota/Pezizomycotina | ANI_1_1122094 | NCBI | 239 | Class II |
| *Aspergillus niger* | Ascomycota/Pezizomycotina | ANI_1_1276124 | NCBI | 169 | Class II |
| *Aspergillus niger* | Ascomycota/Pezizomycotina | ANI_1_1478014 | NCBI | 248 | Class III |
| *Aspergillus niger* | Ascomycota/Pezizomycotina | ANI_1_1764104 | NCBI | 232 | Class II |
| *Aspergillus terreus* | Ascomycota/Pezizomycotina | ATEG_07760 | NCBI | 253 | Class III |
| *Aspergillus terreus* | Ascomycota/Pezizomycotina | ATEG_09753 | NCBI | 234 | Class II |
| *Botryotinia fuckeliana* | Ascomycota/Pezizomycotina | BC1G_16047 | NCBI | 130? | Class II |
| *Chaetomium globosum* | Ascomycota/Pezizomycotina | CHGG_04205 | NCBI | 230 | Class II |
| *Chaetomium thermophilum* | Ascomycota/Pezizomycotina | EGS21780.1 | NCBI | 236 | Class II |
| *Coccidioides immitis* | Ascomycota/Pezizomycotina | CIMG_03805 | NCBI | 243 | Class II |
| *Coccidioides posadasii* | Ascomycota/Pezizomycotina | CPC735_005110 | NCBI | 243 | Class II |
| *Cordyceps militaris* | Ascomycota/Pezizomycotina | CCM_05507 | NCBI | 296 | Class I |
| *Cordyceps militaris* | Ascomycota/Pezizomycotina | CCM_09248 | NCBI | 225 | Class II |
| *Fusarium oxysporum* | Ascomycota/Pezizomycotina | FOXB_01392 | NCBI | 234* | Class II |
| *Fusarium oxysporum* | Ascomycota/Pezizomycotina | FOXB_05365 | NCBI | 237* | Class II |
| *Fusarium oxysporum* | Ascomycota/Pezizomycotina | FOXB_05842 | NCBI | 239 | Class II |
| *Fusarium oxysporum* | Ascomycota/Pezizomycotina | FOXB_06919 | NCBI | 226 | Class II |
| *Gibberella zeae* | Ascomycota/Pezizomycotina | FG08979.1 | NCBI | 226 | Class II |
| *Glomerella graminicola* | Ascomycota/Pezizomycotina | GLRG_10735 | NCBI | 229 | Class II |
| *Magnaporthe oryzae* | Ascomycota/Pezizomycotina | MGG_01679 | NCBI | 231 | Class II |
| *Metarhizium acridum* | Ascomycota/Pezizomycotina | MAC_07323 | NCBI | 228 | Class II |
| *Metarhizium acridum* | Ascomycota/Pezizomycotina | MAC_05717 | NCBI | 294 | Class I |
| *Metarhizium anisopliae* | Ascomycota/Pezizomycotina | MAA_08674 | NCBI | 294 | Class I |
| *Metarhizium anisopliae* | Ascomycota/Pezizomycotina | MAA_09738 | NCBI | 228* | Class II |
| *Myceliophthora thermophila* | Ascomycota/Pezizomycotina | MYCTH_2310610 | NCBI | 244 | Class II |
| *Mycosphaerella graminicola* | Ascomycota/Pezizomycotina | MYCGRDRAFT_77354 | NCBI | 236 | Class II |
| *Nectria haematococca* | Ascomycota/Pezizomycotina | NECHADRAFT_42326 | NCBI | 234 | Class II |
| *Nectria haematococca* | Ascomycota/Pezizomycotina | NECHADRAFT_45064 | NCBI | 239 | Class II |
| *Nectria haematococca* | Ascomycota/Pezizomycotina | NECHADRAFT_49514 | NCBI | 239 | Class II |
| *Nectria haematococca* | Ascomycota/Pezizomycotina | NECHADRAFT_60634 | NCBI | 237 | Class II |
| *Nectria haematococca* | Ascomycota/Pezizomycotina | NECHADRAFT_62574 | NCBI | 232 | Class II |
| *Nectria haematococca* | Ascomycota/Pezizomycotina | NECHADRAFT_81418 | NCBI | 233 | Class II |
| *Nectria haematococca* | Ascomycota/Pezizomycotina | NECHADRAFT_83491 | NCBI | 233 | Class II |
| *Neosartorya fischeri* | Ascomycota/Pezizomycotina | NFIA_002150 | NCBI | 253 | Class III |
| *Neosartorya fischeri* | Ascomycota/Pezizomycotina | NFIA_040680 | NCBI | 236 | Class II |
| *Neurospora crassa* | Ascomycota/Pezizomycotina | NCU06603 | NCBI | 242 | Class II |
| *Neurospora tetrasperma* | Ascomycota/Pezizomycotina | NEUTE2DRAFT_85759 | NCBI | 242 | Class II |
| *Paracoccidioides brasiliensis* | Ascomycota/Pezizomycotina | PAAG_03106 | NCBI | 232 | Class II |
| *Penicillium chrysogenum* | Ascomycota/Pezizomycotina | Pc12g09930 | NCBI | 252 | Class III |
| *Penicillium chrysogenum* | Ascomycota/Pezizomycotina | Pc20g03290 | NCBI | 235 | Class II |
| *Penicillium marneffei* | Ascomycota/Pezizomycotina | PMAA_010240 | NCBI | 239 | Class II |
| *Penicillium marneffei* | Ascomycota/Pezizomycotina | PMAA_013250 | NCBI | 236 | Class II |
| *Phaeosphaeria nodorum* | Ascomycota/Pezizomycotina | SNOG_00505 | NCBI | 262 | Class III |
| *Phaeosphaeria nodorum* | Ascomycota/Pezizomycotina | SNOG_04306 | NCBI | 229 | Class II |
| *Pyrenophora teres* | Ascomycota/Pezizomycotina | PTT_13641 | NCBI | 229 | Class II |
| *Pyrenophora teres* | Ascomycota/Pezizomycotina | PTT_19431 | NCBI | 252 | Class III |
| *Pyrenophora tritici-repentis* | Ascomycota/Pezizomycotina | PTRG_04958 | NCBI | 229 | Class II |
| *Pyrenophora tritici-repentis* | Ascomycota/Pezizomycotina | PTRG_10645 | NCBI | 252 | Class III |
| *Sclerotinia sclerotiorum* | Ascomycota/Pezizomycotina | SS1G_06318 | NCBI | 238 | Class II |
| *Talaromyces stipitatus* | Ascomycota/Pezizomycotina | TSTA_003770 | NCBI | 236 | Class II |
| *Talaromyces stipitatus* | Ascomycota/Pezizomycotina | TSTA_105480 | NCBI | 247 | Class III |
| *Thielavia terrestris* | Ascomycota/Pezizomycotina | THITE_2114472 | NCBI | 244 | Class II |
| *Trichoderma reesei* | Ascomycota/Pezizomycotina | TRIREDRAFT_59940 | NCBI | 229 | Class II |
| *Trichophyton equinum* | Ascomycota/Pezizomycotina | TEQG_08419 | NCBI | 240 | Class II |
| *Trichophyton rubrum* | Ascomycota/Pezizomycotina | TERG_00228 | NCBI | 222 | Class II |
| *Trichophyton tonsurans* | Ascomycota/Pezizomycotina | TESG_07748 | NCBI | 240 | Class II |
| *Trichophyton verrucosum* | Ascomycota/Pezizomycotina | TRV_03863 | NCBI | 240 | Class II |
| *Uncinocarpus reesii* | Ascomycota/Pezizomycotina | UREG_07480 | NCBI | 242 | Class II |
| *Verticillium albo-atrum* | Ascomycota/Pezizomycotina | VDBG_05153 | NCBI | 274 | Class I |
| *Verticillium albo-atrum* | Ascomycota/Pezizomycotina | VDBG_07599 | NCBI | 258 | Class II |
| *Verticillium dahliae* | Ascomycota/Pezizomycotina | VDAG_08958 | NCBI | 293 | Class I |
| *Verticillium dahliae* | Ascomycota/Pezizomycotina | VDAG_09321 | NCBI | 235 | Class II |
| *Ashbya gossypii* | Ascomycota/Saccharomycotina | - |  |  |  |
| *Candida albicans* | Ascomycota/Saccharomycotina | CaO19.251 | NCBI | 236 | Class II |
| *Candida albicans* | Ascomycota/Saccharomycotina | CaO19.7882 | NCBI | 236 | Class II |
| *Candida dubliniensis* | Ascomycota/Saccharomycotina | CD36_82570 | NCBI | 236 | Class II |
| *Candida glabrata* | Ascomycota/Saccharomycotina | CAGL0C00275g | NCBI | 236 | Class II |
| *Candida tenuis* | Ascomycota/Saccharomycotina | CANTEDRAFT_115959 | NCBI | 235 | Class II |
| *Candida tropicalis* | Ascomycota/Saccharomycotina | CTRG_02436 | NCBI | 236* | Class II |
| *Candida tropicalis* | Ascomycota/Saccharomycotina | CTRG_02543 | NCBI | 235 | Class II |
| *Clavispora lusitaniae* | Ascomycota/Saccharomycotina | CLUG_02395 | NCBI | 233 | Class II |
| *Debaryomyces hansenii* | Ascomycota/Saccharomycotina | DEHA2E03762g | NCBI | 239 | Class II |
| *Debaryomyces hansenii* | Ascomycota/Saccharomycotina | DEHA2E17270g | NCBI | 241 | Class II |
| *Debaryomyces hansenii* | Ascomycota/Saccharomycotina | DEHA2G01474g | NCBI | 236 | Class II |
| [*Eremothecium cymbalariae*](http://www.ncbi.nlm.nih.gov/bioproject?term=txid931890%5Borgn%5D) | Ascomycota/Saccharomycotina | - | NCBI |  |  |
| *Kluyveromyces lactis* | Ascomycota/Saccharomycotina | KLLA0D00682g | NCBI | 237 | Class II |
| *Kluyveromyces lactis* | Ascomycota/Saccharomycotina | KLLA0D00704g | NCBI | 237 | Class II |
| *Komagataella pastoris* | Ascomycota/Saccharomycotina | PAS_chr3_0691 | NCBI | 229 | Class II |
| *Lachancea thermotolerans* | Ascomycota/Saccharomycotina | KLTH0A00242g | NCBI | 193 | Class II |
| *Lodderomyces elongisporus* | Ascomycota/Saccharomycotina | LELG_02042 | NCBI | 237 | Class II |
| *Lodderomyces elongisporus* | Ascomycota/Saccharomycotina | LELG_02043 | NCBI | 235 | Class II |
| *ILodderomyces elongisporus* | Ascomycota/Saccharomycotina | LELG_02044 | NCBI | 237 | Class II |
| *Meyerozyma guilliermondii* | Ascomycota/Saccharomycotina | PGUG_04657 | NCBI | 244* | Class II |
| *Naumovozyma castellii* | Ascomycota/Saccharomycotina | - |  |  |  |
| *Naumovozyma dairenensis* | Ascomycota/Saccharomycotina | - |  |  |  |
| *Ogataea parapolymorpha* | Ascomycota/Saccharomycotina | - |  |  |  |
| *Saccharomyces cerevisiae* | Ascomycota/Saccharomycotina | YDR533C | NCBI | 237 | Class II |
| *Saccharomyces cerevisiae* | Ascomycota/Saccharomycotina | YPL280W | NCBI | 237 | Class II |
| *Saccharomyces cerevisiae* | Ascomycota/Saccharomycotina | YOR391C | NCBI | 237 | Class II |
| *Saccharomyces cerevisiae* | Ascomycota/Saccharomycotina | YMR322C | NCBI | 237 | Class II |
| *Scheffersomyces stipitis* | Ascomycota/Saccharomycotina | PICST_59509 | NCBI | 236 | Class II |
| *Spathaspora passalidarum* | Ascomycota/Saccharomycotina | SPAPADRAFT_51238 | NCBI | 236 | Class II |
| *Tetrapisispora phaffii* | Ascomycota/Saccharomycotina | - | NCBI |  |  |
| *Torulaspora delbrueckii* | Ascomycota/Saccharomycotina | TDEL0F00110 | NCBI | 234 | Class II |
| *Vanderwaltozyma polyspora* | Ascomycota/Saccharomycotina | Kpol_196p3 | NCBI | Gap | Class II |
| *Vanderwaltozyma polyspora* | Ascomycota/Saccharomycotina | Kpol_1058p1 | NCBI | 237 | Class II |
| *Yarrowia lipolytica* | Ascomycota/Saccharomycotina | YALI0C22000p | NCBI | 239 | Class II |
| *Yarrowia lipolytica* | Ascomycota/Saccharomycotina | YALI0F00682p | NCBI | 250 | Class II |
| *Zygosaccharomyces rouxii* | Ascomycota/Saccharomycotina | - |  |  |  |
| *Schizosaccharomyces pombe* | Ascomycota/Taphrinomycotina | SPAC5H10.02c | NCBI | 240 | Class II |
| *Schizosaccharomyces pombe* | Ascomycota/Taphrinomycotina | SPBC947.09 | NCBI | 262 | Class II |
| *Schizosaccharomyces pombe* | Ascomycota/Taphrinomycotina | SPCC757.03c | NCBI | 244 | Class II |
| *Schizosaccharomyces pombe* | Ascomycota/Taphrinomycotina | SPAC1F7.06 | NCBI | 251 | Class II |
| *Schizosaccharomyces pombe* | Ascomycota/Taphrinomycotina | SPAC11D3.13 | NCBI | 222 | Class II |
| *Schizosaccharomyces japonicus* | Ascomycota/Taphrinomycotina | SJAG_02988 | NCBI | 238 | Class II |
| *Schizosaccharomyces japonicus* | Ascomycota/Taphrinomycotina | SJAG_0467 | NCBI | 243 | Class II |
| [*Schizosaccharomyces octosporus*](http://www.ncbi.nlm.nih.gov/bioproject?term=txid483514%5Borgn%5D) | Ascomycota/Taphrinomycotina | SOCG_04619.1 | Broad | 245 | Class II |
| [*Schizosaccharomyces octosporus*](http://www.ncbi.nlm.nih.gov/bioproject?term=txid483514%5Borgn%5D) | Ascomycota/Taphrinomycotina | SOCG_01701.1 | Broad | 238 | Class II |
| *Schizosaccharomyces cryophilus* | Ascomycota/Taphrinomycotina | SPOG_03630.1 | Broad | 242 | Class II |
| *Schizosaccharomyces cryophilus* | Ascomycota/Taphrinomycotina | SPOG_03829.1 | Broad | 238 | Class II |
| *Coprinopsis cinerea* | Basidiomycota/Agaricomycotina | CC1G_10162 | NCBI | 270 | Class III |
| *Coprinopsis cinerea* | Basidiomycota/Agaricomycotina | CC1G_11702 | NCBI | 231 | Class II |
| *Coprinopsis cinerea* | Basidiomycota/Agaricomycotina | CC1G_00260 | NCBI | 230 | Class II |
| *Cryptococcus gattii* | Basidiomycota/Agaricomycotina | CGB_C3010C | NCBI | 233 | Class II |
| *Cryptococcus gattii* | Basidiomycota/Agaricomycotina | CGB_E6750W | NCBI | 252 | Class II |
| *Cryptococcus neoformans* | Basidiomycota/Agaricomycotina | CNC01950 | NCBI | 233 | Class II |
| *Cryptococcus neoformans* | Basidiomycota/Agaricomycotina | CNJ00030 | NCBI | 252 | Class II |
| *Laccaria bicolor* | Basidiomycota/Agaricomycotina | - |  |  |  |
| *Moniliophthora perniciosa* | Basidiomycota/Agaricomycotina | - |  |  |  |
| *Postia placenta* | Basidiomycota/Agaricomycotina | Pospl1_110200 | JGI | 224 | Class II |
| *Postia placenta* | Basidiomycota/Agaricomycotina | Pospl1_115118 | JGI | 249 | Class II |
| *Schizophyllum commune* | Basidiomycota/Agaricomycotina | SCHCODRAFT_46162 | NCBI | 226 | Class II |
| *Schizophyllum commune* | Basidiomycota/Agaricomycotina | SCHCODRAFT_49614 | NCBI | 225 | Class II |
| *Serpula lacrymans* | Basidiomycota/Agaricomycotina | SERLA73DRAFT_120613 | NCBI | 224 | Class II |
| *Melampsora larici-populina* | Basidiomycota/Puccciniomycotina | - |  |  |  |
| *Puccinia graminis* | Basidiomycota/Puccciniomycotina | - |  |  |  |
| *Rhodotorula glutinis* | Basidiomycota/Puccciniomycotina | # | NCBI | 230 | Class II |
| [*Rhodotorula graminis*](http://genome.jgi.doe.gov/Rhoba1_1) | Basidiomycota/Puccciniomycotina | Rhoba1_1_64353 | JGI | 232 | Class II |
| *Malassezia globosa* | Basidiomycota/Ustilaginomycotina | MGL_4192 | NCBI | 240 | Class II |
| *Ustilago maydis* | Basidiomycota/Ustilaginomycotina | UM00094.1 | NCBI | 235 | Class II |
| *Allomyces macrogynus* | Chytridiomycota/Chytridiomycetes | - |  |  |  |
| *Batrachochytrium dendrobatidis* | Chytridiomycota/Chytridiomycetes | - |  |  |  |
| *Spizellomyces punctatus* | Chytridiomycota/Chytridiomycetes | SPPG_02734.3 | Broad | 260 | Class II |
| *Spizellomyces punctatus* | Chytridiomycota/Chytridiomycetes | SPPG_05672.3 | Broad | 231 | Class II |
| *Rhizopus oryzae* | Zygomycota/Mucormycotina | RO3G_07202.3 | Broad | 240 | Class II |
| *Mucor circinelloides* | Zygomycota/Mucormycotina | Mucci2_157529 | JGI | 240 | Class II |
| *Phycomyces blakesleeanus* | Zygomycota/Mucormycotina | Phybl2_109595 | JGI | 243 | Class II |
