# Supplementary material for: Identification of glutathione (GSH)-independent glyoxalase III from Schizosaccharomyces pombe
Source: BMC Evol Biol. 2014 Apr 23;14:86. doi: 10.1186/1471-2148-14-86 (PMC4021431; doi:10.1186/1471-2148-14-86)
Supplement: Additional file 4 — The number of Hsp31 proteins in fungal species. [file 1471-2148-14-86-S4.doc]

Additional file 4. The number of Hsp31 proteins in fungal species

| Species | Taxonomy | Number |
| --- | --- | --- |
| **Ascomycota** |  |  |
| *Chaetomium globosum* | Pezizomycotina**/**Sordariomycete | 1 |
| *Chaetomium thermophilum* | Pezizomycotina**/**Sordariomycete | 1 |
| *Cordyceps militaris* | Pezizomycotina**/**Sordariomycete | 2 |
| *Fusarium oxysporum* | Pezizomycotina**/**Sordariomycete | 4 |
| *Gibberella zeae* | Pezizomycotina**/**Sordariomycete | 1 |
| *Glomerella graminicola* | Pezizomycotina**/**Sordariomycete | 1 |
| *Magnaporthe oryzae* | Pezizomycotina**/**Sordariomycete | 1 |
| *Metarhizium acridum* | Pezizomycotina**/**Sordariomycete | 2 |
| *Metarhizium anisopliae* | Pezizomycotina**/**Sordariomycete | 2 |
| *Myceliophthora thermophila* | Pezizomycotina**/**Sordariomycete | 1 |
| *Mycosphaerella graminicola* | Pezizomycotina**/**Sordariomycete | 1 |
| *Nectria haematococca* | Pezizomycotina**/**Sordariomycete | 7 |
| *Neurospora crassa* | Pezizomycotina**/**Sordariomycete | 1 |
| *Neurospora tetrasperma* | Pezizomycotina**/**Sordariomycete | 1 |
| [*Thielavia terrestris*](http://www.ncbi.nlm.nih.gov/bioproject?term=txid578455%5Borgn%5D) | Pezizomycotina**/**Sordariomycete | 1 |
| *Trichoderma reesei* | Pezizomycotina**/**Sordariomycete | 1 |
| *Verticillium albo-atrum* | Pezizomycotina**/**Sordariomycete | 2 |
| *Verticillium dahliae* | Pezizomycotina**/**Sordariomycete | 2 |
| *Ajellomyces capsulatus* | Pezizomycotina**/**Leotiomycete | 1 |
| *Ajellomyces dermatitidis* | Pezizomycotina/Leotiomycete | 1 |
| *Botryotinia fuckeliana* | Pezizomycotina/Leotiomycete | 1 |
| *Sclerotinia sclerotiorum* | Pezizomycotina/Leotiomycete | 1 |
| *Aspergillus clavatus* | Pezizomycotina/Eurotiomycete | 3 |
| *Aspergillus flavus* | Pezizomycotina/Eurotiomycete | 4 |
| *Aspergillus fumigatus* | Pezizomycotina/Eurotiomycete | 2 |
| *Aspergillus nidulans* | Pezizomycotina/Eurotiomycete | 2 |
| *Aspergillus niger* | Pezizomycotina/Eurotiomycete | 5 |
| *Aspergillus terreus* | Pezizomycotina/Eurotiomycete | 2 |
| *Coccidioides immitis* | Pezizomycotina/Eurotiomycete | 1 |
| [*Coccidioides posadasii*](http://www.ncbi.nlm.nih.gov/bioproject?term=txid199306%5Borgn%5D) | Pezizomycotina/Eurotiomycete | 1 |
| *Neosartorya fischeri* | Pezizomycotina/Eurotiomycete | 3 |
| *Paracoccidioides brasiliensis* | Pezizomycotina/Eurotiomycete | 1 |
| *Penicillium chrysogenum* | Pezizomycotina/Eurotiomycete | 2 |
| *Penicillium marneffei* | Pezizomycotina/Eurotiomycete | 2 |
| *Talaromyces stipitatus* | Pezizomycotina/Eurotiomycete | 2 |
| *Trichophyton equinum* | Pezizomycotina/Eurotiomycete | 1 |
| *Trichophyton rubrum* | Pezizomycotina/Eurotiomycete | 1 |
| *Trichophyton tonsurans* | Pezizomycotina/Eurotiomycete | 1 |
| *Trichophyton verrucosum* | Pezizomycotina/Eurotiomycete | 1 |
| *Uncinocarpus reesii* | Pezizomycotina/Eurotiomycete | 1 |
| *Phaeosphaeria nodorum* | Pezizomycotina/Dothideomycete | 2 |
| *Pyrenophora teres f. teres* | Pezizomycotina/Dothideomycete | 2 |
| *Pyrenophora tritici-repentis* | Pezizomycotina/Dothideomycete | 2 |
| *Arthrobotrys oligospora* | Pezizomycotina/Orbiliomycete | 1 |
| *Arthroderma benhamiae* | Pezizomycotina/Orbiliomycete | 1 |
| *Arthroderma gypseum* | Pezizomycotina/Orbiliomycete | 1 |
| *Arthroderma otae* | Pezizomycotina/Orbiliomycete | 1 |
| *Ashbya gossypii* | Saccharomycotina | 0 |
| *Candida albicans* | Saccharomycotina | 2 |
| *Candida dubliniensis* | Saccharomycotina | 1 |
| *Candida glabrata* | Saccharomycotina | 1 |
| *Candida tenuis* | Saccharomycotina | 1 |
| *Candida tropicalis* | Saccharomycotina | 2 |
| *Clavispora lusitaniae* | Saccharomycotina | 1 |
| *Debaryomyces hansenii* | Saccharomycotina | 4 |
| *Eremothecium cymbalariae* | Saccharomycotina | 0 |
| *Kluyveromyces lactis* | Saccharomycotina | 2 |
| *Komagataella pastoris* | Saccharomycotina | 1 |
| *Lachancea thermotolerans* | Saccharomycotina | 1 |
| *Lodderomyces elongisporus* | Saccharomycotina | 3 |
| *Meyerozyma guilliermondii* | Saccharomycotina | 1 |
| *Naumovozyma castellii* | Saccharomycotina | 0 |
| *Naumovozyma dairenensis* | Saccharomycotina | 0 |
| *Ogataea parapolymorpha* | Saccharomycotina | 0 |
| *Saccharomyces cerevisiae* | Saccharomycotina | 4 |
| *Scheffersomyces stipitis* | Saccharomycotina | 1 |
| *Spathaspora passalidarum* | Saccharomycotina | 1 |
| *Tetrapisispora phaffii* | Saccharomycotina | 0 |
| *Torulaspora delbrueckii* | Saccharomycotina | 1 |
| *Vanderwaltozyma polyspora* | Saccharomycotina | 2 |
| *Yarrowia lipolytica* | Saccharomycotina | 2 |
| *Zygosaccharomyces rouxii* | Saccharomycotina | 0 |
| *Schizosaccharomyces cryophilus* | Taphrinomycotina | 2 |
| *Schizosaccharomyces japonicus* | Taphrinomycotina | 2 |
| [*Schizosaccharomyces octosporus*](http://www.ncbi.nlm.nih.gov/bioproject?term=txid483514%5Borgn%5D) | Taphrinomycotina | 2 |
| *Schizosaccharomyces pombe* | Taphrinomycotina | 3 |
| **Basidiomycota** |  |  |
| *Coprinopsis cinerea* | Agaricomycotina | 3 |
| *Cryptococcus gattii* | Agaricomycotina | 2 |
| *Cryptococcus neoformans* | Agaricomycotina | 2 |
| *Laccaria bicolor* | Agaricomycotina | 0 |
| *Moniliophthora perniciosa* | Agaricomycotina | 0 |
| *Postia placenta* | Agaricomycotina | 2 |
| *Schizophyllum commune* | Agaricomycotina | 2 |
| *Serpula lacrymans* | Agaricomycotina | 1 |
| *Melampsora larici-populina* | Puccciniomycotina | 0 |
| *Puccinia graminis* | Puccciniomycotina | 0 |
| *Rhodotorula glutinis* | Puccciniomycotina | 1 |
| *Rhodotorula graminis* | Puccciniomycotina | 1 |
| *Malassezia globosa* | Ustilaginomycotina | 1 |
| *Ustilago maydis* | Ustilaginomycotina | 1 |
| **Chytridiomycota** |  |  |
| *Allomyces macrogynus* | Blastocladiomycete | 0 |
| *Batrachochytrium dendrobatidis* | Chytridiomycete | 0 |
| *Spizellomyces punctatus* | Chytridiomycete | 2 |
| **Zygomycota** |  |  |
| *Mucor circinellodes* | Mucormycotina | 1 |
| *Phycomyces blakesleeanus* | Mucormycotina | 1 |
| *Rhizopus oryzae* | Mucormycotina | 1 |
