# Supplementary material for: Identification of glutathione (GSH)-independent glyoxalase III from Schizosaccharomyces pombe
Source: BMC Evol Biol. 2014 Apr 23;14:86. doi: 10.1186/1471-2148-14-86 (PMC4021431; doi:10.1186/1471-2148-14-86)
Supplement: Additional file 5 — Multiple sequence alignment of candidate DJ-1 and Hsp31 proteins from representative fungal species. The accession numbers for the candidates are listed in Table 1 and Additional file 3. The annotation of the alignment is described in the legend to Figure 1. [file 1471-2148-14-86-S5.doc]

**Additional file 5**

**ARB_05407 (1) ------------------------------------------------MPLPRRALIAVTSANPHFYPDG---------------KKTGLFFSEALHPFDELQSAG-----FHIDIASETGT**

**TERG_00228 (1) ------------------------------------------------MPLPRKALIAVTSANPHFYPDG---------------KKTGLFFSEALHPFDELQSAG-----FHIDIASETGT**

**TRV_03863 (1) ------------------------------------------------MPLPRRALIAVTSANPHFYPDG---------------KKTGLFFSEALHPFDELQSAG-----FHIDIASETGT**

**TEQG_08419 (1) ------------------------------------------------MPLPRRALIAVTSANPHFYPDG---------------KKTGLFFSEALHPFDELQAAG-----FHIDIASETGT**

**TESG_07748 (1) ------------------------------------------------MPLPRRALIAVTSANPHFYPDG---------------KKTGLFFSEALHPFDELQAAG-----FHIDIASETGT**

**MGYG_01284 (1) ------------------------------------------------MPLPRKALIAVTSANPHFYPDG---------------KKTGLFFSEALHPFDELQAAG-----FHVDIASETGT**

**MCYG_00686 (1) ------------------------------------------------MPLPRRALIAVTSANPHFYPDG---------------KKTGLFFSEALHPFDELQSAG-----FHVDIASETGT**

**CIMG_03805 (1) -----------------------------------------------MAPLPRKALLAITSAHPPFWPDG---------------KRTGLFFSEALHPFNELTAAG-----FEVDVASETGT**

**CPC735_005110 (1) -----------------------------------------------MAPLPRKALLAITSAHPPFWPDG---------------KRTGLFFSEALHPFNELTAAG-----FEVDVASETGT**

**UREG_07480 (1) ------------------------------------------------MPLPRRALLAVTSAHPPFWPDG---------------KKTGLFFSEALHPYNELTAAG-----FEVDVASETGT**

**Mucci2_157529 (1) -----------------------------------------------MPSLPRKALIAVTSYNEPFYADG---------------TKTGLFYTEALHPYQALKKAG-----FEIDLASETGT**

**RO3G_07202.3 (1) ------------------------------------------------MSVPRKVLLAISSYNEVFYEDG---------------KRTGLFYTEALHPYQAFVQAG-----FEVDVASETGT**

**Phybl2_109595 (1) -----------------------------------------------MSSLPRKALIAITSYNEVFYSDG---------------ARTGLFYTEALHPYMALVKAG-----FEVDLASETGT**

**ACLA_067030 (1) ------------------------------------------------MPVPRRALIAVSSAHAPLYPDG---------------KETGLFITEALHPFKVFKKAG-----FEVALVSETGH**

**ANI_1_1122094 (1) ------------------------------------------------MSPARRALIAITSAHAPLYPEG---------------KETGLFITEALHPFEVFRKAG-----FEVDLVSETGT**

**NECHADRAFT_45064 (1) ------------------------------------------------MAPPKKALIAITSAHAPLYEGG---------------KETGLFITEALHPFNVFKEAG-----FETDLVSETGT**

**MGL_4192 (1) ----------------------------------------------MPASVPRRALIAVTSAHAQLYPNG---------------GETGVFITEALHPFEGFRKAG-----FEVDLVSETGK**

**FOXB_05842 (1) ------------------------------------------------MSPPRRALISITSASATLFDGK---------------ETTGLFITEALHPYKVLRAAG-----FEVDLASETGT**

**NECHADRAFT_49514 (1) ------------------------------------------------MSPPRRALISVTSAKATLFQGK---------------ETTGLFIGEALHPYRVLRAAG-----FEVDLASETGT**

**PMAA_010240 (1) ------------------------------------------------MVLPRKAVISVTSAQATLFNGK---------------ETTGVFISEALHPYHVLTAAG-----FEVDLVSETGT**

**CaO19.251 (1) ---------------------------------------------------MVKVLLALTSYNETFYSDG---------------KKTGVFVVEALHPFEVFRKKG-----YEIQLASETGT**

**CaO19.7882 (1) ---------------------------------------------------MVKVLLALTSYNETFYSDG---------------KKTGVFVVEALHPFEVFRKKG-----YEIQLASETGT**

**CD36_82570 (1) ---------------------------------------------------MVKVLLALTSYNEPFYSDG---------------KKTGVFVVEALHPFEVFRKKG-----YEIQFASETGT**

**CTRG_02436 (1) ---------------------------------------------------MVKVLLALTSEDPVFYPDG---------------KKTGVFTIEALHPFEEFKKKG-----YEVQFASETGS**

**CTRG_02543 (1) ---------------------------------------------------MVKVLLALTSANPVFYADG---------------KRTGVFVVEALHPFEVYKKKG-----YEIQFVSETGT**

**SPAPADRAFT_51238 (1) ---------------------------------------------------MVKALIAVTSYHGEFYPNG---------------GKTGLFVCEAIEPFLEFRKKG-----YEVDFASETGT**

**PICST_59509 (1) ---------------------------------------------------MPRAIIAITSYSAPFFPDG---------------KTTGLFLVEALEPYRIFKEAG-----FDVDFASETGT**

**CAGL0C00275g (1) -------------------------------------------------MAAKKVLLALTSYNGDFYEDG---------------AKTGVFVVEAMHPFNLFTEKG-----FEVDFVSETGK**

**YDR533C (1) -------------------------------------------------MAPKKVLLALTSYNDVFYSDG---------------AKTGVFVVEALHPFNTFRKEG-----FEVDFVSETGK**

**Kpol_196p3 (1) -------------------------------------------------MNQKKVLISLTSDNEVFYLDG---------------ARTGVFVVEAMHPYNAFTKKG-----FQVDFVSETGT**

**Kpol_1058p1 (1) -------------------------------------------------MAQKKVLLALTSFNGVFYADG---------------TKTGVFVVEAMHPFNAFKSKG-----FEVDFVSETGT**

**KLLA0D00682g (1) ---------------------------------------------------MTRVLIALTSYNEAFFTDG---------------AKTGVFAVEALHPFNYYKEQG-----YDVDFVSETGK**

**KLLA0D00704g (1) ---------------------------------------------------MTKVLIALTCYNDTFYADG---------------TKTGVFVVEALHPFNYYKEQG-----YDVDFVSETGK**

**YMR322C (1) -------------------------------------------------MTPKRALISLTSYHGPFYKDG---------------AKTGVFVVEILRSFDTFEKHG-----FEVDFVSETGG**

**YOR391C (1) -------------------------------------------------MTPKRALISLTSYHGPFYKDG---------------AKTGVFVVEILRSFDTFEKHG-----FEVDFVSETGG**

**YPL280W (1) -------------------------------------------------MTPKRALISLTSYHGPFYKDG---------------AKTGVFVVEILRSFDTFEKHG-----FEVDFVSETGG**

**DEHA2E03762g (1) ------------------------------------------------MSSQKKVLVAVTSYNKIFYKDG---------------KKTGLFLVEALHPFNVFKENG-----YDVDFVSETGT**

**DEHA2E17270g (1) ------------------------------------------------MLTHKKILLAVTSYNEVFYQDG---------------AKTGLFLVEALHPFEVFKDNG-----YEVDFVSETGS**

**PGUG_04657 (1) ---------------------------------------------------MVKALIAVTSYNDVFYEDG---------------AKTGLFLTEALHPFEVFRAQG-----YDVDFVSENGT**

**CANTEDRAFT_115959 (1) ---------------------------------------------------MPSVLIALPSYHGPFFNDG---------------TTTGTFAVEVIHPFEAFKAKG-----YDIDFASRTGS**

**DEHA2G01474g (1) ---------------------------------------------------MVKALIAITSYNEVFYDDG---------------AKTGLFFSEALHPFLVFKEHN-----YDVEFVSETGI**

**TDEL0F00110 (1) ---------------------------------------------------MTKVLIVLSSYNEPFYPDG---------------AKTGVFAMEALHPYQVFVKQG-----AEVDFVSETGT**

**LELG_02042 (1) ---------------------------------------------------MVKALIAITSYNEPFYDDG---------------AKTGLFVSEALEPFLEFAQKG-----YDITFASETGT**

**LELG_02043 (1) ---------------------------------------------------MVKVIIAISSYNEIFYEDG---------------QRAGALVSEILEPFVEFYEKG-----YDVTIASETGT**

**LELG_02044 (1) ---------------------------------------------------MVKVLIALTSYNESFYEDG---------------SKTGVFISEALEPFLEFFEKG-----YDITFASETGK**

**YALI0C22000p (1) -----------------------------------------------MAPPPMKALIAVTCYNDSFYPDG---------------KKTGVYFTEAYHPFMAFWAAG-----FDVQFVSEDGT**

**YALI0F00682p (1) ------------------------------------------------MSFPKRALIAVTDYNGPFYPDG---------------SKTGLFFSEAYEPFEVFQKAG-----FDVQFVSEDGK**

**SPOG_03630 (1) -------------------------------------------------MTTKKVLLATTSYYGPFYLDG---------------MKTGAYFSETLVPFKVFRKAG-----YDVQMVSESGQ**

**SOCG_04619 (1) ----------------------------------------------MSSNTTRKVLLGTTSYYGPFYLDG---------------MKTGAFFSETLVPYKVFKQAG-----YEVQIVSETGQ**

**SPAC5H10.02c (1) ----------------------------------------------MSIAKGKNALLVASSYYGPFYPDG---------------KNTGVHFSELLIPYNVFKKAG-----FNVQFVSENGS**

**SPCC757.03c (1) -----------------------------------------------MASEGK-VLLVASSYYGPFYPDG---------------MNTGVHFAELLIPYQVFREAG-----YEVQLTSETGK**

**SPOG_03829 (1) --------------------------------------------------MPGKALLVASSYYGPIYPDG---------------KNTGVHFSELLTPYEVFRQAG-----LDVDITSEKGT**

**SOCG_01701 (1) --------------------------------------------------MTGKALLVASSHYGPIYPDG---------------KNTGVHFSELLTPYQIFRQAG-----LDVDITSEKGT**

**SJAG_02988 (1) -------------------------------------------------MHGKKVLLVASSVYVPFYPDG---------------KKTGVHFSELLEPYKVFRENG-----FAVDFTSPNGS**

**SPBC947.09 (1) -----------------------------------------------MPAKTRNVLIACSDYYGPFYKDG---------------ENTGAFFLELLHPYLVFRDAC-----FNVDIVTESGK**

**SPAC11D3.13 (1) -----------------------------------------------------MVLFMKTVQR-PE-----------------------HISLKSCIPFKSLQRQGI VFRLSVRMV**

**SJAG_04674 (1) ---------------------------------------------------MPLALIIVTSYCGPFYDDG---------------KKTGAYFSEILHPYEAFTRN-----CFQVEFASETGH**

**SPAC1F7.06 (1) --------------------------------------MDERHEAAGETSEKPKVLFLLNSYYGPFYDDG------------DNTGVNVVDLYEAFKVFEENG--------FDIVIASDTGD**

**HCDG_00904 (1) --------------------------------------------------MAPKVLFVLTSQDKIPATG----------------NPTGWYLPELAHPYEVLKGK------VDLVIASPKGG**

**BDBG_02344 (1) --------------------------------------------------MAPKILIVLTSQDKIPATG----------------KPTGWYLPELAHPYDILKGK------AELVFASPKGG**

**PAAG_03106 (1) --------------------------------------------------MAPKVLIVLTSQDKIPATG----------------NPTGWYLPEFAHPYEILKDK------VELVIASPKGG**

**AFUA_5G01430 (1) --------------------------------------------------MAPKILIVLTSQDKIPSNG----------------HPTGWYLPELAHPWEVLHEK------AELVIASPKGG**

**NFIA_040680 (1) --------------------------------------------------MAPKVLIVLTSQDKIPSNG----------------HPTGWYLPELAHPWEVLHDK------TELVIASPKGG**

**ACLA_003820 (1) --------------------------------------------------MPPKVLIVLTSQDTIPANN----------------HPTGWYLPEFAHPWEVLHDK------TELVIASPKGG**

**AFLA_019490 (1) --------------------------------------------------MAPKVLVVLTSQAQIPDRD----------------HATGWYLPEFAHPWEVLHEK------VELTIASPKGG**

**ATEG_09753 (1) --------------------------------------------------MAPKILVVLTSHDQLPN-G----------------HPTGWYLPEFAHPWEVLHDK------AELVIASPKGG**

**Pc20g03290 (1) --------------------------------------------------MTPKVLVVLTSHDKLGSTG----------------NPTGWYLPEFAHPWEVLHEK------VSLTIASPKGG**

**AN6796.2 (1) --------------------------------------------------MAPKVLVVLTSFDKIEANN----------------HPTGWYLPEFAHPWEVLHSK------TELTIASPKGG**

**ANI_1_114144 (1) --------------------------------------------------MAPKVLVVLSSHDHHNANN----------------EPTGWFLPEFAHPWDVLHSK------TELVIASPAGG**

**PMAA_013250 (1) --------------------------------------------------MAPKVLVVLTSQNVIPAIN----------------HPTGWYLPEFAHPWKVLHEAG-----VDLTIASPKGG**

**TSTA_003770 (1) --------------------------------------------------MAPKVLIVLTSQNVIPNTD----------------HQTGWYLPEFAHPWKILHDAG-----VELTVASPKGG**

**FOXB_01392 (1) -----------------------------------------------MTTSKPKILVVLTSADKVPNTG----------------KQIGWYLPELAHPFHVLNPR------AELVYATPKGG**

**NECHADRAFT_81418 (1) -----------------------------------------------MTTQKPKVLVVLTSADKVPKTG----------------KQIGWYLPELAHPFHVLNPL------VELVYASPKGG**

**VDBG_07599 (1) ----------------MSNDIPTADPSRIADEGCLAPDLIAASACNNDVHNKPKVLVILTSADNHSPS-----------------------LPELAQPWEVLRNR------AELTYASPKGG**

**VDAG_09321 (1) ----------------------------------------------MASTTKPKVLVILTSVDTIPKSG----------------KTIGWYLPELAHPWEVLRNR------AELTYASPKGG**

**NECHADRAFT_83491 (1) -----------------------------------------------MAASKPRILVVLTSTDKVPTNG----------------KPIGWYLSELAHPFHVLHGK------ADFTFASPMGG**

**GLRG_10735 (1) --------------------------------------------------MAPKVLVVLTSTNKTEKSG----------------KTIGWYLPELAHPYDVLKEAG-----VEMTFASPKGG**

**MAC_07323 (1) --------------------------------------------------MAKKVLVVLTSADRIVKLDK----------------PTGWYLPELAHPYDVLAPK------AEIVVASPKGG**

**MAA_09738 (1) --------------------------------------------------MAKKVLVVLTSADKIVKLDK----------------PTGWYLPELAHPHDVLAPK------AEIVVASPKGG**

**CCM_09248 (1) --------------------------------------------------MAPKILVVLTSADKIVKLDK----------------PTGWYLPELAHPYDVLSPK------AELVLASPKGG**

**TRIREDRAFT_59940 (1) -------------------------------------------------MAPKKVLVVLTSHDKIDKINK----------------PSGWYLPEFAHPWKVLSDKN-----VEFTVVSPKGG**

**FG08979.1 (1) --------------------------------------------------MAPKVLVVLTSQSKMNNGN-----------------PTGWYLPELAHPYYDLVNAG-----VEITVASPAGG**

**NECHADRAFT_62574 (1) -------------------------------------------------MAPKKVLVVLTSRDKMDNGN-----------------PTGWYLPEFAHPYYDLVGEDESNPKVEIVVASPAGG**

**MGG_01679 (1) --------------------------------------------------MVKKVLVVLTSASEMKNKNGNA-------------KPTGWFLPEFAHPYDEFAKAG-----YELTVVSPKGG**

**CHGG_04205 (1) ------------------------------------------------MSDAPKILVVLTSHDKLGDTG----------------KPTGWYLSELAHPYEVLTSQG-----FQLTLASPTGG**

**MYCTH_2310610 (1) ------------------------------------------------MSDSPKILIVLTSHDKLGDTG----------------KPTGWYLSELAHPYSVFASHG-----FTITLASPAGG**

**THITE_2114472 (1) ------------------------------------------------MAHSPKILVVLTSHDKLGDTG----------------KPTGWYLSELAHPYQILTEHG-----FTLTLASPAGG**

**EGS21780.1 (1) ------------------------------------------------MSDSPKVLVVLTSHDKLGDTG----------------KPTGWYLPELAHPYSILASHG-----FDFDFASPKGG**

**NCU06603 (1) -------------------------------------------------MSAPKVLVVLTSHDKLGNTG----------------KPTGWYLSEFSHPYDVLNSAS-----VSLTVASPRGG**

**NEUTE2DRAFT_85759 (1) -------------------------------------------------MSVPKVLVVLTSHDKLGNTG----------------KPTGWYLSEFAHPYDVLNSAS-----VSLTVASPRGG**

**AOL_s00109g140 (1) -----------------------MATSATTAAVTAAAAPTETTTETTTKPSTVKVLVVLSSHEHLGNTG----------------KKTGWYLPEFAHPYYVLEPY------ATFTIASPRGG**

**MYCGRDRAFT_77354 (1) --------------------------------------------------MSPKVLFVLSSHDQMGNTG----------------KPTGWYLPEFAHPYYKLEGK------ADITIVSPAGG**

**PTT_13641 (1) -------------------------------------------------MSKPSILFVLTSHNKLGDTG----------------KPTGWYLPELAHPYHILRNK------ANITVASPKGG**

**PTRG_04958 (1) -------------------------------------------------MSKPSILFVLTSHNKLGDTG----------------KPTGWYLPELAHPYHVLRNK------ANITVASPKGG**

**SNOG_04306 (1) -------------------------------------------------MSKPSVLFVLTSHNKLGDTG----------------KPTGWYLPELAHPYHVLRNK------VNITVASPKGG**

**SS1G_06318 (1) -----------------------------------------------MSSTQPKVLFVLTSHDKMGNSG----------------KPTGWYLPEFAHPYDILAPH------TEITIASVIGG**

**FOXB_05365 (1) ------------------------------------------------MSTGQKLLVVLTSQDVLPTRAN---------------MKTGWYLPELVHPYNDLDGH------VELVVASPKGG**

**NECHADRAFT_60634 (1) ------------------------------------------------MSTKQKLLVVLTSQDVLPTRAN---------------MKTGWYLPELVHPYNDLAPH------VDLIMASPKGG**

**NECHADRAFT_42326 (1) ------------------------------------------------MSPKPKLLIILTSQDILPTRDN---------------MKTGWYLPELVHPYNMLVPH------VDVVIASPKGG**

**SPPG_05672 (1) ------------------------------------------------MAGKKNIVFVLTSHSQLGNTG----------------KKTGWYLPEVAHPYNILAPH------YNITWASPLGG**

**CC1G_00260 (1) ---------------------------------------------------MPSVLFVFTSTNKTLTGA-----------------QTGWYLPEAAHPYYVLA-P-----HVQIDFASPAGP**

**CC1G_11702 (1) ---------------------------------------------------MPSVLFVFISASKTLTGK-----------------PTGWYLPEAAHPYYILS-P-----HVQIDFAAPAGA**

**Pospl1_110200 (1) ---------------------------------------------------MGRILFVYTSCSRTLTGA-----------------QTGWYLPEAAHPYYVLA-A-----HHEIDFASPNGP**

**SERLA73DRAFT_120613 (1) --------------------------------------------------MAPNILFVFTSTDRTLTGA-----------------PTGWYLPEAAHPYYVLS-P-----HFNIEFAAPAGP**

**SCHCODRAFT_46162 (1) --------------------------------------------------MASKILIVFTSCDKLPNGS-----------------EAGWYLPEAAHPYYVLS-P-----SFTIDFASPKGA**

**SCHCODRAFT_49614 (1) --------------------------------------------------MSNKILFVFTSVNKTLTGE-----------------PTGWYLPEAAHPYYVLA-P-----HFDIDFAAPAGP**

**CGB_C3010C (1) -----------------------------------------------MSQSNKAILFVFTSADKLLNGA-----------------PTGWYLPEAAHPYYVLS-P-----HYRIEAISTKGG**

**CNC01950 (1) -----------------------------------------------MSQPSKAVLFVFTSAEKLLNGA-----------------PTGWYLPEAAHPYYVLS-P-----HYRIEAISTKGG**

**Rglhsp31 (1) ------------------------------------------------MAANKNILFVLTSHDKFLSGK-----------------PTGWYLPEAAHPYYVFKNA-----GYTITFASPKGG**

**Rhoba1_1_64353 (1) ------------------------------------------------MAANKNILFVVSSHDQFLDGK-----------------PTGYYLPEAAHPYYVLKNA-----GYKIDVASPKGG**

**UM00094.1 (1) --------------------------------------------------MSKKILVVFTSHTKLIGSD----------------HPTGYYLPEIAHPYYILKDAG-----YTLVSASPKGG**

**AFLA_124160 (1) --------------------------------------------------MVKRILNVVTNVGHYDDPS----------------HPTGLWLSELTHAWHVFEEHG-----FEQTIVSPAGG**

**ANI_1_1764104 (1) ------------------------------------------------MSRPKRILHVVTNVGHYDDPS----------------HPTGLWLTELTHAWDVFEASG-----YTQTIVSPAGG**

**PAS_chr3_0691 (1) --------------------------------------------------MTVQILIVVTSVAKYESGK----------------LPTGLWLSELTHMYHSAKENG-----YDVTIASPQGG**

**CLUG_02395 (1) ---------------------------------------------------MGKALIAITSYFGPFYDDG---------------AKTGLFFVEALHPYETFTQKG-----YEVTFVSEDGT**

**AFUA_3G01210 (1) -------------------------------------------------MTGKKILIILTDAKSFPLKKTSGPDAG-----KTVEQPSGFFLMELAKPLEKILAAGY-----EVTFASPKGL**

**NFIA_002150 (1) -------------------------------------------------MTGKKILIILTDAKSFPLKKTSGPDAG-----KTVEQPSGFFLMELAKPLEKILAAGY-----EVTFASPKGL**

**ACLA_063760 (1) -------------------------------------------------MSSKKILIVLSDAHSFPLKKTSGADAG-----KTVEQPSGFFLMELAKPLQKILDAGY-----EVTFASPKGQ**

**AFLA_138590 (1) -------------------------------------------------MPAKKVLIILSDADSFPLKKTSGQDAG-----KTVDQPSGFFLMELAKPLQKLLDAGY-----EVTFASPKGQ**

**ATEG_07760 (1) -------------------------------------------------MPQKKVLIILSDANSFPLKKTRGPDAD-----KVIDEPSGYFLMELAKPLEKLLAAGH-----EVTFASPKGQ**

**Pc12g09930 (1) -------------------------------------------------MPPKKVLIILSDAHSFPLKRTSGHDAG-----KVVEQPSGFFLQELAKPLHKILSAGH-----EVTFASPKGQ**

**ANI_1_1478014 (1) -------------------------------------------------MPPKKVLIILSDATSFPLHNTSSGTQ---------QQPTGFFLPELAKPLSKLLSAGH-----EVTFASPKGQ**

**AN6810.2 (1) -------------------------------------------------MPSKRILIVLSDANYFPLKKPAGSGEGSSSNSKIVDQPSGFFLMELAKPLQKLLDAGH-----EVTFASPEGR**

**TSTA_105480 (1) -------------------------------------------------MAPKKVLIVLSDAHSFPLKKPASSSN---DKDQIVQQPSGFFLMELAKPLAQLLESG--------------AP**

**CGB_E6750W (1) -------------------------------------------------MPPKKILIIMSDASAVPLLKTSQCGET------TTDQSTGFFLMELAKPLSKFLSAGC-----EVTFASPLGK**

**CNJ00030 (1) -------------------------------------------------MSPKKILIIMSDASSIPLHKTSQCGET------TADQSSGFFLMELAKPLSKFLSAGC-----EVTFASPLGK**

**PTT_19431 (1) -------------------------------------------------MAAMKVLIVLSDASSFPLYNTGSDGKT-------VSQDSGYFLMELTKPLQKVLDAGY-----EVTFASPEGK**

**PTRG_10645 (1) -------------------------------------------------MVAKKVLIVLSDASSFPLYNTGSDGKT-------VSQPSGYFLMELAKPLQKVLDAGY-----EVTFASPEGK**

**SNOG_00505 (1) ---------------------------------------MTPEQLQTYNKASKKILIIVSDASSFPLYNTGNDGKT-------VSQDSGYFLMELAKPLQKFLDAGY-----EVTFASPEGK**

**CC1G_10162 (1) -------------------------------------------------MSKGKILIILSGADSFAVEKPDGNVSE---------EKTGFFLTELATPLEKILDAGY-----DVVFASPHGN**

**Pospl1_115118 (1) -------------------------------------------------MSAGKVLIILSDADSYPVKKPDGSTTN---------QETGVFLTELAKPLQKLLDAGY-----EVTFASPSGR**

**SPPG_02734 (1) ---------------------------------------------MAQTASKGKVLIILSGANTIHLKEGK-------------DQKTGYFLSELGHPLMKILEAGY-----DVVFANPTGT**

**MAC_05717 (1) MSNTSDRAPVADQAELNAWFPSKYSLTQYTSPKTDFDGADYP---NAYTGGKWKVLLIATQERYLRMAGG-------------EFFSTGNHPVETLLPMVHLDAAG-----FDIDIATLSGA**

**MAA_08674 (1) MSNTSDRAPVADQAEFNAWFPSKYSLTQYTAPKTDFDGADYP---NAYKGGKWKILLIATQERYLKMAGG-------------EFFSTGNHPVEMLLPMVHLDAAG-----FDIDIATLSGD**

**VDBG_05153 (1) --MSNDRKPVADQAEDDTWFPSPYSLTQYVAPKTDFAEGDADYAATAYKGGKWKVLLIATQERYLKMADG-------------SFFSTGNHPVEMLLPMLHMDAAG-----FDIDIATLSGE**

**VDAG_08958 (1) --MSNDRKPVADQAEDDAWFPSPYSLTQYVAPKTDFAEGDADYAATAYKGGKWKVLLIATQERYLKMADG-------------SFFSTGNHPVEMLLPMLHMDAAG-----FDIDIATLSGE**

**CCM_05507 (1) --MSNDRAPVADTAEINAWFPSPYSLTQYVPATTDFAGTSSP---RPYTGGRWKVLLIGTQERYLPMADG-------------RFFSTGNHPVELLLPLLHLDAAG-----FDVDVATPSGG**

*****

**ARB_05407 (55) FAFDEHSLEKKFLNK-------EDEEVLHNANDPFNKKLNSQMFKAGDLSP-------HEYGLFFAAGGHGACYDFPHAKHLQAIASDVYNRGGVVAAVCHGPAILAGVKN-PE--------**

**TERG_00228 (55) FAFDEHSLEKKFLNK-------EDEEVLHNANDPFNKKLNSQMFKAGDLSP-------HEYGLFFAAGGHGACYDFPHAKHLQAIASDVYNRGGVVAAVCHGPAILAGVKN-PE--------**

**TRV_03863 (55) FAFDEHSLEKKFLNK-------EDEEVLHNTNDPFNKKLNSQMFKAGDLSP-------HEYGLFFAAGGHGACYDFPHAKHLQAIASDVYNRGGVVAAVCHGPAILAGVKN-PE--------**

**TEQG_08419 (55) FAFDEHSLEKKFLNK-------EDEEVLHNANDPFNKKLNSQMFKAGDLSA-------HEYGLFFAAGGHGACYDFPHAKHLQAIASDVYNRGGVVAAVCHGPAILTGIKN-PE--------**

**TESG_07748 (55) FAFDEHSLEKKFLNK-------EDEEVLHNANDPFNKKLNSQMFKAGDLSA-------HEYGLFFAAGGHGACYDFPHAKHLQAIASDVYNRGGVVAAVCHGPAILTGIKN-PE--------**

**MGYG_01284 (55) YAFDEHSLEKKYLNR-------EDEEVMRNSNDPFNKKLNSQMFKAGDLSP-------HEYGLFYAAGGHGACYDFPNAKHLQAIASDIYNRGGVVAAVCHGPAILGGIRG-PD--------**

**MCYG_00686 (55) YAYDEHSLEKKFLSR-------EDEEVLHNNNDPFNKKLNSQMFKAGDLSP-------HEYGLFFAAGGHGACYDFPNAKHLQAIASDVYGRGGVVAAVCHGPAILGGIHD-SN--------**

**CIMG_03805 (56) FGWDEHSLTQEYLSK-------EDEKVLHSEHNHFMEKMNKQVFKAGDLAP-------HDYGLMFVCGGHGALYDFPHAKHLQNIAQDIYKRGGVIGAVCHGPAMLPGIHD-EN--------**

**CPC735_005110 (56) FGWDEHSLTQEYLSK-------EDEKVLHSEHNHFMEKMNKQVFKAGDLAP-------HDYGLMFVCGGHGALYDFPHAKHLQNIAQDIYKRGGVIGAVCHGPAMLPGIHD-EN--------**

**UREG_07480 (55) FAWDEHSLTPSFLSK-------EDEKVYNSEQSPFMQKMNKQVFKADDLTP-------HDYGLVFVCGGHGALYDFPHAKHIQHIAQDVYKRGGVIGAVCHGPVMLPGILD-EN--------**

**Mucci2_157529 (56) YGMDEHSTEKQFLTD-------EDEKILNDPNHPFNVLLNKHLHKASELDA-------KQYGLFFASAGHATLYDYPHARGLQSIAEDIYKRGGIVSAVCHGPAILPGIKG-DD--------**

**RO3G_07202.3 (55) YGLDEHSTTKDFLTD-------EDERTYNDPNHPFNVLLNKHLYKASNLDP-------KQYGLFFASAGHATLYDYPHARGLQSIAEDIYERGGVVSAVCHGPAILPGVKDKKT--------**

**Phybl2_109595 (56) FGLDDHSTQKMFLTD-------EDEQILKNPNHPFNVKLNKELKRASDVQQHS-----KDYGLFFASAGHATLYDYPTARGLQAIAQDIYGRGGVVSAVCHGPAILPGIKDPLT--------**

**ACLA_067030 (55) YQPDWLSLQKEWLPE-------EDRAIWEDHASEFRSKLDDLL-SPSKVDA-------NNYGLFFASAGHASLIDYPDARGLQQIATQIYKDGGIVSSVCHGGAIFPGIIDTTT--------**

**ANI_1_1122094 (55) YQPDWLSQRKDWLND-------KDRAVWEDHSSEFRSKLDNLL-KPSDIDP-------EKYGLFFASAGHASLIDYPDAKGLQAIASKIFTSGGIVSAVCHGGAIFPGVIDPST--------**

**NECHADRAFT_45064 (55) CQPDWLSTTKDWLPE-------KDREVWEDHDSEFRSKVDKLM-RPSDIHA-------EDYGLFFASAGHASLIDYPEASGLQSIASKIYVDGGIVCAVCHGGAIFPNTIDPRT--------**

**MGL_4192 (57) YTPDALSMTKPWITD-------EELKVYEDKNSEFRQKLDNLK-KPSDIDP-------TKYGLFFASAGHAALIDYPDDKGLQEIASKIWEAGGIVSAVCHGGAILPGVKDQ-S--------**

**FOXB_05842 (55) YTPDWLSQQPDFLNG-------DDLAIWNDTNSEFRKKLDNMP-KASELDP-------SKYGLFYASAGHAALIDYPTASSLQNIAAQVWASGGVVSTVCHGPAIFANLIDPTT--------**

**NECHADRAFT_49514 (55) YTADWLSQQPDFLNG-------DDLVTWNDVDSDFRKKLDNMP-KAADLDG-------SKYGLFYASAGHAALIDYPTAASLQKIAAQVWANGGVVSSVCHGPAIFANLIDPTT--------**

**PMAA_010240 (55) YIADWLSQQPDFLNG-------DDLKTWNDKTSDFRKKMDNMP-KAADVDG-------SQYGVFFASAGHASLIDYPKARNLQKIAEEVWANGGVLGSVCHGPAIFANILDKAT--------**

**CaO19.251 (52) FGWDDHSVVPDFLNG-------EDKEIFDNVNSEFNVALKILK-KASDLD--P-----NDYDIFFGSAGHGTLFDYPNAKDLQKIATTVYDKGGVVSAVCHGPAIFENLNDPKT--------**

**CaO19.7882 (52) FGWDDHSVVPDFLNG-------EDKEIFDNVNSEFNVALKNLK-KASDLD--P-----NDYDIFFGSAGHGTLFDYPNAKDLQKIATTVYDKGGVVSAVCHGPAIFENLNDPKT--------**

**CD36_82570 (52) YGWDDHSVVADFLNG-------EDKEIFDNVNSDFNIALKNLK-KASDVN--P-----NDYDIFFGSAGHGTLFDYPKAKDLQKIATTVYDKGGVVSAVCHGPAIFENLNDPKT--------**

**CTRG_02436 (52) FGYDDYGISPDFLNG-------ENRKIFEDPNSDYNQALKKTK-KASDLD--P-----NDYDIFFASAGHGTLFDYPKSKSLQLIASTVYDKGGIVAAVCHGPIIFDNMKNLKT--------**

**CTRG_02543 (52) FGYDEHSTSEDFLTG-------EDKDIFEDQNSDYNVHLKNIK-KASDVS--S-----KDYDIFFGSAGHGTLFDYPKAKNLQKLAAEIYDKGGVVSAVCHGPAIFENLIDPKT--------**

**SPAPADRAFT_51238 (52) YGLDVHCTHPDYLNG-------EAKAAFDDPKSEYSVAIANIK-KASDVN--V-----DDYDIVFASAGHGTCFDYPTADGLHAIALSTYKRGGVVAAVCHGPLFFDNFNDPAT--------**

**PICST_59509 (52) FGIDDNSTQPDFLNG-------QDLEDYNNKSSDVSIALANIK-KASDITT-P-----EEYDIFYASAGHGCLFDYPKADNLHRIAATIYAKGGVVAAVCHGPAIFDNLNDLTT--------**

**CAGL0C00275g (54) YGWDEHSLIPDFLSG-------KDKEDFDNENSSFHKALKNVK-AAKDVK--A-----SDYDIFFASAGHGTLFDYPKAKELQGLAEDIYANGGVVAAVCHGPAIFDGLKDKKT--------**

**YDR533C (54) FGWDEHSLAKDFLNG-------QDETDFKNKDSDFNKTLAKIK-TPKEVN--A-----DDYQIFFASAGHGTLFDYPKAKDLQDIASEIYANGGVVAAVCHGPAIFDGLTDKKT--------**

**Kpol_196p3 (54) FGWDDHSLAPDFLSS-------QDKLDFDDKESDFNQCIRNVK-KASDIN--P-----DEYCIFFASAGHGTLFDYPTAKGLQKIASEIYDNNGVVAAVCHGPAIFDGLIEKKT--------**

**Kpol_1058p1 (54) YGWDDHSLAPDFLTG-------QDKLDFDDKGSDFNKGLKNVK-KASDVN--P-----DDYCIFFASAGHGTLFDYPKAKSLQSLASEIYDKNGVVAAVCHGPAIFDGLIEKKS--------**

**KLLA0D00682g (52) YGFDEHSLSADFLSG-------KDKEQFEDKNSAFNQALAKTK-VAADVN--P-----STYKIFFASAGHGTLFDYPTAKNLQKIASDIYANNGVVAAVCHGPAIFDGLTDKDT--------**

**KLLA0D00704g (52) YGFDEHSLSADFLSG-------KDKEQFEDKNSAFNQALAKTK-VAADVN--P-----STYKIFFASAGHGTLFDYPTAKNLQKIASDIYANNGVVAAVCHGPAIFDGLTDKDT--------**

**YMR322C (54) FGWDEHYLPKSFIGG-------EDKMNFETKNSAFNKALARIK-TANEVN--A-----SDYKIFFASAGHGALFDYPKAKNLQDIASKIYANGGVIAAICHGPLLFDGLIDIKT--------**

**YOR391C (54) FGWDEHYLPKSFIGG-------EDKMNFETKNSAFNKALARIK-TANEVN--A-----SDYKVFFASAGHGALFDYPKAKNLQDIASKIYANGGVIAAICHGPLLFDGLIDIKT--------**

**YPL280W (54) FGWDEHYLPKSFIGG-------EDKMNFETKNSAFNKALARIK-TANEVN--A-----SDYKIFFASAGHGALFDYPKAKNLQDIASKIYANGGVIAAICHGPLLFDGLIDIKT--------**

**DEHA2E03762g (55) YGFDDHSLGPDFLNG-------KDLEVFNDAKSDFNVHLKNIK-KASEVN--A-----DDYGIFFASAGHGTLFDYPQAKVLQSLGENIWSNGGVLAAVCHGGAIFDGMVDKAN--------**

**DEHA2E17270g (55) FGFDEHSLAPDFLNG-------KDLEVYKDSKSDFNQHLKNLK-KASEVN--A-----DDYGVFFASAGHATLFDYPKAKGLQSLAQAIWAKGGVVAAVCHGGAIFDGLIDQAT--------**

**PGUG_04657 (52) FGYDEHSLSPDFLDG-------DDKNIHLNAHSEFNIGLKNIK-KPSQVD--A-----SEYSIFFASAGHGTLFDYPKASGLQELAQKIYAKGGVVAAVCHGPAIFANLIDTSS--------**

**CANTEDRAFT_115959 (52) FGYDDHSLTEDFLKG-------DDKAILEDPNSEYNITLKKIK-KASDVN--P-----KDYDIFFAVGGYGASFDFPTATDVVKVAEDIYANGGVVAAVCHGPAIFTNMKDPST--------**

**DEHA2G01474g (52) VGYDEHSLSPDFLNG-------KEAEVFNDKNSDYSVSISKIK-KASDVN--A-----DDYKIIYFAGGHGTVYDFPQATGLHKIAQQIWKANGVVAAVCHGPAIFDGLNDPENP-------**

**TDEL0F00110 (52) FGWDEHSLAPDFLSG-------KEKEILEDSSSGFGKAVKQVK-KASEVN--S-----KDYDIVFAAGGHGAAYDLGRDPEVHRLAAEIYAHGGVVAAVCHGPVIFDGLKKLDG--------**

**LELG_02042 (52) FGYDEHSLSKDFLQG-------KAREVYEDKDSAYNKTIAQIK-KASDLV--D-----TKFDVFFAAGGHGAIFDFPKATDLHKIAAKTWENGNVVSAVCHGPAIFENLNLSNG--------**

**LELG_02043 (52) FGYDEHSFAEYPLHG-------KFQEAYDDQNSPYNKAIRQLK-KASDLL--N-----TDFDVFFAAGGHGAIYDLVHATNLHKIALKTFENNKVIGAVCHGPAIFANLNLSNG--------**

**LELG_02044 (52) FGYDDHSLTDHFLKG-------KLLEVYNDKNSAFNKAIAQIK-KASDLL--D-----TDFDVFFAAGGHGTIFDFPKATDLKKIAAKTWENGKVVSALCHGPAIFENLNLSNG--------**

**YALI0C22000p (56) FGYDEKSLDPINCSD-------KEMEDLRNPDSSFSKCSAKVT-TADKVN--G-----KDYGIIFFAGGHGTIHDFCDAPKLAKLASEVYFNNGILAAVCHGPAIFNNLKGPDG--------**

**YALI0F00682p (55) YGYDAHSLDPKFATE-------EQLDAQKNPQSQYNLVLDGIL-PASEID--A-----SNYSVFFAAGGHGAIFDFVNAPVLGKIAADIYAAGGIVSAVCHGPAIFASIKNEEG--------**

**SPOG_03630 (54) CRFDDHSLTETNLGE-------LEKQVLDDKNEEFWTYLKNTK-AAKDVDP-------NEYPLLFVAGGHGAMFDLPTAKGVQNLAAKVYDNKGILAAVCHGPVLLAHVKNSKCPV------**

**SOCG_04619 (57) CKFDDKSLTNIALGN-------LEKQVLDDKNDDFWECLKNAK-SAKEINP-------DEYPLLFIAGGHGAMFDLPTAKDMQNLAARIYENRGILAALCHGPVLLVHVQNTKCPK------**

**SPAC5H10.02c (57) YKFDDHSIEESKLGD-------FERKVFNDKNDDFWTNLNNMK-KASDIVG-------KDYQLLFVAGGHAAMFDLPKATNLQAVAREVFTNGGVLSAVCHGPVLLANVKNPQSVE------**

**SPCC757.03c (55) CKFDDHSIKKSALGE-------VERDAFDNKDNEFWYALKDIK-PADKINY-------KEFCIMFIAGGHAAMFDLPHATNLQTLAQQIYASNGVLAAVCHGPVMLPFVDDTKSPE------**

**SPOG_03829 (53) CHFDDNSVDESKLPE-------HVKNVLHDKSHEFWTAIKNMK-RAADVDY-------SEYNIYFVAGGHAALFDLPGAIDLQAIAAQIYKNGGVIAAVCHGPCILPFISDLTRNN------**

**SOCG_01701 (53) CKFDDVSVDESQLPA-------EIKNVLHDKSNEFWTAINNMK-RAADVDY-------SQYKIFFVAGGHAALFDLPGAIDLQAIAAQIYKNGGIISAVCHGPVILPFISDLTRPN------**

**SJAG_02988 (54) CQFDESSVDESSLPE-------NEKKILHDKQDEFWKDLKRMI-PAANVDP-------ADYCLMFVAGGHAAMFDLPTAVDLHAVAAQIYKNGGVIAAVCHGPVMLPFVQDLKSKG------**

**SPBC947.09 (56) IQFDDHSVAGPAIDKGSK(19)AEKYVLENKDDMFWRIVQNSK-TADEVNP-------DKYDIFFVAGGHATLFDFPKATNLQKLGTSIYENGGVVAAVCHGPTLLPFMKRQTSDG------**

**SPAC11D3.13 (40) MLADDHSIS--------------DSALSDSDKNAF-KDKNNDFWKAIKNAKNASDINFSDYSIFFAAGGHGTLFDFPSATNLHKGAAKIYSMGGVIAAVCHGPVILPCIKDST---------**

**SJAG_04674 (52) VGFDEHSIVPPAVTG-------EELRVLHDNSHPLMRALHSAVQRVDSLDINR-------YDIVFVAGGHGTLFDMPRSGDIQKFLAGMYEAGKIVAAVCHGPVVLPFVHLKG---------**

**SPAC1F7.06 (65) YGFDDKSFR--------------DPAIVDETQSIFSNPDCSLMKKLKNIARLD-RLNPSDYVIVYIPGGYGCSFDFPHAKVVQDFLYRFYETKGIICAVAQANIALAYTTNSD---------**

**HCDG_00904 (51) PAPLDPASVTAFAG---------DAVSSAFLKE--NESIWHNTVKLSDVKSSD-------FDAIFYVGGHGPLFDLYEDADSLRLIKEFAEARKVVSAVCHGP---AVFLKATAG-------**

**BDBG_02344 (51) LAPLDPGSVEAFAN---------DAVAAAFVKE--KEPIWRNTVKLSEVKASD-------FDAIFYVGGHGPLFDLYEDPDSLRLIKEFAESKKIVSAVCHAP---AVFLKATAG-------**

**PAAG_03106 (51) VAPLDPGSVTAFAN---------DPVASSFAKE--KEAVWSNTVKLSEVKAGD-------FDAIFYVGGHGPMFDLHNDKDSLALIQQFAELKKPVTAVCHGP---AVLLKATAG-------**

**AFUA_5G01430 (51) EAPLDPASVKMFEN---------DPVASKFLNE--QKSLWTNTVKLSDVLPKV---S--EFDAIFYVGGHGPMFDLVNDETSIALIEAFSAARKPIAAVCHGP---TVLLKAKAPS------**

**NFIA_040680 (51) EAPLDPASVKMFEN---------DPVASKFLNE--QKSLWTNTVKLSDVLPKV---S--EFDAIFYVGGHGPMFDLVNDETSIALIEAFSAAKKPIAAVCHGP---TVLLKAKAPS------**

**ACLA_003820 (51) EAPLDPASVKASEN---------DPVATKFLNE--QKSLWTNTEKLSDVLPKV---S--EFDAIFYVGGHGPMFDLHNDETSIALIEAFSEGRKTIAAVCHGP---AALIKAKTRS------**

**AFLA_019490 (51) EAPLDPSSVEAFKS---------DPVSSKFLKE--QESLWKNTHKLADFLPRV---S--EFDAIFYVGGHGPMFDLHYDETSLSLIQAFAAAGKPVSAVCHGP---TVFIKATTKS------**

**ATEG_09753 (50) EAPLDPSSVKMFEN---------DPVSTKFLNE--QSALWKNTHKLSDFVSRV---S--EFDAIFYVGGHGPMFDLHSDPVSLALIQSFAAAGKPVSAVCHGP---TVFLKATTPS------**

**Pc20g03290 (51) EAPLDPASVKMFES---------DEASQKFLKE--QKALWTNTHKLADVLPRA---G--EFDAIFYVGGHGPMFDLTEDPTSLALIQTFAAANKPVAAVCHGP---CVLLNATAPS------**

**AN6796.2 (51) AAPLDPSSVKMFEN---------DPVSARFLKE--QESLWKNTLKLEEVLPRA---EKGEFDAIFYVGGHGPMFDLVTDKTSIALIQSFAKAKKPVSAVCHGP---CVFVNVTTPS------**

**ANI_1_114144 (51) KAPLDPGSIEMFKE---------DPVSQKFLKE--QESLWTNTVKLSDVVSRV---S--EFDAIFYVGGHGPMYDLYSDKTSLALIQAFAVAKKPVAAVCHGP---AVLVNATTPS------**

**PMAA_013250 (52) EAPLDPSSVELFKS---------DEIAVSFHKN--QESLWKNTVRLADIVPRA---H--EFDAIFYVGGHGPMFDLVSDPISLSLIQTFASAKKPVSAVCHGP---IVLVNATTPS------**

**TSTA_003770 (52) EAPLDPASVEMFKQ---------DEIAVNFHKN--QEPLWKNTVRLADIVPRA---K--EFDAIFYVGGHGPMFDLVSDPISLSLIQTFASAKKPVSAVCHGP---IVFVNATTPS------**

**FOXB_01392 (54) ESPLDPVSVELFKD---------DPVCKDFLEN--HESVWKNTLKLSDVAGRA---S--EFDAIFYPGGHGPMVDLVHDEHSKNLLRDFHSQEKVISAVCHGP---AAFVNATTAS------**

**NECHADRAFT_81418 (54) VSPLDPVSVDLFKE---------DPVCKDFLEN--HTSVWEKTAKLSDFAGRA---S--EFDAIFYPGGHGPMVDLADDQHSKDLLRDFHSQGKIISAVCHGP---AALVNATTAA------**

**VDBG_07599 (78) VAPL**D**PI**S**VDL**F**SS---------**D**PVCKDFLDN--HKAIWENTQ**K**L**S**TFAGRA---S--EFDAV**F**YP**GGHGP**MF**DL**AFDEDSIALIKD**F**DAQ**GK**VIS**AVCHGP**---AAFVNAKDAA------**

**VDAG_09321 (55) VAPLDPISVDLFSS---------DPVCKDFLDN--HKAIWENTQKLSSFAGRA---S--EFDAVFYPGGHGPMFDLAFDEDSIALIKDFDAQGKVISAVCHGP---AAFVNAKDAA------**

**NECHADRAFT_83491 (54) EAPLDQVSVEMSME---------DPVCKDFHDN--HSSTWRETRRLSEVADRT---S--EFDAVFYPGGHGPMFDLISNPDSLRILRDLHAEDKVIAAVCHGP---AALVNAKTAD------**

**GLRG_10735 (52) VAPLDPASVEMFSS---------DPSSKNFLEK--HKEVWENTEPLSKFVGRA---S--EFDAIFYPGGHGPMYDLAFDADSHKLIAEFAAQNKPVASVCHGP---ASIVNAKTND------**

**MAC_07323 (51) IAPLDPSSIDMFKS---------DESSVNFLNN--NKSVWEKTTPLKEFIGRS-----DEFDALFYPGGHGPMYDLVTDQDSIKLIEEFHNAGKPVAAVCHGP---IVFRDAKA-------K**

**MAA_09738 (51) IAPLDPSSIDMFKS---------DESSVNFLNN--HKSVWEKTTPLKEFIGRS-----DEFDALFYPGGHGPMYDLVTDQDSIKLIEEFYNAGKPVAAVCHGP---IVFRDAKG-------K**

**CCM_09248 (51) VAPLDPSSVEAFKN---------DASSQKFL----EKKLWEKTAPLKDFVGRS-----GEFAAVFYPGGHGPMYDLVDDKDSIAIIEEFYKAGKPISAVCHGP---IVFAHVK--------V**

**TRIREDRAFT_59940 (53) AAPLDPSSVELFKQ---------DPVSVSFLEN--QKSLWENTLPLKQFIGRS-----GDFDALFYPGGHGPMFDLVDDADSIKLIEEFYSAGKVVAAVCHGT---IALVNAKQ-------G**

**FG08979.1 (51) EAPLDQGSVQMFKE---------DEESVKFLNE--KKQVWEQTTPLKEFLGKS-----SEFDAIFYPGGHGPMFDLVNDETSIKLIEEFYKAGKPVASVCHGP---IVFTQVK--------I**

**NECHADRAFT_62574 (57) KSPLDEVSIKMFES---------DPESVKFLNE--KKSIWENTRPLSEFLGKA-----SEFDAIFYPGGHGPMFDLVKDETSIKLIEEFYKAGKPVSAVCHGP---IVFVNVT--------I**

**MGG_01679 (55) ESKLDPASIEMFKS---------DPSSTSFLNN--QKDLWEKTKKLSDYVDKA-----SEFDAVFYPGGHGPMLDLAVDAESQKLIANFFEAGKPVAAVCHAP---IVLADVKL-------S**

**CHGG_04205 (54) PAPLDPSSIEAASN---------DDSSQRFLKEQ--NALWEATEPLISFLG---RA--DEFAALFFPGGHGPMFDLAVDPTSQALVQEFADKGKVVAAVCHGP---AALVGAGK--------**

**MYCTH_2310610 (54) PAPLDPSSIEAAAASS-------DAVSQSFLAEQ--RALWESTEPLASFVG---RA--AEFAAVFFPGGHGPMFDLATDPTSQALVREFAAAGKVLAAVCHGP---AALANVRTGGDDAGQ-**

**THITE_2114472 (54) AAPLDPASVDAASS---------DPVSTAFLAQHRAAPPWERTAPLASFLG---RA--DDFAALFFPGGHGPMFDLAADPACQALVAEFAAKDKVVAAVCHGP---AALLGVKISSGAAGGG**

**EGS21780.1 (54) PAPVDPASVENADE---------E--SKAFYAKN--SSLWEDTAPLQSFLG---RA--DEYAALFYPGGHGPVFDLVENPTSQTLIQEFLAKGKVVAAVCHGV---AALLGVQDPESADG--**

**NCU06603 (53) LAPVDPSSIEAAKD---------DQVSQNFIKSETTKPLFEQTRPLSEFVGNPSAI--APYSAIFFPGGHGPMYDLATSSESQQLIREFWDAGKTVAAVCHGP---AALVNVKLSDG-----**

**NEUTE2DRAFT_85759 (53) LSPVAPGSIEDAKD---------DEISQNFIKSETTKPLFEQTRPLSEFVGNPSAI--APYSAIFFPGGHGPMYDLPTSPESQQLIREFWEAGKAVAAVCHGP---AALVNVKLSDG-----**

**AOL_s00109g140 (78) PSPLDPSSVEAFKN---------DPISQKFLAE--KEELWTRTEMLFKFLGKA---N--LFDALFYVGGHGPMFDLAIDATSQALIKEFYEKGKIVSAVCHGP---AAFVNVKLID------**

**MYCGRDRAFT_77354 (51) KAPLDPSSVEMFKE---------DAESQKFLKE--KSALWENTEKLSDYIGKA---S--QFDAIFYVGGHGPMFDLATDEKSHQLIREFYEAGKVVSAVCHGP---AALAKVKLSD------**

**PTT_13641 (52) EAPLDPASVEASKD---------DVSVN-FLKN--DEQVWKTTQKLADFKGKA---K--DFDAIFYVGGHGPMFDLVDDATSQQLIREFWEADKIVSAVCHAP---SVFYDAKLSD------**

**PTRG_04958 (52) EAPLDPASVEASKD---------DVSVN-FLKN--DEQVWKTTQKLADFKGKA---K--DFDAIFYVGGHGPMFDLVDDATSQQLIREFWEADKVVSAVCHAP---SVFYDAKLSD------**

**SNOG_04306 (52) EAPLDPASVEAAKD---------DVSVN-FLKN--DEQVWKNTQKLSDFSGKA---K--DFDAVFYVGGHGPMFDLADNPTSQQLIKEFWEAGKIVSAVCHAP---AVLYDVKLSD------**

**SS1G_06318 (54) ASPLDPASIEASRD---------DVSVN-FLKT--QESLWKNTVPLSDFVGKA---A--EFDAIFYVGGHGPMFDLAHNETSQKIISEFHSLNRVIAAVCHGP---AALAYARISS------**

**FOXB_05365 (54) EAPVDPYSIEDSKN---------DEASQRFFKE--KWHVWKNTQKLESFLGKS---A--EYVGIFFVGGHGPMFDLAVDPTSHALIREFYESNKLVSAVCHGP---AALVNVKLSD------**

**NECHADRAFT_60634 (54) EAPIDPYSIEDAKN---------DEACQVFLKE--KGHVWKNTQKLESFLGKS---D--EYAGIFFVGGHGPMFDLAVDGTSHALIREFYESGKLVSAVCHGP---AALANVKLSD------**

**NECHADRAFT_42326 (54) EAPIDPYSVEETKD---------DAACQAFLRD--NEPLWKNTTKLESFLGRS---S--EFAGIFFVGGHGPMFDLAVDSISQELIREFYEAGKIVSAVCHGP---AALVNVKLSD------**

**SPPG_05672 (53) ETPLDPSSAEAFAK---------DEECIKFLNDPVAQKGVKETVKVEDILARA---N--EFDAVFFPGGHGPMFDLFTFVPSLQLAAKVYENNGVVAAVCHGP---AAIVNIKLSN------**

**CC1G_00260 (49) NPPIDEGSVKAFEKDA---------ESVKFLNNETVKQKLASAHKLSDINVAN-------YDAIFYVGGHGPVIDLASDPVNGKLVSDFWNAGKIVSAVCHGPAHCSALVQGADKD------**

**CC1G_11702 (49) NPPIDEYSVKSYTDDG---------S-VKFLNDETVEQKLANAKKLVNIDYKE-------YDAVFYVGGHGPAIDLASDAVNARLVSDFWKSGKIVAAVCHGP---AALVQGVDEH------**

**Pospl1_110200 (49) NPPVDEGSVEMFKDDE---------S-VKFLQDATVTEKLATAKKLSEVNPSQ-------YDAVFYVGGHGPVLDLATDPTNIKVGNEFWRSGKITSAVCHGP---AALVGVTDAE------**

**SERLA73DRAFT_120613 (50) NPPVDPASVQMFQDD------------VKFLEDANVQSLLAKAKKLTDVKVDD-------YEAIFYIGGHGPVIDLAVDPANIKLASEFYRAGKLTTAVCHGP---AALVGATDAS------**

**SCHCODRAFT_46162 (50) NPPVSQGSVELFKEDA---------ESVQFLADEIVKQKLASAKILSEVNPDD-------YAAVFYVGGHGPCIDLPNDETNIKLANSFWRAGKIVSAVCHGP---AALVRITDAS------**

**SCHCODRAFT_49614 (50) NPPLDPVSKEMFQDE----------ESQKFLTDKVPQEKLANAKKLADVNPDD-------YAAVFYVGGHGPVIDLATDPTNVKLASKFWQQGKIVSAVCHGP---AALVGATDAS------**

**CGB_C3010C (53) PVPVDQHSVENFQDE----------ESQKFLKDPDAQKLVKNTKKVEDVKAAD-------YEAIFVIGGHGPLIDLAKSEKFAKLVEDFYVAKKPVSAVCHGPG--ALILATNPTT------**

**CNC01950 (53) PVPVDETSVKNFQDE----------DSQKFLKDPEAQNLVKNTKKVEDVKAAD-------YEAMFVIGGHGPLIDLAKSEKFAKLVEDFYVAKKPVSAVCHGPG--AFILATNPAT------**

**Rglhsp31 (53) KAPLDPSSVEMFKEDE---------EATKFLNDPEAKQLYENTKKLVDVKADD-------YASVLYVGGHGPVL-LTTNQDSIALIHSFLSANKPIAALCHAP--TVLLNCQDPKT------**

**Rhoba1_1_64353 (53) KAPLDPSSVEAFKEDS---------DSIKFLNEDEPKQLFANTKKITDVKEAD-------YAALALPGGHAPIFDLTTDKDSIALIESFLKAGKPVASVCHGP--TVFLNVTDPKS------**

**UM00094.1 (52) KAPLDQSSVDAFKD---------DADSVKFLNDTEAQDWVNNTKRLSEFSASS----VSEFDAIFYPGGHGPCFDLPVDTTSQELIKTFYEAGKPTSAVCHAP---AVFADVKLSD------**

**AFLA_124160 (52) PCPLEPRSLKFPNY---------DKTAKAWHADPARMALLENTASPDQINSAD-------FDAIYFTGGHAVMYDFPDSEGLQRITREIYERGGIVSSVCHGY---CGLLNTKRSD------**

**ANI_1_1764104 (54) HCPLEPRSLKFPNV---------DRTAKAWQADVERMALLQNTFSPEQIKSAE-------FDAIYFTGGHGVMYDFKDSEGLQRITREIFERGGVVASVCHGC---CGLLNTTLSD------**

**PAS_chr3_0691 (52) NIPLDPESLKSMLI---------DKLSKDYETNQDFMKLLQNTKSLGEVTGQQ-------FDVVYLAGGHGTMYDFPNNTVLQNIIKEHYEAGKIVAAVCHGV---CGLLNVKLSD------**

**CLUG_02395 (52) FGWDEHSVAEAFLPAG------KDREIYEDKNSTFMKAISNVK-KPSEVN--A-----DDYDIFFAAGGHGTTFDFPKASGLHELAAKIWSNNKVVAAVCHGPLIFS-NLLVDG--------**

**AFUA_3G01210 (64) EPTPDPLSESLAAFAGNFYERRRENDLIDRMKRENGFSRPRPLGTISDAELDS-------FAGVFIPGGHAPLSDLGSDKEVGRVLRHFHAKKKPTASICHGPIAFLSTKQAGDG-------**

**NFIA_002150 (64) EPAPDPLSESLPAFAGNFYERRRENDLIDRMKRENGFSRPRPLGTISDAELDS-------FAGVFIPGGHAPLSDLGGDKEVGRVLRHFHAKKKPTASICHGPIAFLSTKQAGDG-------**

**ACLA_063760 (64) EPAPDPNSESLLAFAGNFYERRRENELIERMKRENGFAKPRPFSTISDGELDS-------FAGVFIPGGHAPLKDLGADKELGRVLRHFHEKSKPTAAICHGPYAFLSTKQAGDG-------**

**AFLA_138590 (64) EPTPDPNSESLLAFAGNFYERRRENELIDRMKRENGFSHPRTFSSISDDELES-------FAGVFIPGGHAPLRDLGADKDLGRILRYFHAKSRPTAAICHGPFAFLSTKFAGDG-------**

**ATEG_07760 (64) EPAADPNSETLLAFAGNFYERNREKDLIERMKRENGFSHPRPFSSISDDELAT-------FAGVFIPGGHAPLSDLGTDKELGRILRYFHKENKPTAAICHGPYALLSTKYAGDG-------**

**Pc12g09930 (64) EPAPDPSSESLIANAGSFYERQRGNDLIERMKRENGFSSPRPFSTISDDELTT-------FAAVFIPGGHAPLKDLGGDAELGRILRHFHEENKPTAVICHGPYALLSTKKAGDG-------**

**ANI_1_1478014 (60) TPQPDPNSESLLTFAGNFYERRREQELIERMRRENGFSSPRPFASISDDELKT-------FAAVFIPGGHAPLVDLGGDKELGRILRYFHGENKPTAAICHGPLALLSTRVSGDG-------**

**AN6810.2 (69) EPQPDPNSESLLAFAGNFYERRRENELLERMKKENGFTKPRKLNSISDDELKN-------FAGVFIPGGHAPLADLGDNKDLGRILEYFHKENKPTAAICHGPYALLSTKVSG-G-------**

**TSTA_105480 (57) NPLQTPTVETLLAFAGNFYERRHENELIDRMRRENGFDSPRPFKSISDEELDS-------FAGVFIPGGHAPLQDLGDDPELGRILKHFHNKTKPTAAICHGPYAFLSTKATSPG-------**

**CGB_E6750W (63) TPTPDPNSESLMAFALNFYERKRENDLIDRMKRENGFMSPRTFQSISDEELDG-------FAAVFIPGGHAPLADLGADPELGRILAHFHREGKPTAVICHGPYGLLSTKATPEG-------**

**CNJ00030 (63) TPTPDPTSESLVAFALNFYERKRENDLIARMMRENGFASPRTFQSISDEELDG-------FAAVFIPGGHAPLADLGADPELGRILAHFHHEGKPTAVICHGPYGLLSTKATPEG-------**

**PTT_19431 (62) EPTPDPLSVSLAAFAGNYYEKQRELDLIERMKKENGFSRPRKFSEISDDDLKS-------YSGVFIPGGHAPLSDLGDNPELGRILTHFHSAQKPTAALCHGPWAFLSTKYTPNKP------**

**PTRG_10645 (62) EPTPDPLSVSLAAFAGNYYEKQRELDLIERMKKENGFSRPKKFSEISDDDLKS-------YSGVFIPGGHAPLSDLGDNPELGRILTHFHSAQKPTAALCHGPWAFLSTKYTPNKA------**

**SNOG_00505 (72) EPTPDPNSLSLMAFAGNFYERQRELDLIERMKKENGFSRPRKFSEISDDQLKN-------FGGVFIPGGHAPLSDLGDNADLGRILSHFHQNAKPTAALCHGPWAFLSTKYGPQK-------**

**CC1G_10162 (60) KPSIDPLSESILVYMGNYWRKKKEEELIERMRVEKSLYAPRAFSEIGDDELRG-------FKGVFIPGGHAPLTDLGADPELGRILLHFYRNGKPTAAVCHGPYALLSTRVAPNST------**

**Pospl1_115118 (60) RPNIDPLSESLVVYFGNWLQKNRDNQLIQRMYAENNLALPRPFPSITDDELES-------YAGVFIPGGHAPIRDLGNNPDLGRILWHFHGRGKPTAAICHGPLALLSTKYARDSP------**

**SPPG_02734 (60) HPVQDPISELLIWFLGNYKEREREHALIDKMKIESNFASPRPFASLTDDDLST-------FVGVFIPGGHAPMQDLHNDKELARILGHFHRMAKPTGAICHGPAALLSTKTEEGC-------**

**MAC_05717 (102) PVKFEVWAFPKEDEAV--------KKIYAKYEDKLRSPLNLQDIWGKDG-----FTSETPYLGIFIPGGHGALNDVPFSKLVGDIIRWAHDNQRYYLTLCHGPASMLAANVGKPEG------**

**MAA_08674 (102) PVKFEVWAFPKEDEAV--------KKIYAKYEDKIRSPLNLQDIWGKDG-----FTTETPYLGIFIPGGHGVLNDVPFSKLVGDIIRWAHDSQRYYLTLCHGPASMLAANVGKPDG------**

**VDBG_05153 (103) PVKFEMWAFPKEDKAE---------------------------VWGQG------FTDATPYLAVFIPGGHGVLNGVPFSATVGDVLRWAHAHERFFVTLCHGPASMLAADVGKPAG------**

**VDAG_08958 (103) PVKFEMWAFPKEDKAV--------QAIYDKYRDKIRNPLNLQDVWGRG------FTADTPYLAVFIPGGHGVLNGVPFSATVGDVLRWAHAHERFFVTLCHGPASMLAADVGKPAG------**

**CCM_05507 (100) PVKLEVWAFPSRDEAV--------TQIYKKYEEKLRAPLSLEALWEGSSSNGG-FDASTPYLAVFVPGGHGVLNDIPRSRLVGDVLRWAHAQDRYVVTLCHGPAALLAAEVGAPAG------**

******

**ARB_05407 (154) -EEPVVKDKTVTGFTTEGELEL-KVIDQMRQDKVHTIEDCLAPTG--AHYEAPASAFDNFEKVDG----------RIVTGANPASARDTARDAIKVFDGLD-------------------**

**TERG_00228 (154) -EEPVVKDKTVTGFTTEGELEL-KVIDQMRQDKVHTIEDCLGPTG--AHYEAPPSAFDNFEKVDG----------RIVTGANC-------------------------------------**

**TRV_03863 (154) -EEPVVKDKTVTGFTTEGELEL-KMIDQMRQDKVHTIEDCLAPTG--AHYEAPSSAFDNFEKVDG----------RIVTGANPASARDTARDAIKVFDGLD-------------------**

**TEQG_08419 (154) -EEPVVKDKTVTGFTTEGELEL-KVIDQMRQDKVHTIEDCLGPTG--AHYEAPPSAFDNFEKVDG----------RIVTGANPASARDTARDAIKVFDGLD-------------------**

**TESG_07748 (154) -EEPVVKDKTVTGFTTEGELEL-KVIDQMRQDKVHTIEDCLGPTG--AHYEAPPSAFDNFEKVDG----------RIVTGANPASARDTARDAIKVFDGLD-------------------**

**MGYG_01284 (154) -DEPVVKDKTVTGFTTEGELEL-KVIDQMRQDNVHTIEDCLKPSG--ARYEAPPTAFENFEKIDG----------RIITGANPASARDTARDAIKVFDGLE-------------------**

**MCYG_00686 (154) -DEPIVKNKTVTGFTTEGELEL-KVIDQMRQDKVHTIEDCMGPTG--ARYEAPPTPFENFERIDG----------RLVTGANPASARDTARDAIKVDAPMQFLLQENSSH----------**

**CIMG_03805 (155) -GDSVIKDKTVTGFTTKGEIMI-KVIDKMREDHLHTIADMAQTAN--AEYVPPEDPWDDFCKVDG----------RIVTGANPQSATNTARDTIKVYEGIVNE-----------------**

**CPC735_005110 (155) -GDSVIKDKTVTGFTTKGEIMI-KVIDKMREDHLHTIADMAQTAN--AEYVPPEDPWDDFCKVDG----------RIVTGANPQSATNTARDTIKVYEGIVNE-----------------**

**UREG_07480 (154) -GDSIIKDKTVTGFTTNGELML-KVIDQMRNDKLHTVAELATNAH--AEYVEPGDPFDNFCKVDG----------RVVTGANPQSATHTALDTIKVFEGIAKE-----------------**

**Mucci2_157529 (155) -GKSIINGKTVTGFTNEGEVQL-NILDKIKDDKVPTIEEAAASVG--ATYVAPPTPFADFNKTDG----------RVVTGANPASAHSTAEAAIAAFEKL--------------------**

**RO3G_07202.3 (155) -GKSIIDGKTVTGFTSLGEEQM-NLMQKIKGDKVLTIEEGAADAG--ATYVSPPTPFADFHKIDG----------RVVTGANPASAHSTADAAIKVFDGK--------------------**

**Phybl2_109595 (158) -GKSVVENKVVTGFTDLGEVQM-KVMDKLRSDKLNTVEKWMELSH--AKYIAPPEPFNCFSKIDG----------RIVTGANPASAKLTAENAIKVFDCQ--------------------**

**ACLA_067030 (154) -GKPIIAGRRVTGFTTKGEEEE-GVLDTIKSWNRPTIEKSAADCG--ATYVSPPGPWDAFTITDG----------RIVTGANPASATVTAEAAVAAFDKL--------------------**

**ANI_1_1122094 (154) -NKSIIDSRRVTGFTTRGEEEE-NVLDTIKSWNRPTIEASAASCG--ATYVSPPGPWDAFTITDG----------RVVTGANPASAHVTAEAAVTAFDKL--------------------**

**NECHADRAFT_45064 (154) -GKSIIAGKKVTGFTTKGEEEE-GVLDTIKSWHRPTIEAAAADAG--ATYISPPGPWDAFTHTDG----------HIVTGANPASAHVTAEAAVKAFDML--------------------**

**MGL_4192 (155) -GKSIISGRKITGFTDKGEEEL-GALAEIKNWNRPTIQESAASAG--AEYVHPPGPWAPYQVTDG----------RLVTGVNPQSATVTTEAAIKAFDAL--------------------**

**FOXB_05842 (154) -NEPLIKGKKITGFTTEAENTM-KIMGELRSWGSEMVEEVAARLG--ATYERAPGIWDDFHVVDG----------RLVTGQNPASATSTAEAAVAVFEKL--------------------**

**NECHADRAFT_49514 (154) -NEPLIKGKKITGFTTEAEYTL-KIMDELRSWNTEMVEEVAARLG--ATYQRAPGIWDDFHVVDG----------RLVTGQNPASATSTAEAAVAVFETP--------------------**

**PMAA_010240 (154) -GEPVIKGKKLTGFTTEAEYTM-QIMDDLKSWNSELVEELAARLG--AKYERSAGIWDDFHVVDG----------RLVTGQNPASATSTAKAVVEVFNKL--------------------**

**CaO19.251 (151) -GEPLIKGKKITGFTDIGEDIL-GVTDIMKKGNLLTIKQVAEKEG--ATYIEPEGPWDNFTVTDG----------RIVTGVNPQSAVKTAEDVIAAFECN--------------------**

**CaO19.7882 (151) -GEPLIKGKKITGFTDIGEDIL-GVTDIMKKGNLLTIKQVAEKEG--ATYIEPEGPWDNFTVTDG----------RIVTGVNPQSAVKTAEDVIAAFECN--------------------**

**CD36_82570 (151) -GEPLIKGKKITGFTDVGEDIL-GVTDLMKKDNLLTIKQVAEKEG--ATYIEPEGPWANFTVTDG----------KIVTGVNPQSAVKTAEDTITAFESK--------------------**

**CTRG_02436 (151) -GEPLIKGKKITGFTDIGEEIL-GCCRYKKEITLLTMRQVAEKEG--ATYIEPSGPWESFTVTDD----------RIVTGVNPQSAEKITEATIAAFEAK--------------------**

**CTRG_02543 (151) -NEPLIKGKKITGFTDVGEDML-GVTDIMKKDNLLTIKQVAEKEG--ATYIEPEGPWDNFTVTDG----------KIVTGVNPQSAAKTAEESIAALSA---------------------**

**SPAPADRAFT_51238 (151) -GEPIVKGKKITGFTDEGEEYL-GVFNIMKEKNLETVNQMAQKLG--ATYVAPNGPWDAFAVVDG----------KIVTGVNPQSAVVTAEKTIETYEAK--------------------**

**PICST_59509 (152) -GKPIIQGKTITGFTDVGETIL-QVDQIMKDLKLETIRETAEKLG--AIYKEPEGPFVDFSLIDG----------KVVTGVNPQSAKSTAENAIKALKA---------------------**

**CAGL0C00275g (153) -GKPLIEGKAITGFTDIGEVVL-NVDKIMKDKGLLSVEDIAKKYG--AKYLAPIGPWDDFSITDG----------KLVTGVNPASAHSTAQRAIDALN----------------------**

**YDR533C (153) -GRPLIEGKSITGFTDVGETIL-GVDSILKAKNLATVEDVAKKYG--AKYLAPVGPWDDYSITDG----------RLVTGVNPASAHSTAVRSIDALKN---------------------**

**Kpol_196p3 (153) -GKPLIEGKAITGFTDIGEVIL-KVHGVMKEKNLLSVEDIAKKYN--AKYLPPIGPWDDYSVTDG----------KLITGVNPA------------------------------------**

**Kpol_1058p1 (153) -GKPLIEGKVITGFTDIGEVIL-QVDGIMKEKSLLSVEDIAKKYN--AKYVPPIGPWDDYSVTDG----------RLITGVNPASAHSTAVRAMDAAKQ---------------------**

**KLLA0D00682g (151) -GKPLIQGKVITGFTDIGEEIL-GVADILKQKKLDTVEDIAKKYH--AKYMAPIGPWDDFSIADG----------RLVTGVNPASASSTAKRTDQVLNSLN-------------------**

**KLLA0D00704g (151) -GKPLIQGKVITGFTDIGEEIL-GVADILKQKKLDTVEDIAKKYH--AKYMAPIGPWDDFSIADG----------RLVTGVNPASASSTAKRTVQVLNSLN-------------------**

**YMR322C (153) -TRPLIEGKAITGFPLEGEIAL-GVDDILRSRKLTTVERVANKNR--AKYLAPIHPWDDYSITDG----------KLVTGVNANSSYSTTIRAINALYS---------------------**

**YOR391C (153) -TRPLIEGKAITGFPLEGEIAL-GVDDILRSRKLTTVERVANKNG--AKYLAPIHPWDDYSITDG----------KLVTGVNANSSYSTTIRAINALYS---------------------**

**YPL280W (153) -TRPLIEGKAITGFPLEGEIAL-GVDDILRSRKLTTVERVANKNG--AKYLAPIHPWDDYSITDG----------KLVTGVNANSSYSTTIRAINALYS---------------------**

**DEHA2E03762g (154) -GKPLLQGKSITGFTDIGEAIL-GVDGIMKENELQSIEEMTKKYG--GKYLAPIGPWDDYSVSDG----------KLITGVNPASAISTAKRSLIAFEAI--------------------**

**DEHA2E17270g (154) -GKPLIEGKSITGFTDLGEIML-KVDGIMKENNLLSVEDVAKKFG--AKYLAPIGPWDDYTVADG----------KLITGVNPASAASTAKRSLIAVKHKHT------------------**

**PGUG_04657 (151) -GRPIVEGKTVTGFTDKGEEQM-QVDKIMKSKNLESVEELYKRLG--AKYSAPNGPWDDHSVVDG----------RVVTGVNPQSANSTAKKAVQVLSEVKGIDKVTG------------**

**CANTEDRAFT_115959 (151) -GKPLIAGKNVTGFTDEGEKLF-DTDKPLVKYNLDTMEGVAKKTG--ANFKLPGAPFDDFTVTDG----------RVVTGVNPASAGSTARAAIIASQQ---------------------**

**DEHA2G01474g (152) -NKLLVEGKTITGFTNAGEEIM-GVVETLKKKNLGSIEDLSTRLG--AKFSPPSAPFEEHSIAEG----------RLVTGANPASASSTAVKAIKALSS---------------------**

**TDEL0F00110 (151) --ELLIKDRKLTGFTDEGEVIM-KVDKLMEEKGMKTVKQIAKING--GCYVQPKDPWESFVVVDG----------KIVTGVNPASAGETATKSLAAFGQ---------------------**

**LELG_02042 (151) --EPLIKGRKITGFTDIGEEQL-GVQDIIKKDNLLTVPQVAKKEG--AEYVEPKGPWDNFTVSDG----------KLITGVNPQSAVETARKIFVAVESVNN------------------**

**LELG_02043 (151) --QPLIKGKQITGFPDAGEEEL-GLEEIIKKDKVLTIAEIAKKEG--ANFIEPKDTWSNFTVVDG----------NLFTGANPASAVELAKKISAAIDSI--------------------**

**LELG_02044 (151) --EPLIKGRKITGFTDIGEEQL-GLGNLIKKSNLSTVAQVAEREG--AEYVAPNGPWDNFTVTDG----------NLVTGVNPQSAIDTAKKIAAVVESAST------------------**

**YALI0C22000p (155) --KPLIQGKTITGFHDQGETDM-GLDEILKQRSLPTIRQIAANVG--ANYQAPPDPWKDFSLSDQ----------RIVTGANPASATSTAIKAIELYNKL--------------------**

**YALI0F00682p (154) --QPIVAGKVITGFTAEGEKEM-GVSEAITKHGKKLVPEIAKEAG--AEYRAPPTPFQDFSITDD----------RIVTGANPASANSTAEKVVQLFDAIDAPTQSGASVLK--------**

**SPOG_03630 (155) -GKSVVYGKNVTAFTHAGEVMM-GLSTSMRNHNIGFLNEILEQAE--AKYINPPTPVSDFVQVDG----------RIVTGVNPQSAKSTAEAAINTLESKST------------------**

**SOCG_04619 (158) -GKSVVYDKNVTAFTHAGEVMM-GLSTPLKNHNIGFLNEILEQAG--ARYINPSTPVSDFVQVDG----------RIVTGVNPQSAESTAKAAVETLKNNST------------------**

**SPAC5H10.02c (158) -GKTVVYHKHVTAFNKAGEEKM-GVMDELKKRGMKSLNEIFAEAG--ATFIDPPNPNVNFTQIDG----------KIVTGVNPQSAKSTAEAAVSAL-----------------------**

**SPCC757.03c (156) -GRSVVYGKKVTAFNSTGELVM-GVSSALRERNMQDLNSLFREAG--AEFVDPPTPMSDFTQVDG----------RIVTGVNPMSAKSTAEAAIKVSQSLRKT-----------------**

**SPOG_03829 (154) -ASSIVEGRNVTAFNKAGEESM-NMLDIMQKKNLETMNDAFRKAG--AKFIDPASPNDDFVQSDS----------RVVTGVNPQSAASTAKAALQALKA---------------------**

**SOCG_01701 (154) -ASSIVQGRKVTAFNKAGEESM-NVLDIMKQKHLQTMNDLFKDAG--AHFVDPPKPTDDFVQSDS----------RIVTGVNPQSAASTAKAALEASKA---------------------**

**SJAG_02988 (155) -EVSIVKGKHVTAFSKQGEQAM-GVMNQMKQHHFRTLNEVFNSAG--ANFIDPPDPMGEFVQSDC----------RVVTGVNPASAIATAKTAVQALS----------------------**

**SPBC947.09 (160) -SVSIVCGKDVTAFDRVAEDKS-KLMEALKKYNLEVLDDMLNDAG--ANFIKSPNPFGDFVIADG----------RLVTGSNPASATSTAKTALRVL-----------------------**

**SPAC11D3.13 (138) –GFSIVKGKTVTAFNEIAEQQM-NLMPTFEKYHFKTLNKLFQEAG—-SNFVDPQEPFDDFVKTDG----------KLVTGANPASAASTAKAALNSLNS---------------------**

**SJAG_04674 (151) -GTPLVEGKRITGFTRKGEEMVG-VMGTMQKHNFKTIEDLAESAG--AVFKQKEDPFEEYVVLDG----------HLVTGTNPASARRTAEVAVEAWKRVETHACVY-------------**

**SPAC1F7.06 (163) –GQALCTNRRVTGCTWKDEVQN-GVLNVMNRLNFYSFGHIAENIG—-AIFESPPVYVEDPFIVE---------DGQLFTGSNTNSAKGVAMEAVRAVLNYDG**

**HCDG_00904 (145) -GKPVLAEAQATGFSNEEEDQ----VKMSQYLPYALETEMNKISN--GGYVKADLPWGEKVVVAKVAA----TGAPLITGQNPASATAVGTEILKALGL---------------------**

**BDBG_02344 (145) -GKPIVADAQVTGFSNAEEDQ----IKMSQHLPYLLETELKKLN---GKYVKADEPWGEKVVVDKVEA----TGAPLITGQNPASAGGVGKEILKALGL---------------------**

**PAAG_03106 (145) -GKPLIADAKVTGFSNTEEAA----VKMAQYMPFKLETELNKVSN--GGYVKADEPWAEKIVVSRVAA----TGAPLITGQNPASASGVGKEILRALGL---------------------**

**AFUA_5G01430 (148) -GVPLLSACTVTGFSNTEEDQ----AQMSSAMPFMLEDELQKVTG--NRYVKADEPWGEKVVVSKAAG----SGATVITGQNPASATGVGKEILRALGVA--------------------**

**NFIA_040680 (148) -GVPLLSACTVTGFSNTEEDQ----AQMSSAMPFMLEDELQKVTG--NRYVKADEPWGEKVAVSKAAG----TGATVITGQNPASATGVGKEILRALGVA--------------------**

**ACLA_003820 (148) -GVPLLSAATVTGFSNAEEDQ----AQMSSVMPFMLEDELQKVTG--HRYVKADEPWGEKVVLSKTAG----TATTLITGQNPASATGVAKEILRALGLA--------------------**

**AFLA_019490 (148) -GQPLLANSTVTAFTNVEEDQ----AQLTALMPYLVEDELNKIPG--CKFVKADQPWGEKVVVSKTS-----DGATLITGQNPASATGVGKEILKALGL---------------------**

**ATEG_09753 (147) -GEPLIAGASVTGFSNTEEDQ----VNLSSLMPYMLETELNKVSG--GGYVKADQPWGEKVVVSKTAG----SGAPLITGQNPASATAVGQALVQALGL---------------------**

**Pc20g03290 (148) -GVPLISGVGVTGFSNAEEDA----VSLSAAMPFMLETELGRVSG--GKYVKAAEPWGEMVVVGKAAG----TGSTIITGQNPGSATGVGKEILKALGL---------------------**

**AN6796.2 (150) -GKPLVADAEVTGFSNVEEDQ----VDLSKVMPFMLEDELNKKSG--GKYVKADQPWGEKVVVSQVKE----LGGPLITGQNPASATGVGKALLEALGA---------------------**

**ANI_1_114144 (148) -GTALLKGAEVTAFSNTEEDQ----VQLSSIMPFMLEDELKR-VG--ATFVKAEQPWAEKVVVSQVAE----LGGAVITGQNPASATGVGKAILTALGL---------------------**

**PMAA_013250 (149) -GEPLLRDAKVTGFSNTEEDL----AQMTSAMPFLLEDRLRAIPG--ATYVKANEPWGVKVVVDKTYQ----LGGVLITGQNPASATGVGMELVKALGL---------------------**

**TSTA_003770 (149) -GEPLLRDATVTGFSNVEEDQ----AQLTKLMPFLLEDRLKAIPG--TKYVKADQPWGEKVVVDKTYQ----LGGVLITGQNPASATGVGKELLKALGL---------------------**

**FOXB_01392 (151) –GELILKGKQVTGFDDVGEEM----FKFTEDMDFSLERRLGEASG--GKYVKAEEGPLAEKVVVDG---------KMITGQNPASSKGVAEEIAKALGVY--------------------**

**NECHADRAFT_81418 (151) -GEPLLKGVRVTGFDNVGEEM----FQFTEDMDFSLEDRLGQVSG--GNYVKADEGPLAEKVVVNG---------KIITGQNPASSKGVAEEIAKALEV---------------------**

**VDBG_07599 (175) -GEPLLKGKTVTAFTNEGEDM----FKYTEEMNFSLEDKLNEVSG--GKFVKSEEGPMGEKVVVDG---------RIITGQNPASSKGVGEEIAKALGVF--------------------**

**VDAG_09321 (152) -GEPLLKGKTVTAFTNEGEDM----FKYTEEMNFSLEDKLNEVSG--GKFVKSEEGPMGEKVVVDG---------RIITGQNPASSKGVGEEIAKALGVF--------------------**

**NECHADRAFT_83491 (151) -GEYIIQGREVTGFDDVGEEM----FGFDEDVGFSLEGLLNERSG--GKYSKADDGPLGKKVIVDG---------KLITGQNPASAYDVGLELAKALGV---------------------**

**GLRG_10735 (149) -GEYLVKGKRVTGFSNTEEDQ-----AGYTAEMNFLLENRLKENG--GKYESADAPWGEKVVVDG---------IVITGQNPASALGVGKAIVKALGL---------------------**

**MAC_07323 (148) SGEPLLKGKNVTGFTNVEEDQ----VQLSAAMPFLLEDELKKVG---ANFHAADQPWGEKVVVDG----------LIISGQNPASAKGVGEAIAKAIGV---------------------**

**MAA_09738 (147) SGEPLLKGKNVTGFTNVEEDQ----VQLSAAMPFLLEDELKKVG---ANFHAADQPWGEKVVVDG----------LIISGQNPASARGVGEAIANAIGV---------------------**

**CCM_09248 (144) DGKPLVEGRTVTGFTNEEEAQ----VGLAEAMPFLLEDRLKAVG---AKWTQADQAWGEKVVVDG----------QIITGQNPASAHAVGVALAKAIGI---------------------**

**TRIREDRAFT_59940 (149) NGEPLLKGKEVTGFTNAEEEA----VQLTSAMPFLLEDRVKAVG---ANFVQTD-AFGNKVVVDG----------TLITGQNPASATELGEAIAKAIAA---------------------**

**FG08979.1 (146) DGKPLLEGREATGFSNSEEDA----VGLTSAMPVLLEDEIKRVG---GKYVKAD-DWAEKLAVDG----------QIITGQNPASAHAVGKAILKAIGA---------------------**

**NECHADRAFT_62574 (152) DGKPLLQGRTVAGFSNSEEEA----VQLTSAMPALLEDEIKRVG---GNYVKGE-DWGEKLAVDG----------LVITGQNPASAHAVGKALAKAIGI---------------------**

**MGG_01679 (151) TGEYLVKGKTVTGFTNKEEEI----MQLVDYMPFLLEDKVKASG---ASFVKADEPWGEKVVVDG----------KLITGQNPASAKALGEALVKVLG----------------------**

**CHGG_04205 (149) ----VLEGKAVTGFTNAEEEA----VGLDGVVPFLLEDRLVEAVGKGGRFEKAAEKWGEKVVVDG----------KLITGQNPASAKGVGEAIARAVWGA--------------------**

**MYCTH_2310610 (158) AGEFLLRGRRVTGFTNAEEAA----VGLDRAVPFLLEDRLRELVGSEGAFEKADADWGEKVVVDG----------KLITGQNPASATALAEAIVRAVLGSA-------------------**

**THITE_2114472 (159) AGEAYLRGKRVTGFSNAEEEE----IGLGGVVPFLLEDRLTEVVGKGGKYVKAAEKWAEKVVVDG----------KLITGQNPASAKGVAEAIVEAVAGA--------------------**

**EGS21780.1 (153) --TPYLKSKAVTGFSNAEEED----VGLASAVPFLLEDKLIEAVGTNGSYEKAEDKWAQKVVVDG----------KLITGQNPASAKGVAEAIVRAVWGA--------------------**

**NCU06603 (156) --SHLLKGRKVTGFTNDEEDQ----VGLTKVMPFLLEDKLKEAVGTEGAFEKAEQPWAEKVVVDAG--------GRLITGQNPASAKGVGEAIVKSIKAQN-------------------**

**NEUTE2DRAFT_85759 (156) --SHLLKGRKVTGFTNVEEDQ----VGLTQVMPFLLEDKLKEAAGPEGAFVKADQPWGEKVVVEAD--------GRLITGQNPASAKGVGEAIVKFIKAQK-------------------**

**AOL_s00109g140 (175) -GEHLLQDQPVTGFSNYEEEV----VGLTKAMPFLLEDALDKASR--RKFEKAEEPWAPHVAVGRGG--------RLITGQNPASATPLGEELLKQLGIST-------------------**

**MYCGRDRAFT_77354 (148) -GSYLVNGAEVTGFSNTEEDQ----VQLSSVMPFMLEDELKKNGG---KYVKADEPWGAKVVSSGNHGKLLTGRGTILTGQNPNSAGPIGEAILKAL-----------------------**

**PTT_13641 (148) -GSLLIAGKEVTAFTNAEEEQ----AGLTKAVPFLPEDALNKASG--GKFVKAAEPWGEKVVVS----------GRLITGQNPASATGVAEAIAKALGI---------------------**

**PTRG_04958 (148) -GSLLIAGKEVTAFTNAEEEQ----AGLTKAVPFLPEDALNKASG--GKFVKAAEPWGEKVVVS----------GRLITGQNPASATGVAEAIAKALGI---------------------**

**SNOG_04306 (148) -GSLLVAGKEVTAFTNAEEEQ----VGLSKVVPFLPEDALNKASG--GKFVKAAEPWGEKVVVV----------GNLITGQNPASATGVGEAIAKALKV---------------------**

**SS1G_06318 (150) -GKYFLEDTPVTGFSNTEEEN----YGVIDFMPFELETALNEASN--GKYEKAAGNFAAKVVVARD--------GKVITGQNPASASGVGEAILAELRKERKCA----------------**

**FOXB_05365 (151) -GTYMVQGQTVTGFSNAEEDA----YKFTDAMPFLLEDKLKEHGG---QYEKADQLFGVKVVTSGKS-------KNLITGQNPPSAGVIGKVLLQAIQKGMV------------------**

**NECHADRAFT_60634 (151) -GTYMVDGQTVTGFSNAEEEA----YKFTDAMPFLLEDKLQEHGG---HYEKADQLFGVKVVTSGKS-------ENLITGQNPPSAGVIGKVLSEAIRKRAV------------------**

**NECHADRAFT_42326 (151) -GTYMVQGHAITGFSNAEEDA----YQFTDAMPFLLEDELKKHGG---NYEKADQPFGIRVVVSG----------NLVTGQNPPSAGVIGGSLVEEIQRREA------------------**

**SPPG_05672 (152) -GDSLVKGKRVTSFSNSEEDA----VQLSPVMPFLLENALKSAGA---KYEKAAKDWEAHVVVDG----------RLITGQNPNSGAPLGEKLRELLG----------------------**

**CC1G_00260 (149) -GKSIFAGRSFTGFSNAEEKA----VDKVNDVPFLLEDKITS-LG--GKYEKAAEPWGPKVVVDG----------RLITGQNPASASGVGEAILKAIKGN--------------------**

**CC1G_11702 (145) -GQSIFKGKKFTVLSNDEEVA----INGVNEIPFSPEDKIIE-LG--GIFEKADELFEAKTVVDG----------KLVTGQNPASSAGVGEAILKLIKGDQRLRE---------------**

**Pospl1_110200 (145) -GKSIFTGKAATGFSNTEEEQ----VNKVKDIPFLLESRIKE-LG--GKYEAAAEPWGVKVVVDG----------KLITGQNPASAKAIGEAIHKALA----------------------**

**SERLA73DRAFT_120613 (144) -GKSIFAGKAFTGFSNVEEEQ----VGKVKDIPFLLEDKITS-LG--GKYEKATAPWGAKVVVSG----------HLITGQNPASAQPIGEAILKALKS---------------------**

**SCHCODRAFT_46162 (147) -GQSIFKGRRATCFTVAEEKI----LGTVDAIPFQPEETLKE-LG--AQFEN-AGPFESKVVVDG----------RLLTGQNPASSRALAEEVLKALKG---------------------**

**SCHCODRAFT_49614 (146) -GQSIFKGRRATCFTYDEELQ----VKKVDSIPFQPEHKIVE-LG--GKFEK-TEPWGVKVVADG----------QLYTGQNPASAGPLGQELLKALKK---------------------**

**CGB_C3010C (150) -KKSIFAGACATGFSNSEEAQT-PYNDFVNILPFSLEDKIKE-LG--GKYEKANQDWGVKVIWDQ----------GILTGQNPASAGPLAVKLKEILEA---------------------**

**CNC01950 (150) -RRSIFAGARVTGFSNSEEAQT-PYNDFVNILPFSLEDKIKE-LG--GQYEKADQDWGVKVIWDQ----------GVLTGQNPASAGPLAVKLKEILEA---------------------**

**Rglhsp31 (150) -GEALVKGKKVTCFSDEEEKQ----AGLVDEIPFLVETRLRA-LG--ADFQNSRQPWGEEVCEDG----------LVITGANPASAGAMAKKLLARLGN---------------------**

**Rhoba1_1_64353 (151) -GEPFVKGKKITCFSDDEERQ----AGLVDSIPFLVETELRN-KG--ADFQLTRKAWAEEVVVDG----------QLITAGNPASASGMAKALLTVLEKA--------------------**

**UM00094.1 (152) -GSYLVNGKKITCFTNQEEEQ----AGLTKAIPWLVESRLIERG---ATFEKAEP-WAEKVIVDKTGG------RVLISGQNPASAAAVGRQILAALQA---------------------**

**AFLA_124160 (149) -GSYLVAGQEMTGFSWCEEVLA-R---VDKSVPYNAEEELKK-RG--AHYKKATLPFVSYTVVDG----------NLVTGQNPGSATETAKKVVAALNRS--------------------**

**ANI_1_1764104 (151) -GSYLVAGRKLTGFSWREELLA-R---VNKLVPYNVEEELKK-RG--ARYMKATLPFTSYAVNDG----------NLVTGQNPGSAKETAKKVVKVLSGS--------------------**

**PAS_chr3_0691 (149) -GEYLIKDKAITGFNWFEEAIA-G---RRKEVPFNLEAELNK-KT--SKYEKAFIPMTSKVVVDG----------NLITGQNPFSSKEIAKVVMEQLKQ---------------------**

**CLUG_02395 (151) --KPLIENKKVTGFTDEAEAIM-KLDGTMKKYGLKTVKDIAQECG--ATYVEPADPWSSYTVTDG----------KLVTGVNPASAAECAEKSIAALN----------------------**

**AFUA_3G01210 (172) --SFIYKGYKITCWSDAEEKV----METMFGGEVKKVESSLRNEG--AEMVEGPREKVGNITVDR----------ELVTGGNPLAANALGDQFLKMLSVH--------------------**

**NFIA_002150 (172) --SFIYKGYKITCWSDAEEKV----METMLGGEVKKVESSLRNEG--AEMVEGPREKVGSITVDR----------ELVTGGNPLAANALGDQFLKMLSVH--------------------**

**ACLA_063760 (172) --SFVYKGYKITCWSDAEEKV----METLMGGEIEKVESRLRDEG--AEMVEGVMEKIGSITVDR----------ELVTGANPMAANVLGDQFLKMLSAH--------------------**

**AFLA_138590 (172) --EFAYKGYKITSWSDAEEKM----MEMMMGGEIEKVESVLRNEG--AVMIEGAKEKIGSITVDR----------ELVTGANPTAANALGDQFLQMLNVH--------------------**

**ATEG_07760 (172) --EFAYKGYKITSWSDAEEKL----MEMMLGGGIEKVASDLAAEG--AIMVEGAKEKVGGTTLDH----------ELLTGGNPVAADALGERFLRMISAY--------------------**

**Pc12g09930 (172) --SFVYNGYKITSWSDAEENL----METLWCGEVEKVESTLRNEG--AVMVEGVREKTGGTTLHR----------ELVSAGNPVAASALGDRFVRMISV---------------------**

**ANI_1_1478014 (168) --EFVYKGYEITCWSDAEEKV----METLLRGEIEKVESQLRDAG--AVMVEGGREKLGKTTLCR----------ELLTGANPLAAEELGERFVRMVSV---------------------**

**AN6810.2 (176) --EFAYKGYKITSWSNAEEKV----MESMLGGEVEKVETALMNAG--AEMVEGAKEKVGQTTLHR----------ELLTGGNPMAADELGNRFVKMISV---------------------**

**TSTA_105480 (165) --EFAYKGYKLTSWSDAEERM----METMLGGEIEKVESSLKDSG--AEMVAGAREKAGYITVDK----------EVVSGGNPLAADALGKQFLKMLSEKA-------------------**

**CGB_E6750W (171) --TFLYKGYKITSWSDAEEKL----METLWGGEVPKVESTLREAG--AEMVEGLGEKIGRITVDR----------ELISGGNPLAANALAEQILKMVEAQ--------------------**

**CNJ00030 (171) --TFLYKGYKITSWSDAEEKM----METLWGGEVPRVESTLREAG--AEMVEGLGEKIGRITVDR----------ELISGGNPLAANALAEQVLKMVEAQ--------------------**

**PTT_19431 (171) --EFAYKGYKMTSWSDAEEKF----METIMRGQIDKVESTLREEG--ADMQEGIAKSMGSITVDR----------ELITGDNPMSANAIGDKFVEMMAAK--------------------**

**PTRG_10645 (171) --EFAYKGYKMTSWSDAEEKF----METIMRGEIDKVEFTLREEG--ADMQEGIAKSMGSITVDR----------ELITGDNPMSANAIGDKFVEMMAAK--------------------**

**SNOG_00505 (180) --EFAYKGYKMTSWSDAEEKM----MEVYFRGEITKVEGTLREEG--ADMQEGLAKSMGSITVDR----------ELITGDNPMAANAIGDKFLEMLAGVK-------------------**

**CC1G_10162 (169) --GFAYKGYKLTSWSDASESL----VEKLKGGHIEKVESALRDAG--ADMQTQTSKNLGSVTVDR----------EVVSGANPLAVAELGEKFVEMLAQKQGEEQIESLERRNRFPVQVN**

**Pospl1_115118 (169) --GFAYKGYKLTSWSDAEERL----VERLQGGEIPKVESTLQAEG--AEMVSTVGKKAGGITVDR----------EVVSGANPMAAEGLGSKLIEMLRA---------------------**

**SPPG_02734 (168) ----PYSGYKVTAYSNTEDKL----NEIMWWGTLPYKLVDELEKA--GLKCEETFPMGSKVTVDR----------ELVSGQNPSSASAFGDAFVKKLNESVTLGGTGVHAKEI-------**

**MAC_05717 (205) -SKYIYDGYEIVVFPDSLDTGANIDMGYIPGKMPWLMGEELKKLG----VKPLNEDMTGQVHRDR----------FLLTGDSPLASNALGKLAATTLLEDVAKRG---------------**

**MAA_08674 (205) -SKYIYDGYEIVVFPDSLDTGANIDIGYIPGKMPWLVGEELKKLG----VTPLNKGITGQVHRDR----------FLLTGDSPLASNALGKLAATTLLEDVAKRG---------------**

**VDBG_05153 (186) -SKYIYEGYKVDVFPDALDQGANIDIGYIPGKMEWLVGERLRALG----VEPINSQITGEVHRDR----------LLLTGDSPLASNNLGKLAAKTLLEAVANK----------------**

**VDAG_08958 (205) -SRYIYEGYKVDVFPDSLDEGANIDIGYIPGKMAWLVGERLRALG----VEPINSQITGEVHRDR----------LLLTGDSPLASNNLGKLAAKTLLEAVASK----------------**

**CCM_05507 (207) -SRFLYDGYEVVAFPDALDQGTNIDIGYIPGKMQWLVGEALRKRG----VKTLNEGITGRVHRDR----------LLLTGDSPLASNALGKLAAETLLEAVAKRG---------------**

*****
