# Supplementary material for: Identification of glutathione (GSH)-independent glyoxalase III from Schizosaccharomyces pombe
Source: BMC Evol Biol. 2014 Apr 23;14:86. doi: 10.1186/1471-2148-14-86 (PMC4021431; doi:10.1186/1471-2148-14-86)
Supplement: Additional file 6 — Candidate fungal Hsp31 proteins predicted to localize to nucleus or mitochondria. Predicted NLSs are shown. “+” indicates the predicted mitochondrial localization. [file 1471-2148-14-86-S6.doc]

Additional file 6. Candidate fungal Hsp31 proteins predicted to localize to nucleus or mitochondria

| Species | Systematic name | Predicted nuclear  localization signal | Mitochondriallocalization |
| --- | --- | --- | --- |
| *Aspergillus fumigatus* | AFUA_3G01210 | ^148^KKKP^151^ |  |
| *Aspergillus nidulans* | AN6810.2 | ^107^KPRK^110^ |  |
| *Aspergillus niger* | ANI_1_1764104 | ^3^RPKR^6^ |  |
| *Coprinopsis cinerea* | CC1G_10162 | ^79^RKKK^82^ |  |
| *Neosartorya fischeri* | NFIA_002150 | ^148^KKKP^151^ |  |
| *Pyrenophora teres* | PTT_19431 | ^100^RPRK^103^ |  |
| *Pyrenophora tritici-repentis* | PTRG_10645 | ^100^RPKK^103^ |  |
| *Schizosaccharomyces japonicus* | SJAG_02988 | ^71^KKILHDKQDEFWKDLKR^87^ |  |
| *Coccidioides immitis* | CIMG_03805 |  | + |
| *Coccidioides posadasii* | CPC735_005110 |  | + |
| *Mucor circinelloides* | Mucci2_157529 |  | + |
| *Malassezia globosa* | MGL_4192 |  | + |
| *Magnaporthe oryzae* | MGG_01679 |  | + |
| *Nectria haematococca* | NECHADRAFT_49514 |  | + |
| *Nectria haematococca* | NECHADRAFT_83491 |  | + |
| *Penicillium marneffei* | PMAA_010240 |  | + |
| *Postia placenta* | Pospl1_110200 |  | + |
| *Schizosaccharomyces pombe* | SPAC11D3.13 |  | + |
| *Uncinocarpus reesii* | UREG_07480 |  | + |

“**+**” indicates mitochondrial localization
