# Supplementary material for: Identification of glutathione (GSH)-independent glyoxalase III from Schizosaccharomyces pombe
Source: BMC Evol Biol. 2014 Apr 23;14:86. doi: 10.1186/1471-2148-14-86 (PMC4021431; doi:10.1186/1471-2148-14-86)
Supplement: Additional file 7 — Multiple sequence alignment of candidate S. pombe Hsp31 proteins. [file 1471-2148-14-86-S7.doc]

Additional file 7

Hsp3104 (1) ------------------------MVLFMKTVQRPEHISLKSCIPFKSLQ

Hsp3105 (1) MDERHEAAGETSEKPKVLFLLNSYYGPFYDDGDNTGVNVVDLYEAFKVFE

Hsp3102 (1) -----------MSIAKGKNALLVASSYYGPFYPDGKNTGVHFSELLIPYN

Hsp3101 (1) ---------MASEGK----VLLVASSYYGPFYPDGMNTGVHFAELLIPYQ

Hsp3103 (1) ------------MPAKTRNVLIACSDYYGPFYKDGENTGAFFLELLHPYL

*****

Hsp3104 (27) RQGIVFRLSVR----MVMLADDHSIS----------------------DS

Hsp3105 (51) ENGFDIVIASDT---GDYGFDDKSFR----------------------DP

Hsp3102 (40) VFKKAGFNVQFVSENGSYKFDDHSIE----------------------ES

Hsp3101 (38) VFREAGYEVQLTSETGKCKFDDHSIK----------------------KS

Hsp3103 (39) VFRDACFNVDIVTESGKIQFDDHSVAGPAIDKGSKGEEFLSYDDHIASGP

Hsp3104 (51) ALSDSDKNAFKDKNNDFWKAIKNAKNASDINFSDYSIFFAAGGHGTLFDF

Hsp3105 (76) AIVDETQSIFSNPDCSLMKKLKNIARLDRLNPSDYVIVYIPGGYGCSFDF

Hsp3102 (68) KLGDFERKVFNDKNDDFWTNLNNMKKASDIVGKDYQLLFVAGGHAAMFDL

Hsp3101 (66) ALGEVERDAFDNKDNEFWYALKDIKPADKINYKEFCIMFIAGGHAAMFDL

Hsp3103 (89) ELSKAEKYVLENKDDMFWRIVQNSKTADEVNPDKYDIFFVAGGHATLFDF

Hsp3104 (101) PSATNLHKGAAKIYSMGGVIAAVCHGPVILPCIKDST------GFSIVKG

Hsp3105 (126) PHAKVVQDFLYRFYETKGIICAVAQANIALAYTTNSD---GQALCTNRRV

Hsp3102 (118) PKATNLQAVAREVFTNGGVLSAVCHGPVLLANVKNPQSVEGKTVVYHKHV

Hsp3101 (116) PHATNLQTLAQQIYASNGVLAAVCHGPVMLPFVDDTKSPEGRSVVYGKKV

Hsp3103 (139) PKATNLQKLGTSIYENGGVVAAVCHGPTLLPFMKRQTSDGSVSIVCGKDV

******

Hsp3104 (145) KTVTAFNEIAEQQMNLMPTFEKYHFKTLNKLFQEAGSNFVDP-QEPFDDF

Hsp3105 (173) TGCTWKDEVQNGVLNVMNRLNFYSFGHIAENIGAIFESPPVYVEDPFIVE

Hsp3102 (168) TAFNKAGEEKMGVMDELKKRGMKSLNEIFAEAGATFIDP-PNPNVNFTQI

Hsp3101 (166) TAFNSTGELVMGVSSALRERNMQDLNSLFREAGAEFVDP-PTPMSDFTQV

Hsp3103 (189) TAFDRVAEDKSKLMEALKKYNLEVLDDMLNDAGANFIKSPNPFGDFVIAD

Hsp3104 (194) VKTDGKLVTGANPASAASTAKAALNSLNS---------------------

Hsp3105 (223) DGQLFTGSNTNSAKGVAMEAVRAVLNYDG---------------------

Hsp3102 (217) DGKIVTGVNPQSAKSTAEAAVSAL--------------------------

Hsp3101 (215) DGRIVTGVNPMSAKSTAEAAIKVSQSLRKT--------------------

Hsp3103 (239) GRLVTGSNPASATSTAKTALRVL---------------------------
