# Supplementary figures and images for: Identification of glutathione (GSH)-independent glyoxalase III from Schizosaccharomyces pombe
Source: BMC Evol Biol. 2014 Apr 23;14:86. doi: 10.1186/1471-2148-14-86 (PMC4021431; doi:10.1186/1471-2148-14-86)

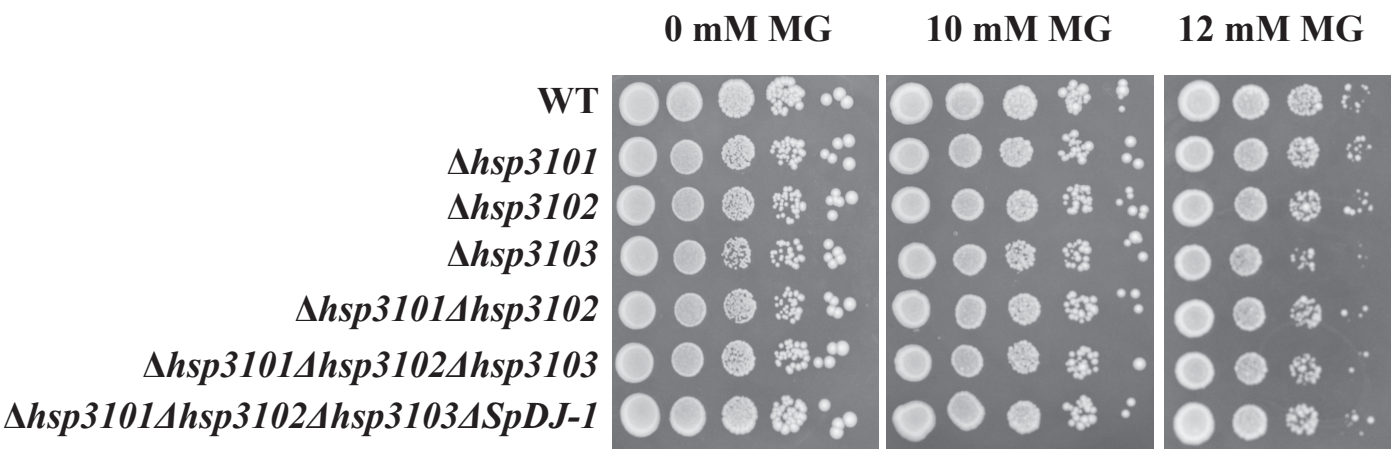

Supplement: Additional file 8 — Deletion of individual SpDJ - 1 and hsp 3101-3103 or in combination does not affect the MG sensitivity of cells. [file 1471-2148-14-86-S8.pdf]
