# Supplementary material for: Identification of glutathione (GSH)-independent glyoxalase III from Schizosaccharomyces pombe
Source: BMC Evol Biol. 2014 Apr 23;14:86. doi: 10.1186/1471-2148-14-86 (PMC4021431; doi:10.1186/1471-2148-14-86)
Supplement: Additional file 9 — List of S. pombe strains used in this study. [file 1471-2148-14-86-S9.doc]

Additional file 9. List of *S. pombe* strains used in this study

| Strain | Genotype | Source |
| --- | --- | --- |
| yHL6381 | *h^+^ his3-D1 leu1-32 ura4-D18 ade6-M210* | H. Levin |
| yAS56 | *h^+^* *leu1-32* *ura4-D18* | H. Levin |
| ySY1 | *h^+^ his3-D1 leu1-32 ura4-D18 ade6-M210* Δ*SpDJ-1::kanMX6* | This study |
| ySS1 | *h^+^ his3-D1 leu1-32 ura4-D18 ade6-M210* Δ*Spglo1*::*ura4^+^* | This study |
| yTW1 | *h^+^ his3-D1 leu1-32 ura4-D18 ade6-M210* Δ*hsp3101::his3* | This study |
| ySY4 | *h^+^ his3-D1 leu1-32 ura4-D18 ade6-M210* Δ*hsp3102::kanMX6* | This study |
| yWP1 | *h^+^ his3-D1 leu1-32 ura4-D18 ade6-M210* Δ*hsp3103:: kanMX6* | This study |
| ySY2 | *h^+^ his3-D1 leu1-32 ura4-D18 ade6-M210* Δ*hsp3101::his3* Δ*hsp3102::kanMX6* | This study |
| ySY3 | *h^+^ his3-D1 leu1-32 ura4-D18 ade6-M210* Δ*hsp3101::his3* Δ*hsp3102::kanMX6*  Δ*hsp3103::ura4* | This study |
| ySY4 | *h^+^ his3-D1 leu1-32 ura4-D18 ade6-M210* Δ*hsp3101::his3* Δ*hsp3102::kanMX6*  Δ*hsp3103::ura4* Δ*SpDJ-1::leu1* | This study |
| yCP1 | *h^+^ his3-D1 leu1-32 ura4-D18 ade6-M210 SpDJ-1-GFP::kanMX6 Sptrz1-RFP::leu1* | This study |
| yCP2 | *h^+^ his3-D1 leu1-32 ura4-D18 ade6-M210 hsp3101-GFP::kanMX6 Sptrz1-RFP::leu1* | This study |
| yCP3 | *h^+^ his3-D1 leu1-32 ura4-D18 ade6-M210 hsp3102-GFP ::kanMX6 Sptrz1-RFP::leu1* | This study |
